# Chemical Constituents from *Cimicifuga dahurica* and Their Anti-Proliferative Effects on MCF-7 Breast Cancer Cells

**DOI:** 10.3390/molecules23051083

**Published:** 2018-05-04

**Authors:** Chu Thi Thanh Huyen, Bui Thi Thuy Luyen, Ghulam Jilany Khan, Ha Van Oanh, Ta Manh Hung, Hui-Jun Li, Ping Li

**Affiliations:** 1 State Key Laboratory of Natural Medicines, China Pharmaceutical University, Nanjing 210009, China; thanhhuyen0187@hotmail.com (C.T.T.H.); cpuli@163.com (H.-J.L.); 2 Hanoi Univerisity of Pharmacy, 13–15 Le Thanh Tong, Hanoi, Vietnam; luyenbthoaduoc@gmail.com (B.T.T.L.); havanoanh@gmail.com (H.V.O.); 3 State Key Laboratory of Bioelectronics, School of Biological Science and Medical Engineering (BME), Southeast University, Nanjing 210096, China; U4574904@hotmail.com; 4 National Institute of Drug Quality Control (NIDQC), 48 Hai Ba Trung, Hoan Kiem, Hanoi, Vietnam; tahungvkn@gmail.com

**Keywords:** *Cimicifuga dahurica*, lignan, phenolic amide, triterpenoid glycoside, anti-proliferation

## Abstract

This study was designed to search for novel anti-cancer compounds from natural plants. The 70% ethanolic extract from the rizhomes of *Cimicifuga dahurica* (Turcz.) Maxim. (Ranunculaceae) was found to possess significant *in vitro* anti-proliferative effects on MCF-7 breast cancer cells. A phytochemical investigation using assay-guided fractionation of the ethanolic extract of *C. dahurica* resulted in the isolation of one new phenolic amide glycoside **3**, two new lignan glycosides **4** and **7**, one new 9,19-cycloartane triterpenoid glycoside **6**, and thirteen known constituents **1**, **2**, **5**, and **8**–**17**. The structures of **3**, **4**, **6**, and **7** were established using contemporary NMR methods and from their HRESIMS data. The anti-proliferative effects of isolated compounds were evaluated using the BrdU-proliferation kit. Five among the 17 isolated compounds showed significant anti-proliferative effects (*p* ≤ 0.05), wherein compound **7** showed the most significant anti-proliferative and cell cycle arresting effect (*p* ≤ 0.05) which followed a dose dependent manner. Western blot protein expression analysis showed a down expression of c-Myc and cyclin D1 which further elucidated the anti-proliferation mechanism of compound **7** while apoptotic effects were found in association with Bcl-2 family protein expression variations. Conclusively this study reports the isolation and identification of 17 compounds from *C. dahurica*, including four novel molecules, in addition to the fact that compound **7** possesses significant anti-proliferative and apoptotic effects *in vitro* that may require further exploration.

## 1. Introduction

The genus *Cimicifuga* (now known as *Actaea*) has been widely used all over the world and since ancient times has been a traditional Chinese herbal medicine. This genus consists of 28 species worldwide [1], among which nine species are indigenous to China. Three *Cimicifuga* species, namely, *C. dahurica*, *C. foetida*, and *C. heracleifolia*, commonly known as “Shengma” in Chinese, are officially listed in the Chinese Pharmacopoeia [2]. In traditional Chinese medicine, Shengma is widely used for treating aphtha, sore throat, toothache, and wind-heat headache. It also has been used in archoptosis, non-erupting measles, spot poison, uterine prolapse, and other diseases [1,2,3,4]. Pharmacological studies have revealed that *Cimicifuga* possesses anti-menopause symptoms [5], antitumor [6], anti-inflammatory [7], anti-osteoporosis [8] and some other effects. In addition to a lot of researchs about biological activities, the safety of *Cimicifuga* rhizomes were also conducted [9]. To date, more than 450 compounds including 9,19-cycloartane triterpenoids, phenylpropanoids, chromones, lignans, amides and other compounds have been isolated from *Cimicifuga* spp. plants [1]. Among them, many compounds such as triterpenoids [10,11,12,13] and phenolic compounds [14,15] show potent anti-cancer activities. In continuation of our studies seeking novel anti-cancer agents from natural products, we focused on assessing the effects of compounds from the ethanolic extract of *C. dahurica*.

## 2. Results and Discussion

### 2.1. Characterization of Compounds ***1**–**17***

Compound **3** was obtained as white powder. The HR-ESI-MS of **3** exhibited the ion peak at *m*/*z* 506.2020 [M + H]^+^, which was in good agreement with a molecular formula of C_25_H_31_NO_10_ (M = 505.19480). The low-field region of the ^1^H-NMR spectrum showed the benzene ring signals of six protons at *δ*_H_ [7.12 (br s, H-6), 7.17 (overlapped, H-2 and H-5)], and [6.83 (d, *J* = 1.5 Hz, H-2′), 6.75 (d, *J* = 8.0 Hz, H-5′), 6.68 (dd, *J* = 1.5, 8.0 Hz, H-6′)] which coupling features suggested that the structure contained two ABX spin systems (Table 1). The other two olefinic protons at *δ*_H_ 7.47 (d, *J* = 15.5 Hz, H-7) and 6.50 (d, *J* = 15.5 Hz, H-8) were a typical pair of *trans*-double bonds, while peaks at *δ*_H_ 3.84 (s) and 3.88 (s) indicative of the presence of two methoxy and two methylene groups at *δ*_H_ 2.79 (t, *J* = 7.5 Hz, H-7′) and 3.51 (t, *J* = 7.5 Hz, H-8′) were also observed. The carbinol protons signal (*δ*_H_ 3.70 to 5.32) and ^13^C-NMR spectra (*δ*_C_ 62.8 to 100.2) suggested the presence of an allose moiety in the structure of **3** (Table 1).

**Figure 1:**
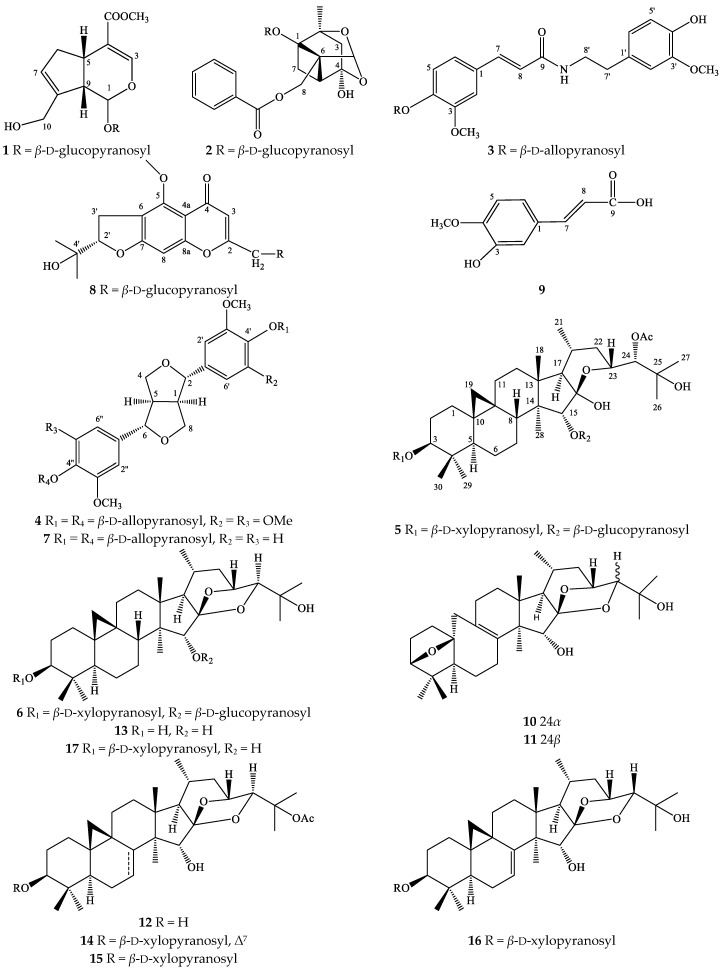
Structures of compounds **1**–**17** isolated from *C. dahurica*.

Complete assignments of all carbons and protons were based on HSQC, COSY, and HMBC experiments. After acid hydrolysis of **3**, the absolute configuration of the sugar unit was confirmed using TLC, based on a sugar standard and analysis of the coupling constant of H-1″ (*J* = 8.0 Hz). Thus, the sugar moiety was identified as *β*-d-allopyranoside. The position of an allose sugar and two methoxy groups were determined by HMBC experiments. The HMBC correlations between H-1″ (*δ*_H_ 5.32) and C-4 (*δ*_C_ 149.7), H-2 (*δ*_H_ 7.17) and H-6 (*δ*_H_ 7.12) with C-4 suggested the location of the allose at C-4. Two methoxy groups attached at C-3 and C-3′ were determined based on the HMBC correlations of the methoxy protons (*δ*_H_ 3.88) with C-3 (*δ*_C_ 150.9) and H-5 (*δ*_H_ 7.17) with C-3, the methoxy protons (*δ*_H_ 3.84) with C-3′ (*δ*_C_ 148.9) and H-5′ (*δ*_H_ 6.75) with C-3′, respectively. Using the COSY and HMBC spectra, the structure of **3** could be divided two parts, where part A was a (2*E*)-3-[4-(*β*-d-allopyranosyl)-3-methoxyphenyl unit and part B was a (4-hydroxy-3-methoxyphenyl)ethyl moiety (Figure 1). These parts were connected through an amide bridge based on the HMBC correlation of H-8′ (*δ*_H_ 3.51) with C-9 (*δ*_C_ 168.8) and the downfield-shift of the nitrogen-bearing methylene group at C-8′ (*δ*_C_ 42.4). From the evidence above, compound **3** was determined as a new compound with the (2*E*)-3-[4-(*β*-d-allopyranosyl)-3-methoxy-phenyl]-*N*-[2-(4–hydroxy-3-methoxyphenyl) ethyl]-2-propenamide structure.

Compound **4** was obtained as a white powder. The HR-ESI-MS spectra displayed the presence of an ion peak at *m*/*z* 765.2573 [M + Na]^+^, indicating a molecular formula of C_34_H_46_O_18_ (M = 742.26841). The ^1^H-NMR spectrum of **4** showed the resonance signal of methine protons at *δ*_H_ 3.14 (br s, H-1 and H-5), 4.79 (d, *J* = 7.5 Hz, H-2 and H-6), methylene protons [*δ*_H_ 3.96 (dd, *J* = 3.0, 9.0 Hz, H-4) and 4.32 (dd, *J* = 6.5, 9.0 Hz, H-8)], aromatic protons [*δ*_H_ 6.74 (s, H-2′, H-2″, H-6′, and H-6″)], and methoxy protons [*δ*_H_ 3.88 (s)]. The presence of a sugar moiety was recorded by the chemical signal of the anomeric proton [*δ*_H_ 5.17 (d, *J* = 7.5 Hz, H-1′″)] (Table 2). The ^13^C-NMR spectrum of **4** exhibited the resonance signals of 16 carbons including the aglycone and sugar unit. The carbon chemical shifts of the aglycone at *δ*_C_ 55.7 (C-1 and C-5), 87.2 (C-2 and C-6), 73.0 (C-4 and C-8), and aromatic carbons ranging from 104.8 to 154.4 suggested that **4** is a lignan skeleton with a tetrasubstituted aromatic ring. Comparing the ^1^H- and ^13^C-NMR spectroscopic data of **4** with those of a previously reported lignan named liriodendrin [16] revealed the similarity of their chemical shifts. However, the downfield-shift of the proton signal at H-1′″, H-3′″ to 5.17 (d, *J* = 7.5 Hz), 4.15 (t, *J* = 2.5 Hz), respectively, and upfield-shift of C-2′″, 3′″, 4′″ in the sugar unit (*δ_C_* 73.1, 72.2, and 68.7) indicated the presence of an allose moiety in the structure of **4** instead of glucose as in liriodendrin. These findings, together with the correlations of H-2′″, H-3′″, and H-4′″ and the absence of correlation between H-1′″ and H-3′″ in the ROESY spectrum indicated that the protons at C-1′″ were in a *β*-configuration and C-2′″, C-3′″, C-4′″ were the *α*-configuration. In addition, acid hydrolysis of **4** and comparison of the TLC, specific rotation, and properties of sugar unit with a sugar standard, in combination with the analysis of the coupling constant of H-1′″ (*J* = 7.5 Hz) confirmed the absolute the sugar to be a *β*-d-allopyranoside (Table 2).

In the HMBC spectrum, the correlations between H-1′″ (*δ*_H_ 5.17) and C-4′, C-4″ (*δ*_C_ 136.0), as well as H-2′, H-2″, H-6′, H-6″ (*δ*_H_ 6.74), with C-4′, C-4″ determined the allose moiety to be linked at C-4′ and C-4″. These evidences indicated that compound **4** was a new compound which was named syringaresinol di-*O*-*β*-d-allopyranoside.

Compound **7** was also obtained as a white amorphous powder with the molecular formula C_32_H_42_O_16_ (M = 682.24729), as determined by sodium adduct ion at *m*/*z* 705.2362 [M + Na]^+^ in the HR-ESI-MS. The structure of **7** showed similar lignan-skeleton chemical shifts with those of **4**, including carbon signals at *δ*_C_ 55.4, 87.0, 72.7, aromatic carbons from 111.6 to 150.8, and an allose moiety. However, the resonance signals of aromatic proton at *δ*_H_ 7.01 (br s, H-2′, 2″), 7.16 (d, *J* = 8.5 Hz, H-5′, 5″), 6.90 (br d, *J* = 8.0 Hz, H-6′, 6″), as well as the HMBC correlation of H-2′ (*δ*_H_ 7.01) and C-4′ (*δ*_C_ 147.6), C-6′ (*δ*_C_ 119.8); H-5′ (*δ*_H_ 7.16) and C-1′ (*δ*_C_ 137.1), C-3′ (*δ*_C_ 150.8); H-6′ (*δ*_H_ 6.90) and C-2′ (*δ*_C_ 111.6), C-4′ (*δ*_C_ 147.6) suggested the presence of an aromatic ring ABX spin system in the structure of **7** (Table 2). In addition, comparing the ^1^H- and ^13^C-NMR spectroscopic data of **7** with those of a previously reported lignan named (+)-pinoresinol di-*O*-*β*-d-glucopyranoside [16] revealed the similarity of their chemical shifts. However, the downfield-shift of the proton signal at H-1′″, H-3′″ to 5.26 (d, *J* = 7.5 Hz), 4.17 (brs), respectively, and upfield-shift of C-2′″, 3′″, 4′″ to the sugar unit (*δ_C_* 72.0, 72.8, and 68.6) indicated the presence of an allose moiety in the structure of **7** instead of glucose as is (+)-pinoresinol di-*O*-*β*-d-glucopyranoside. Thus, compound **7** was identified as a new compound which was named (+)-pinoresinol di-*O*-*β*-d-allopyranoside.

Compound **6** was obtained as a white amorphous powder. The HR-ESI-MS of **6** displayed a quasimolecular ion [M + Na]^+^ at *m*/*z* 805.4344, consistent with a molecular formula of C_41_H_66_O_14_ (M = 782.44526). The ^1^H-NMR spectrum of **6** (Table 3) indicated the presence of characteristic cyclopropane methylene signals [*δ*_H_ 0.43 and 0.68 (each 1H, d, *J* = 4.0 Hz, H-19)], one *sec*- and six *tert*-methyl groups [*δ*_H_ 1.17 (s, H-18), 0.93 (d, *J* = 6.5 Hz, H-21), 1.20 (s, H-26), 1.13 (s, H-27), 1.16 (s, H-28), 1.06 (s, H-29), and 0.91 (s, H-30)], and two anomeric protons [*δ*_H_ 4.30 (d, *J* = 8.0 Hz, H-1′) and 4.28 (d, *J* = 7.5 Hz, H-1″)]. In the ^13^C-NMR spectrum, the signals of a cyclopropane methylene carbon at *δ*_C_ 32.0 (C-19), four methine carbons bearing oxygen at *δ*_C_ 90.1 (C-3), 89.0 (C-15), 72.2 (C-23), and 90.1 (C-24), a ketone carbon at *δ*_C_ 112.4 (C-16), and two anomeric carbons at *δ*_C_ 107.3 (C-1′) and 105.2 (C-1″) were observed. The evidences above suggested that **6** was a 9,19-cimigenol-type triterpenoid diglycoside. After careful comparision, it was found that the NMR spectroscopic data of **6** and 3-*O*-*α*-l-arabinopyranosyl cimigenol 15-*O-β*-d-glucopyranoside were similar, except for the presence of a xylose unit in **6** instead of an arabinose unit [17]. The difference in the carbon chemical shift of C-2′, C-3′ and C-4′ of xylose unit [*δ*_C_ 75.6, 78.1, and 71.3, respectively] and those of arabinose unit [*δ*_C_ 73.1, 74.9, and 69.7] was recorded. Acid hydrolysis of **6** yielded an aglycone—cimigenol—and two sugar units. 

The coupling constant of the anomeric protons in the ^1^H-NMR spectrum of **6** (*J* = 7.5 and 8.0 Hz) indicated the *β*-configuration. In addition, the absolute configuration of the sugar moiety was confirmed as a d-xylopyranoside and d-glucopyranoside by comparing their TLC and specific rotations. The positions of the xylose and glucose units were determined based on HMBC analysis. In the HMBC spectrum, correlations were observed between the anomeric proton of the xylose unit [*δ*_H_ 4.30 (d, *J* = 8.0 Hz, H-1′)] and the methine signal [*δ*_C_ 90.1 (C-3)], the anomeric proton of glucose [*δ*_H_ 4.28 (d, *J* = 7.5 Hz, H-1″)] and the methine signal [*δ*_C_ 89.0 (C-15)]. Furthermore, the downfield-shift of C-3 and C-15 suggested that the xylose moiety was attached to C-3 and glucose was linked at C-15, respectively. Thus, compound **6** was elucidated to be 3-*O*-*β*-d-xylopyranosyl cimigenol-15-*O*-*β*-d-glucopyranoside.

The spectrums of compound **3**, **4**, **6**, **7** can be found in supplementary materials (Figure S1–S26). The structures of known compounds were identified as geniposide (**1**) [18], paeoniflorin (**2**) [19], 24-*O*-acetylhydroshengmanol 3-*O*-*β*-d-xylopyranosyl-15-*O-β*-d-glucopyranoside (**5**) [20], prim-*O*-glucosylcimifugin (**8**) [21], isoferulic acid (**9**) [22], acerinol (**10**) [23], 24-epi-acerinol (**11**) [24], 25-*O*-acetyl-cimigenol (**12**) [25], cimigenol (**13**) [26], 25-*O*-acetyl-7,8-didehydrocimigenol 3-*O-β*-d-xylopyranoside (**14**) [27], 25-*O*-acetylcimigenol 3-*O-β*-d-xylopyranoside (23*R*,24*S*) (**15**) [28], 24-epi-7,8-didehydrocimigenol 3-*O-β*-d-xylopyranoside (**16**) [24], (23*R*,24*S*) cimigenol 3-*O-β*-d-xylo-pyranoside (**17**) [29], based on comparison of their spectroscopic data with those in the literature. Compounds **1**, **2**, **10** were isolated from this plant for the first time.

### 2.2. Cytotoxicity Activity

#### 2.2.1. Cell Proliferation Activity

**Figure 2:**
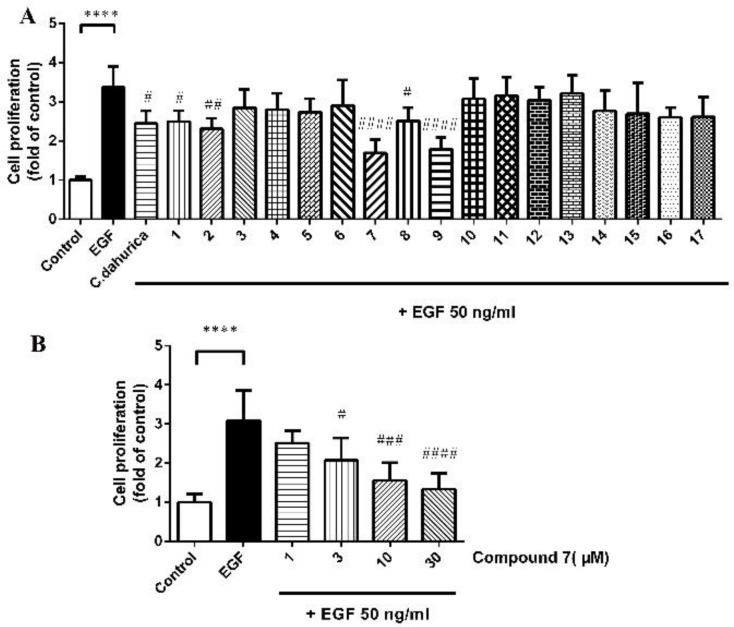
Proliferation of MCF-7 cells exposed to the extraction of *Cimicifuga dahurica* Maxim. at the concentration of 30 µg/mL or 10 µM of purified compounds (**1**–**17**) (**A**) and the increasing doses of compound **7** under simultaneous application of 50 mg/mL EGF (**B**). Means ± SD, n = 5 of three independent experiments: **** *p* < 0.0001 for the EGF group versus the control group; ^#^ *p* < 0.05, ^##^ *p* < 0.01, ^###^ *p* < 0.001, ^####^ *p* < 0.0001 versus the EGF group.

MCF-7 breast cancer cells were exposed to epidermal growth factor (EGF) which was known as a stimulator of proliferation (positive control). The anti-proliferative effects of the extract of *C. dahurica* and five purified compounds (compounds **1**, **2**, **7**, **8**, **9**) under co-incubation of MCF-7 cells with 50 ng/mL EGF had statistically significant differences compared to control vehicle (Figure 2A). Among these five purified compounds, compound **7** had the most potent anti-proliferative effect at the same concentration. In addition, the similar anti-proliferative effect of **7** was demonstrable under co-incubation of MCF-7 cells with 50 ng/mL EGF (Figure 2B).

To date, only two lignans have been found in the plants of *Cimicifuga* genus, actaealactone, a neolignan derived from *C. racemosa* [30] and (+)-isolariciresinol-3-*O-β*-d-glucopyranoside from *C. dahurica* [31]. Actaealactone exhibited a small stimulating effect on the growth of MCF-7 breast cancer cells about 1.24-fold (14 µM) compared to untreated cells, while compound **7** exhibited the significantly anti-proliferative effect on MCF-7 cells even at the lower concentration (3 µM), showing more potential anti-proliferative effect.

#### 2.2.2. Cell Cycle Distribution and Western Blot Analyses

**Figure 3:**
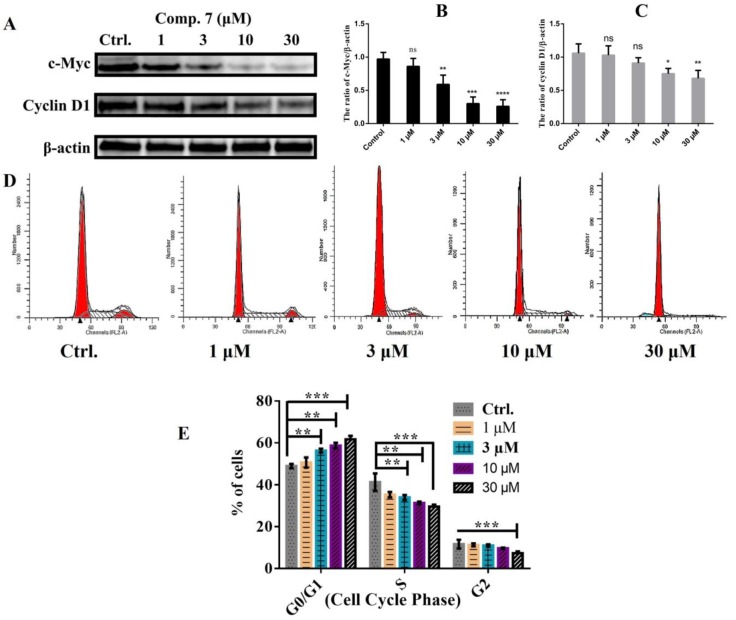
The effect of compound **7** on breast cancer MCF-7 cell cycle. The cells were plated overnight and then treated with compound **7** at the indicated concentrations for 48 h. (**A**) The western blot was performed using antibody against cyclin D1 and c-Myc. *β*-actin was used as internal control for western blot analysis. (**B**,**C**) The results of the western blotting in cyclin D1 and c-Myc were evaluated in three independent experiments and normalized with *β*-actin. (**D**,**E**) After treating the cells with varying concentrations of compound **7**, the cells were collected and fixed with 75% ethanol and then re-incubated in PBS with RNase and then stained with propidium iodide and then subjected to flow cytometry for DNA content was determination percentage at various cell cycle phases. * *p* < 0.1, ** *p* < 0.01, *** *p* < 0.001, **** *p* < 0.0001 versus the control group, *ns*: no significant difference.

For exploration of the anti-proliferation mechanism of compound **7**, western blotting was performed to determine the effect of compound **7** to the levels of cyclin D1 and c-Myc. The cyclin D1 proto-oncogene exercises powerful control over the mechanisms that regulate the mitotic cell cycle, and overexpression of cyclin D1 is common in human cancers [32]. C-Myc encodes for a transcription factor that is a key regulator of a wide variety of cellular processes. In most non-malignant cells, Myc levels are low, its constitutive expression is linked to the pathogenesis of many human cancers [33]. As shown in Figure 3A–C, the downregulation of cyclin D1 protein level and c-Myc level were observed in the cells treated with compound **7**. After observing the expression variation of Cyclin D1 and c-Myc, we further assessed the cell cycle phases to explore the variations. Our results showed that compound **7** caused cell cycle transition arrest in the G0/G1 phase that was markedly increased in dose dependent manner (Figure 3D,E) in breast cancer MCF-7 cells. The increased arrest of cell cycle at G0/G1 phase was contrastingly reduced in subsequent S and G2/M phase indicting that the cell cycle was arrested within G0/G1 to S phase while a very small proportion was arrested in G2/M phase. These results seconded our earlier results of Cyclin D1 and c-Myc (Figure 3A–C).

**Figure 4:**
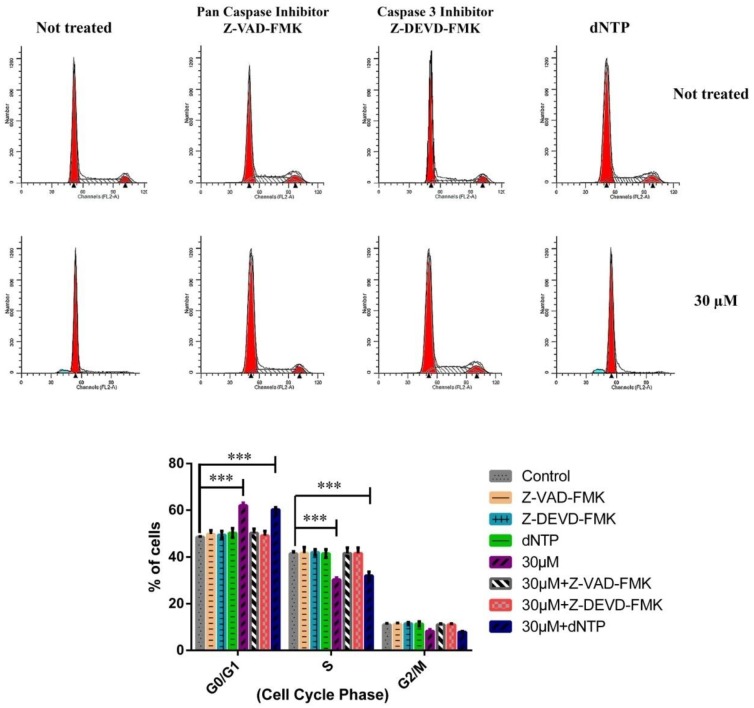
The effects of a caspase 3 inhibitor, a pan caspase inhibitor and nucleoside on compound **7**-induced cell cycle arrest in MCF-7 cells. *** *p* < 0.001 versus the control group.

We examined the effect of the caspase inhibitors and the nucleoside addition on compound 7-induced cell cycle arrest in MCF-7 cells by adding a caspase 3 inhibitor (50 μM, Z-DEVD-FMK), a pan caspase inhibitor (50 μM, Z-VAD-FMK) or a nucleoside mixed solution of dATP, dCTP, dGTP and dTTP (125 µM of each) (dNTP Mixture) to the cultured media with compound **7** (30 μM). As shown in Figure 4, the cell cycle arrest at G0/G1 phase was reversed by a pan caspase inhibitor or a caspase 3 inhibitor but not the nucleoside addition. These results demonstrated that pan caspase and caspase 3 maybe had major roles in the mechanism of cell cycle arrest effects of compound **7** on MCF-7 cells.

#### 2.2.3. Induction of Apoptosis by Compound **7** in MCF-7 Cells

**Figure 5:**
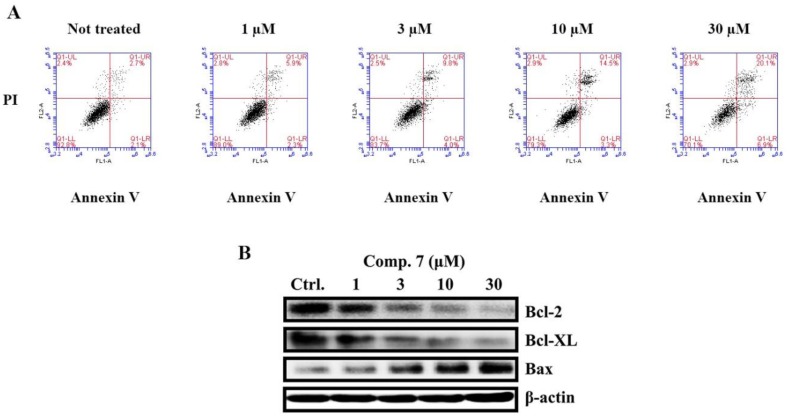
Apoptosis induced by compound **7** in MCF-7 cells. (**A**) Apoptosis quantification, using Annexin V/PI double staining assay after treatment with compound **7** for 48 h. MCF-7 cells were harvested and stained with PI and Annexin V-FITC in darkness for 15 min, followed by flow cytometry analysis. Lower right: Annexin V+/PI− (early apoptosis); upper right: Annexin V+/PI+ (late apoptosis and necrosis). (**B**) The expression of Bcl-2, Bcl-XL and Bax was assessed through western blotting analyses of MCF-7 (treated for 48 h with varying concentrations of the compound), *β*-actin was used as internal control.

After MCF-7 cells were treated with compound **7** (0, 1, 3, 10, 30 μM) for 48 h, cells were stained with annexin V/PI and examined using a BD flow cytometer. Early and late apoptosis and necrotic cells were discriminated. The corresponding quantities of total cell apoptosis and early apoptosis were 4.8% and 2.1%; 8.2% and 2.3%; 13.8% and 4.0%; 17.8% and 3.3%; 27.0% and 6.9%; respectively. The data demonstrated that apoptotic cells were found to be increased in a dose-dependent manner (Figure 5A). We further assessed the expression variations of Bcl-2 family proteins including Bcl-2, Bcl-XL (anti-apoptotic proteins) and Bax (pro-apoptotic protein). Our results showed that after 48 h treatment of compound **7**, the expression of anti-apoptotic proteins (Bcl-2, Bcl-XL) were decreased in dose dependent manner alternatively the expression of pro-apoptotic protein (Bax) was increased in breast cancer MCF-7 cells (Figure 5B).

**Figure 6:**
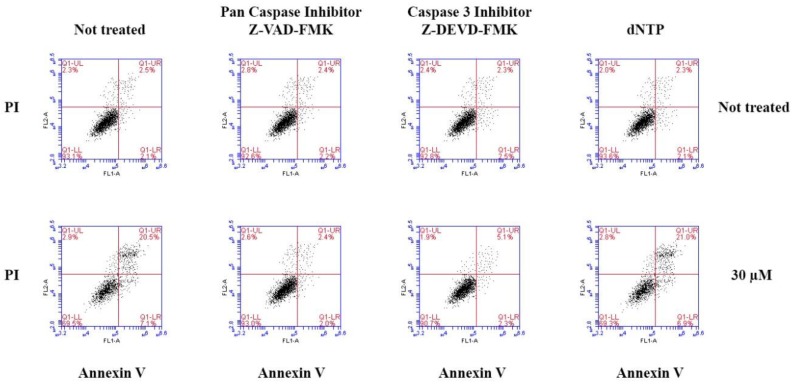
The effects of a caspase 3 inhibitor, a pan caspase and nucleoside on compound **7**-induced apoptosis in MCF-7 cells.

To determine whether cell apoptosis was dependent upon caspase activation and the influence of nucleoside in the cultured media on the cells apoptosis produced by compound **7**, we examined the effects of the caspase inhibitors and the nucleoside addition on compound **7**-induced apoptosis in MCF-7 cells using Annexin V-FITC/PI kit. As shown in Figure 6, apoptosis was reduced by a pan caspase inhibitor or a caspase 3 inhibitor but not the nucleoside addition. These results demonstrate that compound **7** induces apoptosis in MCF-7 cells by activating a pan caspase and caspase 3-dependent apoptotic pathway.

Collectively in our present study we conclusively found four new compounds including one new phenolic amide glycoside **3**, two new lignan glycosides **4** and **7**, and one new 9,19-cycloartane triterpenoid glycoside **6**. In addition to these four novel compounds we also isolated and purified thirteen other previously known compounds **1**, **2**, **5**, and **8**–**17** from the ethanol extract of *C. dahurica*. In continuation of our studies searching for novel anti-cancer agents from natural products, we found that the ethanol extract from the rhizomes of *C. dahurica* exhibited considerable inhibitory effects on the proliferation of MCF-7 cells through the BrdU-proliferation test. Moreover we found that compound **7** had significant anti-proliferatory and apoptotic effects against breast cancer MCF-7 cells. In additions, we also found that compound **7** had significant cell cycle arresting (at G0/G1-S) effects following graded dose response effectiveness. The apoptotic effects and cell cycle arresting (at G0/G1-S) effects of compound **7** maybe through caspase 3 and pan caspase-dependent pathways. Although compounds **7** and **4** were two chemically similar lignans with the same sugar moieties, the higher anti-proliferative activity in MCF-7 cells of **7** compared to **4** suggested that two methoxy units in the benzene ring (rather than four methoxy units) may be associated with increased biological potency.

## 3. Materials and Methods

### 3.1. General Experimental Proceduces

The ^1^H and ^13^C, DEPT, HSQC, HMBC, ROESY, and COSY NMR spectra were recorded on a Avance 500 MHz instrument (Bruker, Switzerland). The high-resolution electrospray ionization mass spectra (HR-ESI-MS) were obtained from an Agilent 6530 Accurate-Mass Q-TOF LC/MS system (Agilent Technologies Co., Ltd., Waldbronn, Germany). Semi-preparative HPLC was performed on a Agilent 1100 series system,)using an ODS column (Zorbax SB-C_18_, 9.4 *×* 250 mm, 5 µm, Agilent Technologies Inc., Santa Clara, CA, USA). Column chromatography was performed on silica gel (200–300 mesh and 300–400 mesh, Qingdao Haiyang Chemical Co., Ltd., Qingdao, China), Sephadex^TM^ LH-20 (GE Healthcare Bio-Sciences AB, Uppsala, Sweden), and octadecyl silica (ODS, 50 μm, YMC Co., Ltd., Tokyo, Japan). Thin-layer chromatography was performed on a precoated GF_254_ plate (Qingdao Haiyang Chemical Co., Ltd., Qingdao, China), RP-18 F_254_S plates (Merck, Kenilworth, NJ, USA) and compounds were visualized by spraying with aqueous 10% H_2_SO_4_ and then heating for 3–5 min.

### 3.2. Plant Material

The rizhomes of *Cimicifuga dahurica* were collected from Panshi City, Jilin Province, China in November 2015. The plant materials were authenticated by Prof. Li Hui-Jun of the Department of Pharmacognosy, China Pharmaceutical University. A voucher specimen (No. XASM201511) was deposited in the State Key Laboratory of Natural Medicines, China Pharmaceutical University, Nanjing, China.

### 3.3. Extraction and Isolation

The air-dried rizhomes (45 kg) of *Cimicifuga dahurica* were successively extracted three times with 70% EtOH (3 × 400 L) for 2 h under reflux. The combined extracts were concentrated under reduced pressure to afford a dark brown residue extract (3.0 kg), which was suspended in H_2_O and partitioned successively with petroleum ether (3 × 3 L), EtOAc (3 × 3 L), and *n*-BuOH (3 × 3 L) to afford petroleum ether (70 g, CP), EtOAc (1200 g, CE), *n*-BuOH (368 g, CB) fractions, and a H_2_O layer (W).

The *n*-butanol fraction (368 g) was fractionated on a silica gel column chromatography (CC) eluting with gradient solvent systems of CH_2_Cl_2_/MeOH (0–100% MeOH, step-wise) to obtain five fractions including: CB-1 (35 g), CB-2 (78 g), CB-3 (42 g), CB-4 (12 g), and CB-5 (175 g). Fraction CB-4 (12 g) was isolated by CC over silica gel, eluting with solvent systems of CH_2_Cl_2_/MeOH (10/1, *v*/*v*) to obtain three subfractions (CB-4.1 through CB-4.3). Compounds **4** (10 mg), **7** (11 mg) and **8** (20 mg) were afforded from fraction CB-4.1 (7.5 g) by silica gel CC eluting with CH_2_Cl_2_/MeOH (1/1, *v*/*v*), and purified by RP-C_18_ silica gel using acetone/H_2_O (3/2, *v*/*v*) as eluent. Fraction CB-4.2 (2.5 g) was fractioned on RP-C_18_ silica gel eluting with acetone/H_2_O (2.5/1, *v*/*v*), and further isolated by silica gel CC using solvent system CH_2_Cl_2_/acetone/MeOH (5/1/0.1, *v*/*v*/*v*) to give compounds **5** (8 mg), and **6** (11 mg). Fraction CB-4.3 (1.1 g) was subjected by Sephadex^TM^ LH-20 CC eluting with MeOH/H_2_O (1/1, *v*/*v*) and further isolated by RP-C_18_ silica gel using solvent system MeOH/H_2_O (2/1, *v*/*v*) to give compounds **1** (42 mg), **2** (20 mg), and **3** (24 mg).

A portion of the ethylacetat fraction (700 g) was applied to a silica gel column chromatography (CC) using a stepwise gradient elution of CH_2_Cl_2_/MeOH (0–100% MeOH) to afford seven fractions including CE-1 (32 g), CE-2 (50 g), CE-3 (55 g), CE-4 (98 g), CE-5 (250 g), CE-6 (100 g) and CE-7 (55 g). Fraction CE-4 (98 g) was chromatographed on a silica gel column by eluting with petroleum ether/aceton (100/0 → 4/1, *v*/*v*) to give three subfractions (CE-4.1 through CE-4.3). Fraction CE-4.1 (10 g) give compound **11** (80 mg) on crystallization from EtOAc. Repeated silica gel column chromatography of fraction CE-4.2 (30 g) with petroleum ether/EtOAc (20/1 → 1/1) and recrystallization to yield compounds **10** (70 mg), **12** (100 mg) and **13** (200 mg). Fraction CE-5 (250 g) was subjected to a silica gel column chromatography with a gradient mixture of petroleum ether/aceton (5/1 → 1/1, *v*/*v*) as eluent to give 5 subfractions (CE-5.1 to CE-5.5). Fraction CE-5.2 (80 g) was crystallized from MeOH to give an addition crop of compound **9** (10 g). Fraction CE-5.4 (53 g) was further separated into three subfractions (CE-5.4.1 to CE-5.4.3) on a silica gel column (CH_2_Cl_2_/acetone, 4/1 → 3/2, *v*/*v*). A part of fraction CE-5.4.1 (4.0 g) was purified by RP-C_18_ silica gel using MeOH/ H_2_O (4/1, *v*/*v*) as eluent, followed by purification using semi-preparative HPLC eluted with CH_3_CN/H_2_O (60:40), to yield compounds **14** (30 mg) and **15** (60 mg). A portion of fraction CE-5.4.2 (5 g) was applied on RP-C_18_ silica gel using MeOH/ H_2_O (3/1, *v*/*v*) as eluent to give compound **16** (50 mg). Repeated silica gel column chromatography of fraction CE-5.5 (40 g) using CH_2_Cl_2_/acetone (2/1, *v*/*v*) and recrystallization furnished compound **17** (1.5 g).

### 3.4. Acid hydrolysis and Sugar Identification

The new compounds **3**, **4**, **6** and **7** (2 mg of each) were dissolved in MeOH (2 mL) and refluxed with 1.0 N HCl (2 mL) for 3 h and following by extracting with ethyl acetate. The water layer was then neutralized by Ag_2_CO_3_, and filtered to give a monosaccharide. Each monosaccharide of those compounds had an *Rf* (n-BuOH–AcOH–H_2_O, 4:1:1) and specific rotation [*α*]d + 52.7° (c 0.10, H_2_O); [*α*]d + 24.3° (c 0.10, H_2_O); [*α*]d + 14.4° (c 0.05, H_2_O) corresponding to those of d-glucose, d-xylose and d-allose (Sigma-Aldrich, St. Louis, MO, USA), respectively.

### 3.5. Biological Evaluations

#### 3.5.1. Cell Culture

MCF-7 cells were purchased from the American Type Culture Collection (ATCC, Rockville, MD, USA). Cells were cultured and growth in Dulbecco’s modified Eagle’s medium (DMEM) (Gibco, Grand Island, NY, USA) medium containing 10% fetal bovine serum (FBS, Gibco, Grand Island, NY, USA), 100 units/mL penicillin, and 100 μg/mL streptomycin (Gibco, Grand Island, NY, USA) at 37°C in 5% CO_2_—95% air.

#### 3.5.2. Anti-Proliferative Activity

After treatment with *C. dahurica* extract at the concentration of 30 mg/mL and 10 µM of purified compounds (**1**–**17**) under co-incubation with 50 ng/mL EGF, MCF-7 cells were incubated with BrdU labelling solution (10 μM) for 2 h and fixed with fixation solution for 30 min at room temperature. 100 μL anti-BrdU peroxidase-labelled antibody was added for 90 min and washed three times. 100 μL/well of substrate solution for colorimetric quantification was added at room temperature for 5–30 min until colour development was sufficient for photometric detection. The absorbance was measured at 405 nm using a Synergy 2 multi-detection microplate reader (Bioteck, Winooski, VT, USA).

#### 3.5.3. Cell Cycle Distribution Analyses

We cultured breast cancer MCF-7 cells in small dishes and after 24 h of incubation the cells were treated with varying concentrations (1, 3, 10 and 30 μM) of compound **7**. The cells were then further incubated for 48 h and then collected. The harvested cells were centrifuged at 1000 rpm for 5 min, washed thoroughly with ice cold PBS and were then subjected to fixation in 75% ethanol over night at 4 °C. After fixation the ethanol was removed and cells (about 1 × 10^6^) were re-suspended in PBS having RNase (25 µg/mL), then the cells were further incubated for another half hour in propidium iodide (50 µg/mL) at 37 °C and subjected to flow cytometer analyses. Cell cycle distribution was analyzed by ModFitLT (Verity, Software House, Topsham, ME, USA).

To determine whether cell cycle distribution is dependent upon caspase activation and the influence of the nucleoside addition to apoptotic activity of compound **7**, a caspase 3 inhibitor (50 μM, Z-DEVD-FMK, Medchem Express, Monmouth Junction, NJ, USA), a pan caspase inhibitor (50 μM, Z-VAD-FMK, Beyotime Biotechnology, Shanghai, China) or a nucleoside mixed solution of dATP, dCTP, dGTP and dTTP (125 µM of each) (dNTP Mixture, Beyotime Biotechnology) were added to the media with compound **7** (30 µM) to incubation for 48 h and followed by flow cytometer analyses as described above.

#### 3.5.4. Annexin V/PI Analyses

The Annexin V-FITC/PI apoptosis detection kit (Keygen Biotechnology, Nanjing, China) was used to detect the effects of compound **7**. MCF-7 cells (3 × 10^5^ cells/well) were seeded in 6-well plates and cultured for 24 h. After treatment of cells with compound **7** (0, 3, 10, 30 μM) for 48 h, adherent and floating cells were collected and washed with ice cold PBS, then cells were centrifuged. The pellet was suspended with binding buffer (500 μL), stained with Annexin V-FITC (5 μL) and PI (5 μL), and then incubated for 15 min at room temperature in the dark. After incubation, cells were analyzed with a BD Accuri C6 Flow Cytometer (BD Biosciences, San Jose, CA, USA) to determine the percentage of apoptotic cells.

To determine whether cell apoptosis is dependent upon caspase activation and the influence of the nucleoside addition on apoptotic activity of compound **7**, a caspase 3 inhibitor (50 μM Z-DEVD-FMK, Medchem Express, Monmouth Junction, NJ, USA), a pan caspase inhibitor (50 μM Z-VAD-FMK, Beyotime Biotechnology, Shanghai, China) or a nucleoside mixed solution of dATP, dCTP, dGTP and dTTP (125 µM of each) (dNTP Mixture, Beyotime Biotechnology) were added to the media with compound 7 (30 µM) and incubated for 48 h.

#### 3.5.5. Western Blotting

MCF-7 cells were seeded in 6-well plates and treated with compound **7***.* Cells were collected and washed with cold phosphate-buffered saline (PBS). The collected cells were lysed on ice for 30 min in 100 μL lysis buffer (120 mM NaCl, 40 mM Tris (pH 8), 1% Nonidet P-40 and protease inhibitors) and centrifuged at 12,000 rpm for 30 min. BCA protein assay kit (Beyotime, Shanghai, China) was used to measure protein concentrations as described in a previous report [34]. Whole-cell extracts were subjected to electrophoresis on polyacrylamide gels and then transferred onto nitrocellulose membranes. The membranes were blocked with 5% skim milk in Tris-buffered saline with Tween-20 (TBST). Then, anti-cyclin D1, anti-Bcl-2, anti-Bcl-XL, anti-Bax antibodies (Cell Signaling Technology, Danvers, MA, USA), anti c-Myc antibody (Abcam, Cambridge, MA, USA) or anti-actin antibody (Santa Cruz Biotechnology, Dallas, TX, USA) was bound overnight at 4 °C. After washing with TBST, HRP conjugated secondary antibody (Keygen, Nanjing, China) was bound for 1 h at room temperature. Finally, protein bands were detected using a Tanon 5200 Multi Chemiluminescent Imaging System (Tanon, Shanghai, China). The ratio of cyclin D1/*β*-actin and c-Myc/*β*-actin were analyze using NIH Image J.

#### 3.5.6. Statistics

Values are presented as mean ± SD. *p*-values were calculated by one-way analysis of variance followed by Bonferroni post-hoc testing using GraphPad Prism 6.01 (GraphPad Software Inc., San Diego, CA, USA). *p* < 0.05 was considered statistically significant. * *p* < 0.05; ** *p* < 0.01; *** *p* < 0.001; **** *p* < 0.0001.

## Figures and Tables

**Table 1:** ^1^H- and ^13^C-NMR data of **3** in CD_3_OD.

Position	*δ*_C_^a^	*δ*_H_^b^ (Mult., *J* in Hz)	Position	*δ*_C_^a^	*δ*_H_^b^ (Mult., *J* in Hz)
1	130.9	-	5′	116.2	6.75 (d, 8.0)
2	112.4	7.17 *	6′	122.2	6.68 (dd, 1.5, 8.0)
3	150.9	-	7′	36.1	2.79 (t, 7.5)
4	149.7	-	8′	42.4	3.51 (t, 7.5)
5	117.3	7.17 *	3-OCH_3_	56.7	3.88 (s)
6	122.7	7.12 (br s)	3′-OCH_3_	56.4	3.84 (s)
7	141.4	7.47 (d, 15.5)	1″	100.2	5.32 (d, 8.0)
8	120.4	6.50 (d, 15.5)	2″	72.0	3.70 *
9	168.8	-	3″	72.9	4.18 (t, 3.0)
1′	132.0	-	4″	68.6	3.63 (dd, 3.0, 10.0)
2′	113.5	6.83 (d, 1.5)	5″	75.9	3.87 (m)
3′	148.9	-	6″	62.8	3.71 *
4′	146.0	-			3.88 (dd, 6.0, 12.0)

^a^ 125 MHz, ^b^ 500 MHz. * Overlapped signals. Assignments were made using the HMQC and HMBC spectra.

**Table 2:** ^1^H- and ^13^C-NMR Data of **4** and **7** in CD_3_OD.

Position	4		7	
	*δ*_C_^a^	*δ*_H_^b^ (Mult., *J* in Hz)	*δ*_C_^a^	*δ*_H_^b^ (Mult., *J* in Hz)
1, 5	55.7	3.14 (br s)	55.4	3.09 (br s)
2, 6	87.2	4.79 (d, 7.5)	87.0	4.74 (br s)
4, 8	73.0	3.96 (dd, 3.0, 9.0)	72.7	3.86 *
		4.32 (dd, 6.5, 9.0)		4.22 (d, 6.0)
1′, 1″	139.4	-	137.1	-
2′, 2″	104.8	6.74 (s)	111.6	7.01 (br s)
3′, 3″	154.4	-	150.8	-
4′, 4″	136.0	-	147.6	-
5′, 5″	154.4	-	117.7	7.16 (d, 8.5)
6′, 6″	104.8	6.74 (s)	119.8	6.90 (br d, 8.0)
OCH_3_	57.1	3.88 (s)	56.8	3.88 (s)
Allo-1′″, 1″″	103.9	5.17 (d, 7.5)	100.6	5.26 (d, 7.5)
2′″, 2″″	73.1	3.62 *	72.0	3.63 *
3′″, 3″″	72.2	4.15 (t, 2.5)	72.8	4.17 (br s)
4′″, 4″″	68.7	3.65 *	68.6	3.60 *
5′″, 5″″	76.3	3.64 (m)	75.7	3.82 m
6′″, 6″″	63.0	3.78 (dd, 6.0, 12.0)	62.8	3.65 *
		3.68 *		3.85 (dd, 6.0, 12.0)

^a^ 125 MHz, ^b^ 500 MHz. * Overlapped signals. Assignments were made using the HMQC and HMBC.

**Table 3:** ^1^H- and ^13^C-NMR Data of **6** in CD_3_OD.

Position	*δ*_C_^a^	*δ*_H_^b^ (Mult., *J* in Hz)	Position	*δ*_C_^a^	*δ*_H_^b^ (Mult., *J* in Hz)
1	33.5	1.29 (m)/1.57 (m)	24	90.1	3.45 (s)
2	30.5	1.95 (m)/1.70 (m)	25	71.9	-
3	90.1	3.21 (dd, 4.5, 11.5)	26	27.3	1.20 (s)
4	42.0	-	27	25.3	1.13 (s)
5	49.8	1.72 (m)	28	12.6	1.16 (s)
6	22.1	0.81 (m)/1.62 (m)	29	26.0	1.06 (s)
7	27.3	1.96 (m)/1.21 (m)	30	15.5	0.91 (s)
8	48.8	1.36 (m)	*3-Xylose*		
9	21.2	-	1′	107.3	4.30 (d, 8.0)
10	27.7	-	2′	75.6	3.28 (dd, 7.5, 8.0)
11	27.0	1.22 (m)/2.15 (m)	3′	78.1	3.34 (dd, 8.0, 8.0)
12	34.8	1.66 (m)/1.74 (m)	4′	71.3	3.58 *
13	42.5	-	5′	66.7	3.19 (dd, 10.0, 11.0)
14	48.3	-			3.84 (dd, 5.0, 11.0)
15	89.0	3.97 (s)	*15-Glucose*		
16	112.4	-	1″	105.2	4.28 (d, 7.5)
17	60.3	1.46 (d, 11.0)	2″	75.4	3.21 (dd, 8.0, 9.0)
18	19.9	1.17 (s)	3″	78.0	3.37 *
19	32.0	0.43 (d, 4.0)/0.68 (d, 4.0)	4″	72.4	3.26 *
20	25.0	1.68 (m)	5″	77.5	3.31 (m)
21	20.2	0.93 (d, 6.5)	6″	63.0	3.62 (dd, 5.5, 11.5)
22	38.8	1.03 (m)/2.35 (ddd, 2.5, 6.0, 13.0)			3.79 (dd, 2.5, 11.5)
23	72.2	4.42 (br d, 9.0)			

^a^ 125 MHz, ^b^ 500 MHz. * Overlapped signals. Assignments were made using the HMQC and HMBC.

## Supplementary Materials

Supplementary materials are available on line.

## References

[1] Guo Y.Q., Yin T., Wang X.M., Zhang F., Pan G.X., Lv H., Wang X.R., Owoicho Orgah J., Zhu Y., Wu H.H. (2017). Traditional uses, phytochemistry, pharmacology and toxicology of the genus *Cimicifuga*: A review. J. Ethnopharmacol..

[2] The State Pharmacopoeia Commission of P.R.China (2015). Pharmacopoeia of Chinese People’s Republic.

[3] Li J.X., Yu Z.Y. (2006). Cimicifugae rhizoma: From origins, bioactive constituents to clinical outcomes. Curr. Med. Chem..

[4] Hao D.C., Gu X.J., Xiao P.G., Liang Z.G., Xu L.J., Peng Y. (2013). Recent advance in chemical and biological studies on cimicifugeae pharmaceutical resources. Chin. Herb. Med..

[5] Borrelli F., Ernst E. (2008). Black cohosh (*Cimicifuga racemosa*) for menopausal symptoms: A systematic review of its efficacy. Pharmacol. Res..

[6] Tian Z., Si J., Chang Q., Zhou L., Chen S., Xiao P., Wu E. (2007). Antitumor activity and mechanisms of action of total glycosides from aerial part of *Cimicifuga dahurica* targeted against hepatoma. BMC Cancer.

[7] Schmid D., Woehs F., Svoboda M., Thalhammer T., Chiba P., Moeslinger T. (2009). Aqueous extracts of *Cimicifuga racemosa* and phenolcarboxylic constituents inhibit production of proinflammatory cytokines in LPS-stimulated human whole blood. Can. J. Physiol. Pharmacol..

[8] Seidlova-Wuttke D., Stecher G., Kammann M., Haunschild J., Eder N., Stahnke V., Wessels J., Wuttke W. (2012). Osteoprotective effects of *Cimicifuga racemosa* and its triterpene-saponins are responsible for reduction of bone marrow fat. Phytomedicine.

[9] Borrelli F., Ernst E. (2017). Black cohosh (*Cimicifuga racemosa*): A systematic review of adverse events. Am. J. Obstet. Gynecol..

[10] Nian Y., Wang H.Y., Zhou L., Su J., Li Y., Qiu M.H. (2013). Cytotoxic cycloartane triterpenes of the traditional Chinese medicine “shengma” (*Cimicifuga dahurica*). Planta Med..

[11] Chen J.Y., Li P.L., Tang X.L., Wang S.J., Jiang Y.T., Shen L., Xu B.M., Shao Y.L., Li G.Q. (2014). Cycloartane triterpenoids and their glycosides from the rhizomes of *Cimicifuga foetida*. J. Nat. Prod..

[12] Watanabe K., Mimaki Y., Sakagami H., Sashida Y. (2002). Cycloartane glycosides from the rhizomes of *Cimicifuga racemosa* and their cytotoxic activities. Chem. Pharm. Bull..

[13] Yue G.G., Xie S., Lee J.K., Kwok H.F., Gao S., Nian Y., Wu X.X., Wong C.K., Qiu M.H., Lau C.B. (2016). New potential beneficial effects of actein, a triterpene glycoside isolated from *Cimicifuga* species, in breast cancer treatment. Sci. Rep..

[14] Jarry H., Stromeier S., Wuttke W., Nahrstedt A. (2007). Petasiphenone, a phenol isolated from *Cimicifuga racemosa*, in vitro inhibits proliferation of the human prostate cancer cell line LNCaP. Planta Med..

[15] Yim S.H., Kim H.J., Park S.H., Kim J., Williams D.R., Jung D.W., Lee I.S. (2012). Cytotoxic caffeic acid derivatives from the rhizomes of *Cimicifuga heracleifolia*. Arch. Pharm. Res..

[16] Deyama T. (1983). The constituents of *Eucommia ulmoides* Oliv. I. Isolation of (+)-medioresinol di-*O-β*-d-glucopyranoside. Chem. Pharm. Bull..

[17] Zhang Q.W., Ye W.C., Hsiao W.W., Zhao S.X., Che C.T. (2001). Cycloartane glycosides from *Cimicifuga dahurica*. Chem. Pharm. Bull..

[18] Lelono R.A., Tachibana S., Itoh K. (2009). Isolation of antifungal compounds from *Gasdenia jasminoides*. Pak. J. Biol. Sci..

[19] Yen P.H., Kiem P.V., Nhiem N.X., Tung N.H., Quang T.H., Minh C.V., Kim J.W., Choi E.M., Kim Y.H. (2007). A new monoterpene glycoside from the roots of *Paeonia lactiflora* increases the differentiation of osteoblastic MC3T3-E1 cells. Arch. Pharm. Res..

[20] Sakurai N., Koeda M., Inoue T., Nagai M. (1994). Studies on the Chinese crude drug “Shoma.” VIII. Two new triterpenol bisdesmosides, 3-arabinosyl-24-*O*-acetylhydroshengmanol 15-glucoside and 3-xylosyl-24-*O*-acetylhydroshengmanol 15-glucoside, from *Cimicifuga dahurica*. Chem. Pharm. Bull..

[21] Liu R.M., Wu S.J., Sun A.L. (2008). Separation and purification of four chromones from radix saposhnikoviae by high-speed counter-current chromatography. Phytochem. Anal..

[22] Li A.F., Xuan H.Z., Sun A.L., Liu R.M., Cui J.C. (2016). Preparative separation of polyphenols from water-soluble fraction of Chinese propolis using macroporous absorptive resin coupled with preparative high performance liquid chromatography. J. Chromatogr. B.

[23] Kusano A., Shibano M., Kusano G., Miyase T. (1996). Studies on the constituents of *Cimicifuga* species. XIX. Eight new glycosides from *Cimicifuga simplex* Wormsk. Chem. Pharm. Bull..

[24] Li J.X., Kadota S., Hattori M., Yoshimachi S., Shiro M., Oogami N., Mizuno H., Namba T. (1993). Constituents of Cimicifugae rhizoma. I. Isolation and characterization of ten new cycloartenol triterpenes from *Cimicifuga heracleifolia* Komarov. Chem. Pharm. Bull..

[25] Kusano A., Shibano M., Kitagawa S., Kusano G., Nozoe S., Fushiya S. (1994). Studies on the constituents of *Cimicifuga* species. XV. Two new diglycosides from the aerial parts of *Cimicifuga simplex* Wormsk. Chem. Pharm. Bull..

[26] Cuong T.D., Lim C.J., Kim S.W., Park J.E., Hung T.M., Min B.S. (2011). Isolation of compounds from Cimicifugae rizhoma and their cytototoxic activity. Nat. Prod. Sci..

[27] Kusano A., Takahira M., Shibano M., Miyase T., Kusano G. (1999). Studies on the constituents of *Cimicifuga* Species. XXVI. Twelve new cyclolanostanol glycosides from the underground parts of *Cimicifuga simplex* Wormsk. Chem. Pharm. Bull..

[28] Kadota S., Li J.X., Tanaka K., Namba T. (1995). Constituents of Cimicifugae rizhoma II. Isolation and structures of new cycloartenol triterpenoids and related compounds from *Cimicifuga foetida* L.. Tetrahedron.

[29] Zhang Q.W., Ye W.C., Che C.T., Zhao S.X. (1999). A new cycloartane saponin from *Cimicifuga acerina*. J. Asian Nat. Prod. Res..

[30] Nuntanakorn P., Jiang B., Einbond L.S., Yang H., Kronenberg F., Weinstein I.B., Kennelly E.J. (2006). Polyphenolic constituents of *Actaea racemosa*. J. Nat. Prod..

[31] Zhang F., Han L.F., Pan G.X., Peng S., Andre N. (2013). A new phenolic amide glycoside from *Cimicifuga dahurica*. Acta Pharm. Sin..

[32] Knudsen K.E., Diehl J.A., Haiman C.A., Knudsen E.S. (2006). Cyclin D1: Polymorphism, aberrant splicing and cancer risk. Oncogene.

[33] Poole C.J., Zheng W., Lodh A., Yevtodiyenko A., Liefwalker D., Li H., Felsher D.W., van Riggelen J. (2017). DNMT3B overexpression contributes to aberrant DNA methylation and MYC-driven tumor maintenance in T-ALL and Burkitt’s lymphoma. Oncotarget.

[34] Cao Y., Huang H.T., Wang Z.X., Zhang G.B. (2017). The inflammatory CXC chemokines, GROα^high^, IP-10^low^, and MIG^low^, in tumor microenvironment can be used as new indicators for non-small cell lung cancer progression. Immunol. Investig..

