# Supplementary material for: Chemical Constituents from Cimicifuga dahurica and Their Anti-Proliferative Effects on MCF-7 Breast Cancer Cells
Source: Molecules. 2018 May 4;23(5):1083. doi: 10.3390/molecules23051083 (PMC6102574; doi:10.3390/molecules23051083)
Supplement: Supplementary file 1 [file molecules-23-01083-s001.zip › Supplementary Materials_liping/Figure S1. 1H spectrum of compound 3.pdf]

CB3-MeOD-1H

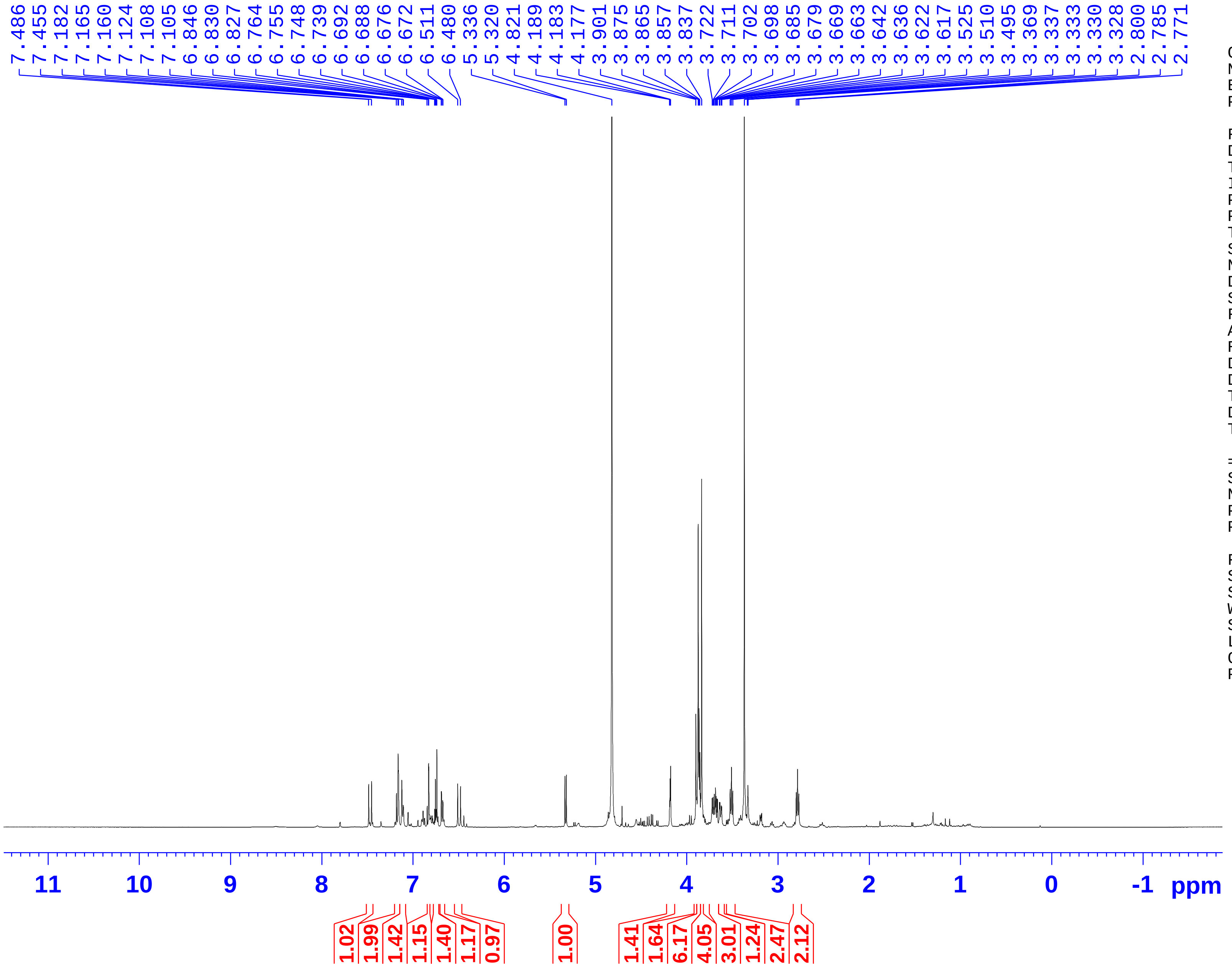


```
Current Data Parameters
NAME              11D_CB3
EXPNO                   1
PROCNO                  1

F2 - Acquisition Parameters
Date_            20170601
Time                16.13
INSTRUM             spect
PROBHD    5 mm PABBO BB/
PULPROG              zg30
TD                  65536
SOLVENT              MeOD
NS                     16
DS                      2
SWH            10000.000 Hz
FIDRES          0.152588 Hz
AQ             3.2767999 sec
RG                  30.85
DW                 50.000 usec
DE                   6.50 usec
TE                  303.0 K
D1            1.00000000 sec
TD0                     1

======== CHANNEL f1 ========
SFO1         500.2030889 MHz
NUC1                   1H
P1                  10.00 usec
PLW1          22.00000000 W

F2 - Processing parameters
SI                  65536
SF           500.2000002 MHz
WDW                    EM
SSB         0
LB                   0.30 Hz
GB          0
PC                   1.00
```

CB3-MeOD-1H

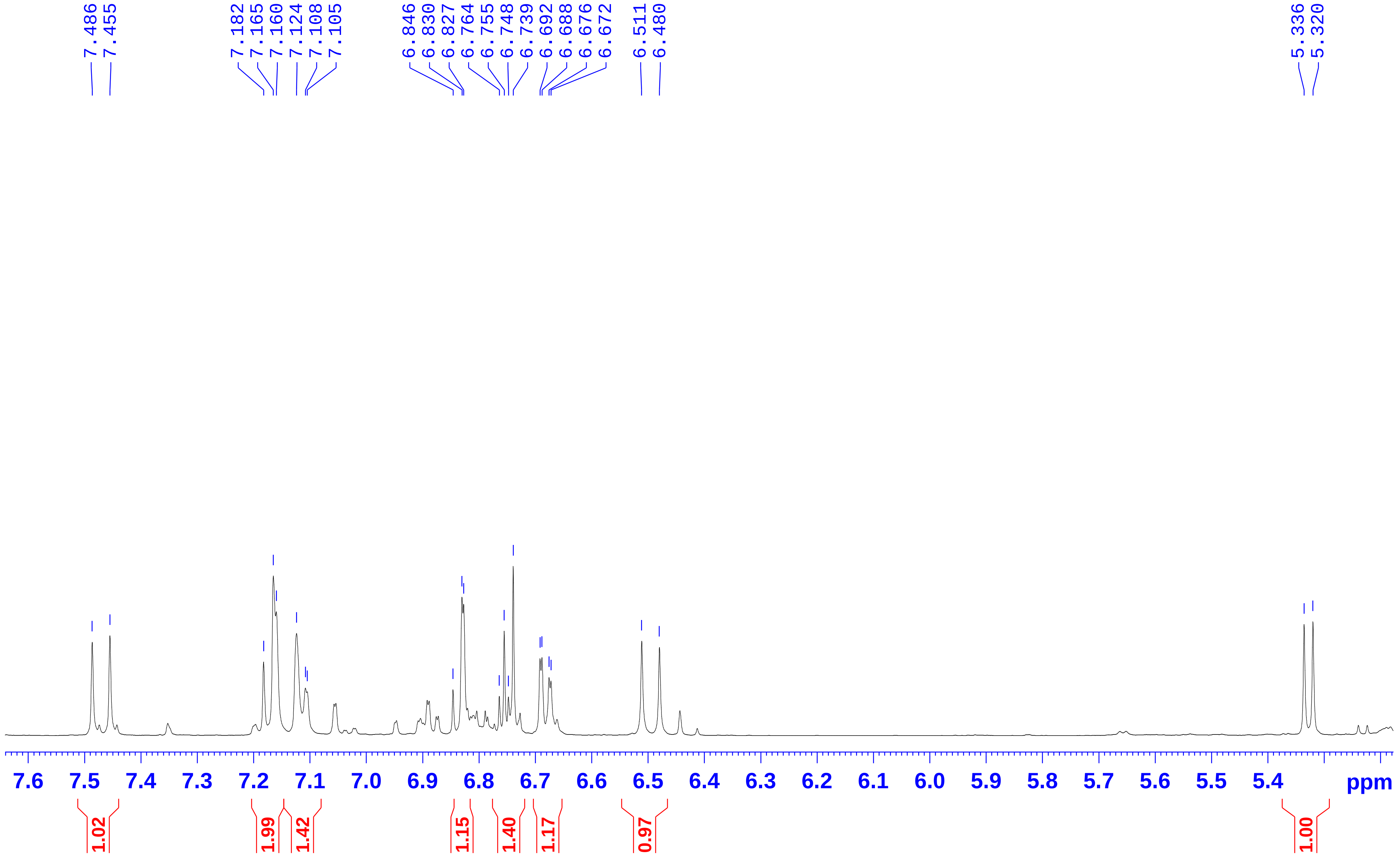

7.486
7.455
7.182
7.165
7.160
7.124
7.108
7.105
6.846
6.830
6.827
6.764
6.755
6.748
6.739
6.692
6.688
6.676
6.672
6.511
6.480
5.336
5.320
7.6 7.5 7.4 7.3 7.2 7.1 7.0 6.9 6.8 6.7 6.6 6.5 6.4 6.3 6.2 6.1 6.0 5.9 5.8 5.7 5.6 5.5 5.4 ppm
1.02
1.99
1.42
1.15
1.40
1.17
0.97
1.00

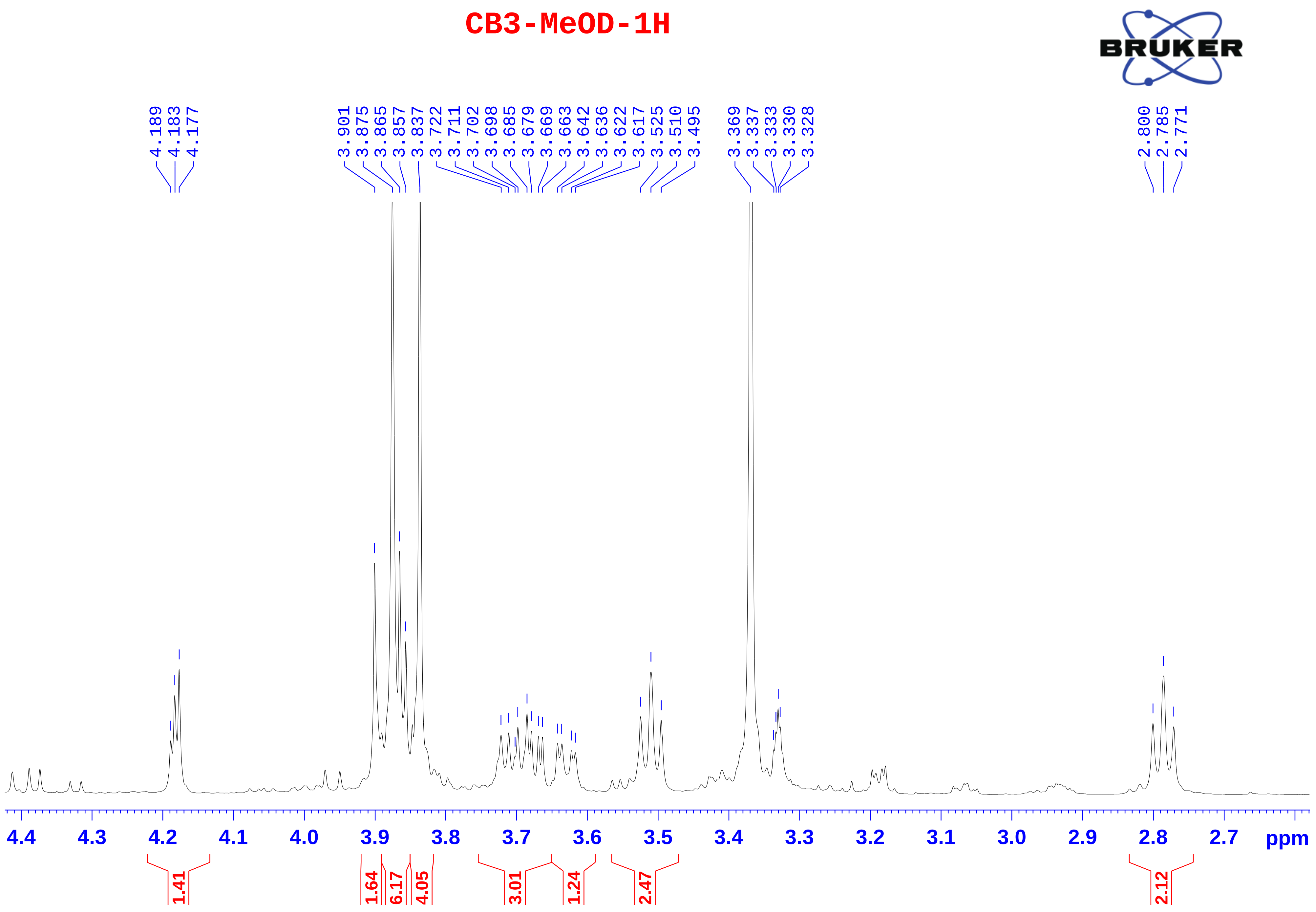
CB3-MeOD-1H
4.189
4.183
4.177
3.901
3.875
3.865
3.857
3.837
3.722
3.711
3.702
3.698
3.685
3.679
3.669
3.663
3.642
3.636
3.622
3.617
3.525
3.510
3.495
3.369
3.337
3.333
3.330
3.328
2.800
2.785
2.771
4.4
4.3
4.2
4.1
4.0
3.9
3.8
3.7
3.6
3.5
3.4
3.3
3.2
3.1
3.0
2.9
2.8
2.7
ppm
1.41
1.64
6.17
4.05
3.01
1.24
2.47
2.12
