# Supplementary material for: Chemical Constituents from Cimicifuga dahurica and Their Anti-Proliferative Effects on MCF-7 Breast Cancer Cells
Source: Molecules. 2018 May 4;23(5):1083. doi: 10.3390/molecules23051083 (PMC6102574; doi:10.3390/molecules23051083)
Supplement: Supplementary file 1 [file molecules-23-01083-s001.zip › Supplementary Materials_liping/Figure S6. COSY spectrum of compound 3.pdf]

CB3-MeOD-COSYGP

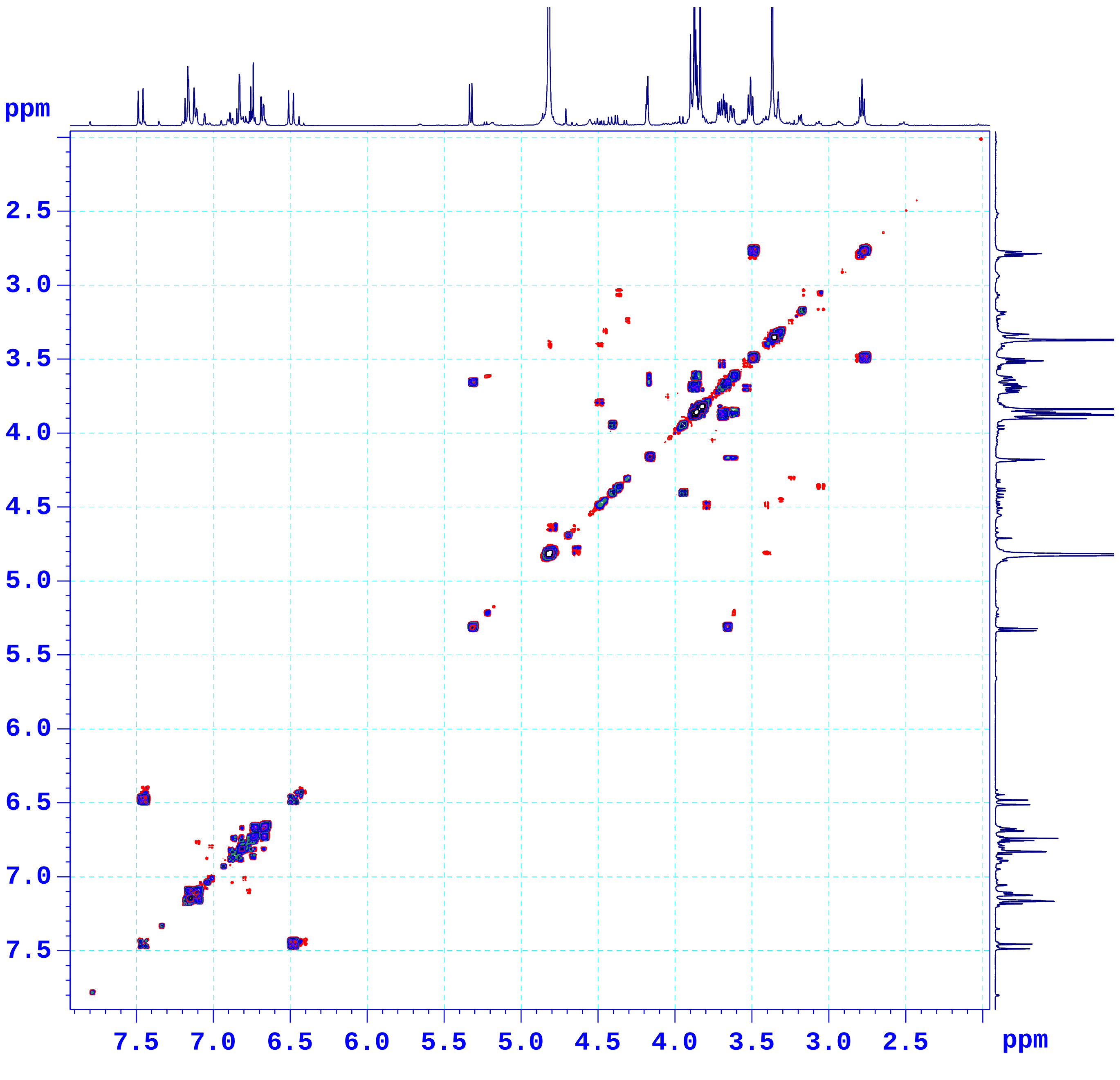

Current Data Parameters
NAME 11D_CB3
EXPNO 7
PROCNO 1

F2 - Acquisition Parameters
Date_ 20170608
Time 13.51
INSTRUM spect
PROBHD 5 mm PABBO BB/
PULPROG cosygpppqf
TD 2048
SOLVENT MeOD
NS 2
DS 8
SWH 3906.250 Hz
FIDRES 1.907349 Hz
AQ 0.2621440 sec
RG 19.38
DW 128.000 usec
DE 6.50 usec
TE 301.9 K
D0 0.00000300 sec
D1 1.89104605 sec
D11 0.03000000 sec
D12 0.00002000 sec
D13 0.00000400 sec
D16 0.00020000 sec
IN0 0.00025600 sec

======== CHANNEL f1 ========
SFO1 500.2021239 MHz
NUC1 1H
P0 10.00 usec
P1 10.00 usec
P17 2500.00 usec
PLW1 22.00000000 W
PLW10 3.25440001 W

====== GRADIENT CHANNEL =====
GPNAM[1] SMSQ10.100
GPZ1 10.00 %
P16 1000.00 usec

F1 - Acquisition parameters
TD 128
SFO1 500.2021 MHz
FIDRES 30.517578 Hz
SW 7.809 ppm
FnMODE QF

F2 - Processing parameters
SI 1024
SF 500.2000122 MHz
WDW QSINE
SSB 0
LB 0 Hz
GB 0
PC 1.40

F1 - Processing parameters
SI 1024
MC2 QF
SF 500.2000122 MHz
WDW QSINE
SSB 0
LB 0 Hz
GB 0

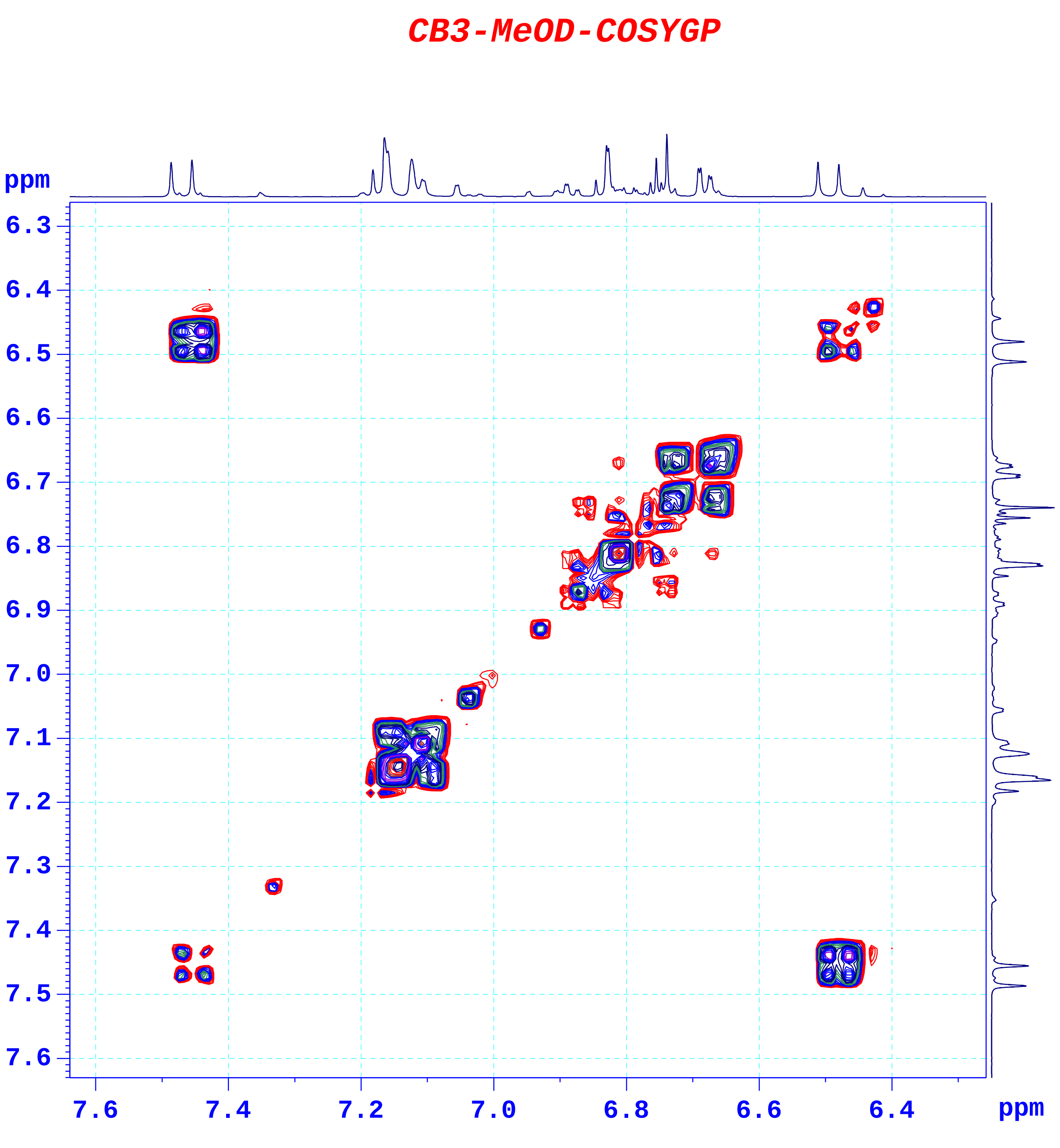


```
Current Data Parameters
NAME              11D_CB3
EXPNO                   7
PROCNO                  1

F2 - Acquisition Parameters
Date_            20170608
Time                13.51
INSTRUM             spect
PROBHD     5 mm PABBO BB/
PULPROG        cosygpppqf
TD                   2048
SOLVENT              MeOD
NS                      2
DS                      8
SWH              3906.250 Hz
FIDRES           1.907349 Hz
AQ              0.2621440 sec
RG                  19.38
DW                128.000 usec
DE                   6.50 usec
TE                  301.9 K
D0             0.00000300 sec
D1             1.89104605 sec
D11            0.03000000 sec
D12            0.00002000 sec
D13            0.00000400 sec
D16            0.00020000 sec
IN0            0.00025600 sec

======== CHANNEL f1 ========
SFO1          500.2021239 MHz
NUC1                   1H
P0                  10.00 usec
P1                  10.00 usec
P17               2500.00 usec
PLW1          22.00000000 W
PLW10          3.25440001 W

====== GRADIENT CHANNEL =====
GPNAM[1]       SMSQ10.100
GPZ1                10.00 %
P16               1000.00 usec

F1 - Acquisition parameters
TD                    128
SFO1             500.2021 MHz
FIDRES          30.517578 Hz
SW                  7.809 ppm
FnMODE                 QF

F2 - Processing parameters
SI                   1024
SF            500.2000122 MHz
WDW                 QSINE
SSB        0
LB         0 Hz
GB         0
PC                   1.40

F1 - Processing parameters
SI                   1024
MC2                    QF
SF            500.2000122 MHz
WDW                 QSINE
SSB        0
LB         0 Hz
GB         0
```

CB3-MeOD-COSYGP

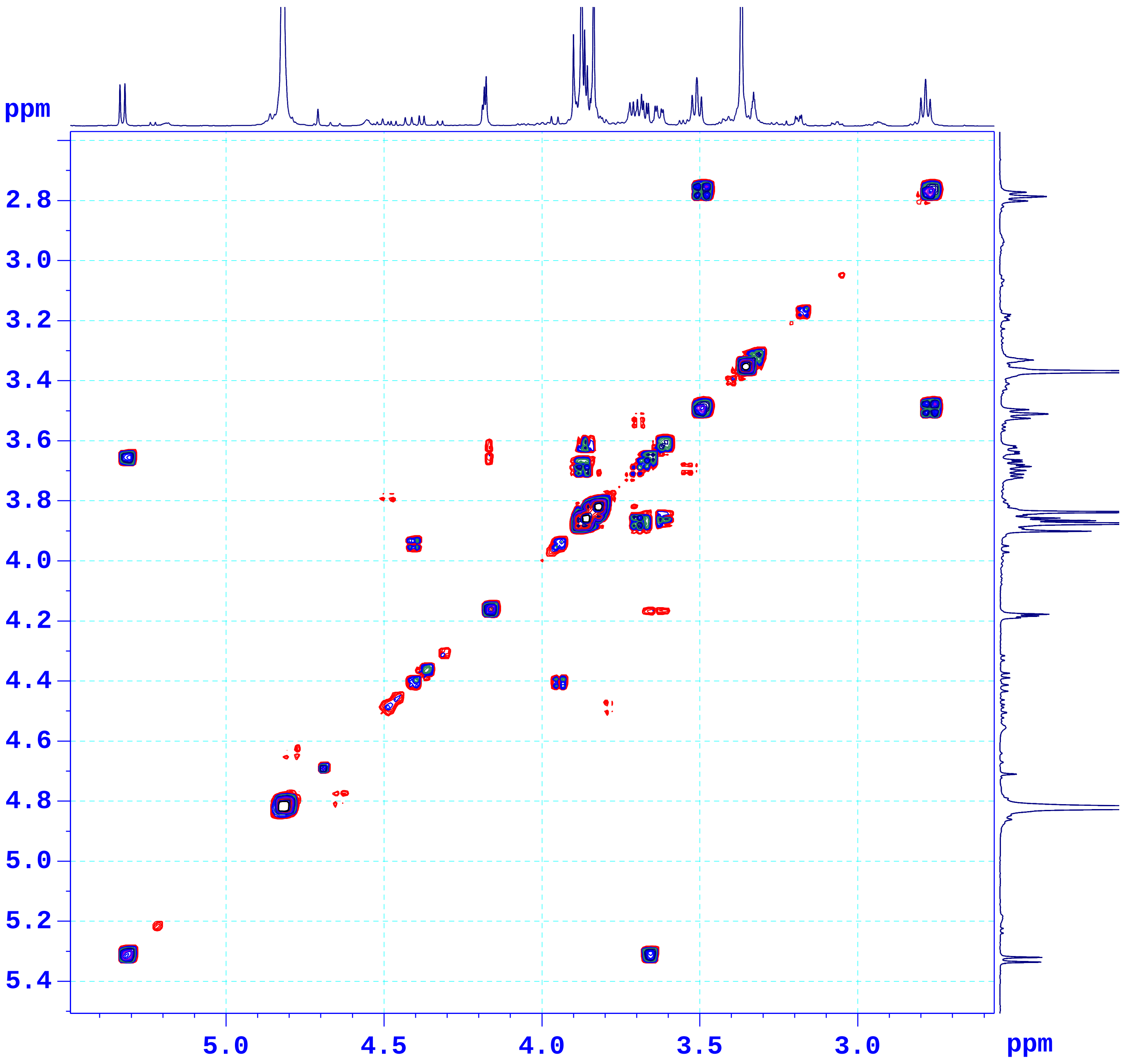

BRUKER

Current Data Parameters
NAME 11D_CB3
EXPNO 7
PROCNO 1

F2 - Acquisition Parameters
Date_ 20170608
Time 13.51
INSTRUM spect
PROBHD 5 mm PABBO BB/
PULPROG cosygpppqf
TD 2048
SOLVENT MeOD
NS 2
DS 8
SWH 3906.250 Hz
FIDRES 1.907349 Hz
AQ 0.2621440 sec
RG 19.38
DW 128.000 usec
DE 6.50 usec
TE 301.9 K
D0 0.00000300 sec
D1 1.89104605 sec
D11 0.03000000 sec
D12 0.00002000 sec
D13 0.00000400 sec
D16 0.00020000 sec
IN0 0.00025600 sec

======== CHANNEL f1 ========
SFO1 500.2021239 MHz
NUC1 1H
P0 10.00 usec
P1 10.00 usec
P17 2500.00 usec
PLW1 22.00000000 W
PLW10 3.25440001 W

====== GRADIENT CHANNEL =====
GPNAM[1] SMSQ10.100
GPZ1 10.00 %
P16 1000.00 usec

F1 - Acquisition parameters
TD 128
SFO1 500.2021 MHz
FIDRES 30.517578 Hz
SW 7.809 ppm
FnMODE QF

F2 - Processing parameters
SI 1024
SF 500.2000122 MHz
WDW QSINE
SSB 0
LB 0 Hz
GB 0
PC 1.40

F1 - Processing parameters
SI 1024
MC2 QF
SF 500.2000122 MHz
WDW QSINE
SSB 0
LB 0 Hz
GB 0

CB3-MeOD-COSYGP

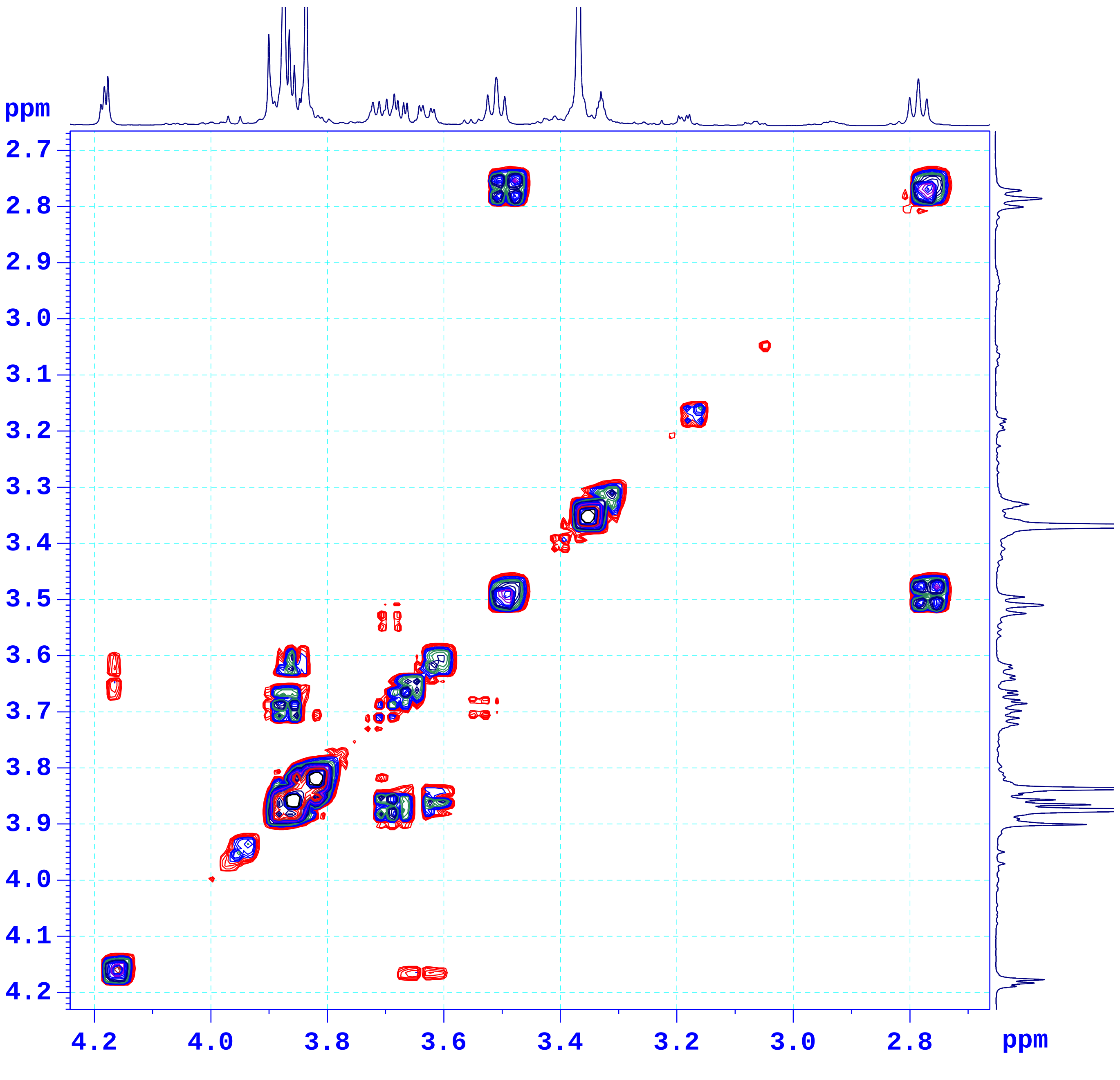


```
Current Data Parameters
NAME              11D_CB3
EXPNO                   7
PROCNO                  1

F2 - Acquisition Parameters
Date_            20170608
Time                13.51
INSTRUM             spect
PROBHD     5 mm PABBO BB/
PULPROG        cosygpppqf
TD                   2048
SOLVENT              MeOD
NS                      2
DS                      8
SWH              3906.250 Hz
FIDRES           1.907349 Hz
AQ              0.2621440 sec
RG                  19.38
DW                128.000 usec
DE                   6.50 usec
TE                  301.9 K
D0             0.00000300 sec
D1             1.89104605 sec
D11            0.03000000 sec
D12            0.00002000 sec
D13            0.00000400 sec
D16            0.00020000 sec
IN0            0.00025600 sec

======== CHANNEL f1 ========
SFO1          500.2021239 MHz
NUC1                   1H
P0                  10.00 usec
P1                  10.00 usec
P17               2500.00 usec
PLW1          22.00000000 W
PLW10          3.25440001 W

====== GRADIENT CHANNEL =====
GPNAM[1]        SMSQ10.100
GPZ1                 10.00 %
P16                1000.00 usec

F1 - Acquisition parameters
TD                    128
SFO1             500.2021 MHz
FIDRES          30.517578 Hz
SW                  7.809 ppm
FnMODE                 QF

F2 - Processing parameters
SI                   1024
SF            500.2000122 MHz
WDW                 QSINE
SSB       0
LB        0 Hz
GB        0
PC                   1.40

F1 - Processing parameters
SI                   1024
MC2                    QF
SF            500.2000122 MHz
WDW                 QSINE
SSB       0
LB        0 Hz
GB        0
```
