# Supplementary material for: Chemical Constituents from Cimicifuga dahurica and Their Anti-Proliferative Effects on MCF-7 Breast Cancer Cells
Source: Molecules. 2018 May 4;23(5):1083. doi: 10.3390/molecules23051083 (PMC6102574; doi:10.3390/molecules23051083)
Supplement: Supplementary file 1 [file molecules-23-01083-s001.zip › Supplementary Materials_liping/Figure S7. 1H spectrum of compound 4.pdf]

# CB4-MeOD-1H

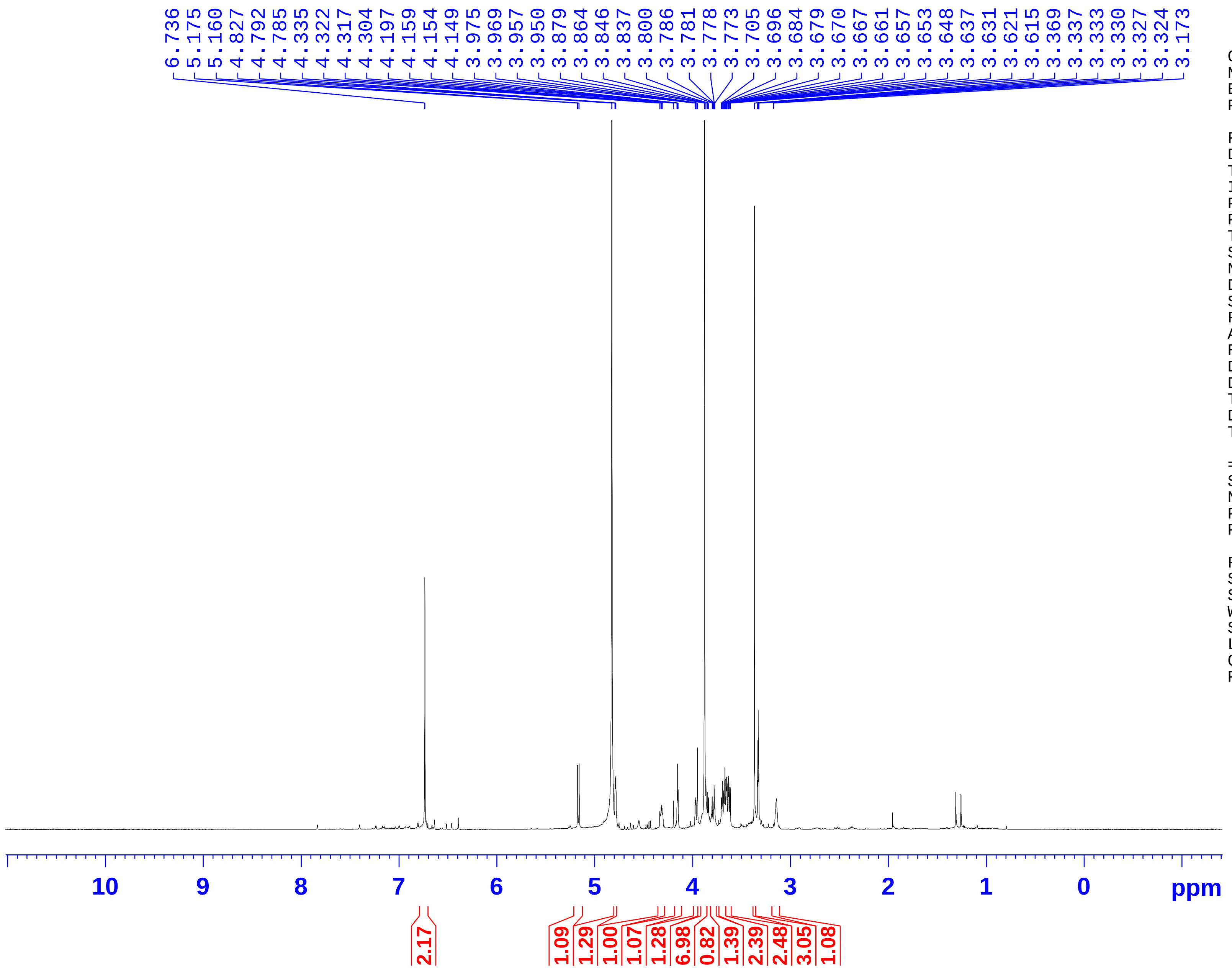


| Current Data Parameters | |
|---|---|
| NAME | 11D_CB4 |
| EXPNO | 1 |
| PROCNO | 1 |
| **F2 - Acquisition Parameters** | |
| Date_ | 20170601 |
| Time | 16.18 |
| INSTRUM | spect |
| PROBHD | 5 mm PABBO BB/ |
| PULPROG | zg30 |
| TD | 65536 |
| SOLVENT | MeOD |
| NS | 16 |
| DS | 2 |
| SWH | 10000.000 Hz |
| FIDRES | 0.152588 Hz |
| AQ | 3.2767999 sec |
| RG | 79.36 |
| DW | 50.000 usec |
| DE | 6.50 usec |
| TE | 303.0 K |
| D1 | 1.00000000 sec |
| TD0 | 1 |
| **======== CHANNEL f1 ========** | |
| SFO1 | 500.2030889 MHz |
| NUC1 | 1H |
| P1 | 10.00 usec |
| PLW1 | 22.00000000 W |
| **F2 - Processing parameters** | |
| SI | 65536 |
| SF | 500.2000004 MHz |
| WDW | EM |
| SSB | 0 |
| LB | 0.30 Hz |
| GB | 0 |
| PC | 1.00 |

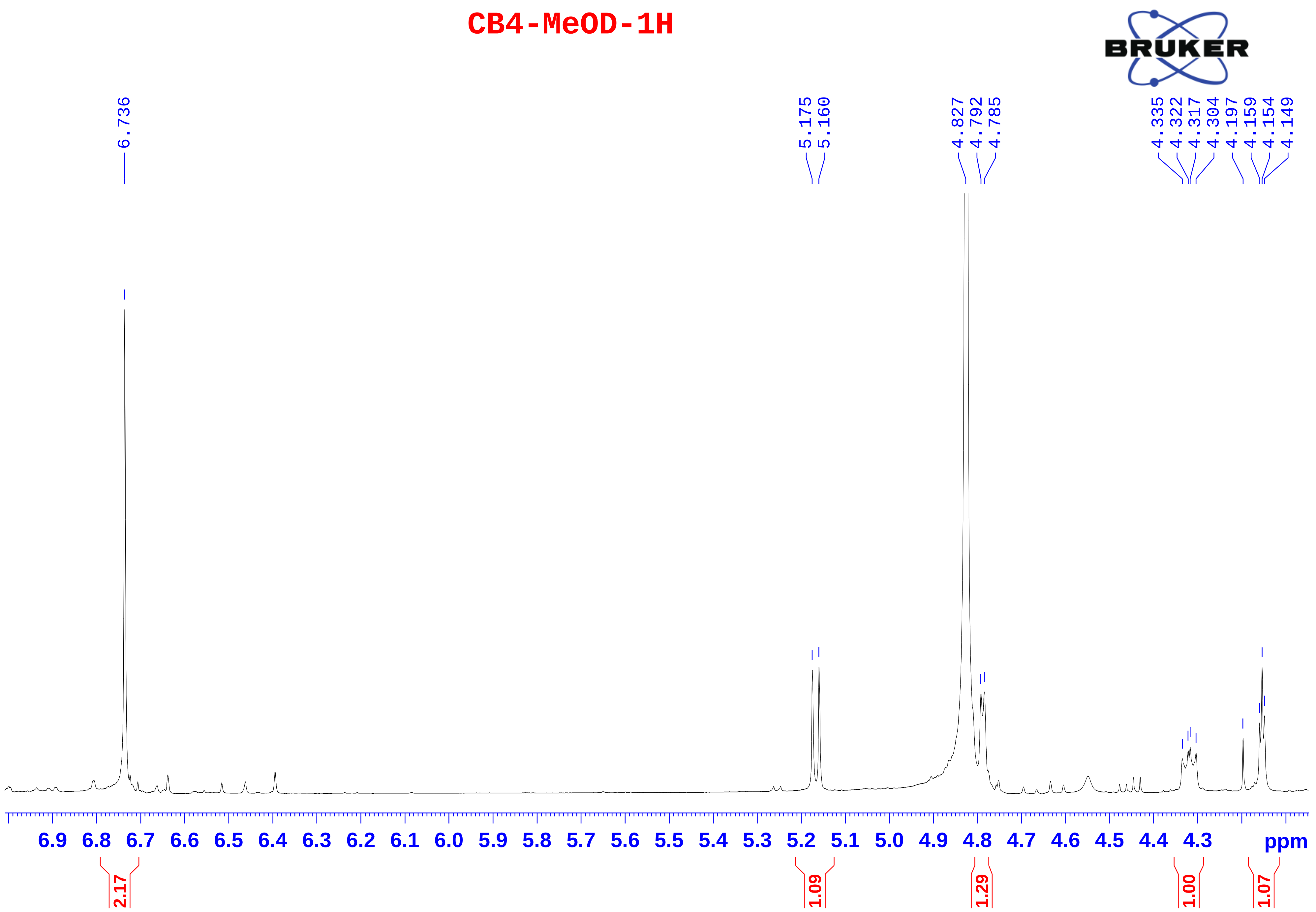
CB4-MeOD-1H
BRUKER
6.736
5.175
5.160
4.827
4.792
4.785
4.335
4.322
4.317
4.304
4.197
4.159
4.154
4.149
6.9 6.8 6.7 6.6 6.5 6.4 6.3 6.2 6.1 6.0 5.9 5.8 5.7 5.6 5.5 5.4 5.3 5.2 5.1 5.0 4.9 4.8 4.7 4.6 4.5 4.4 4.3
ppm
2.17
1.09
1.29
1.00
1.07

# CB4-MeOD-1H

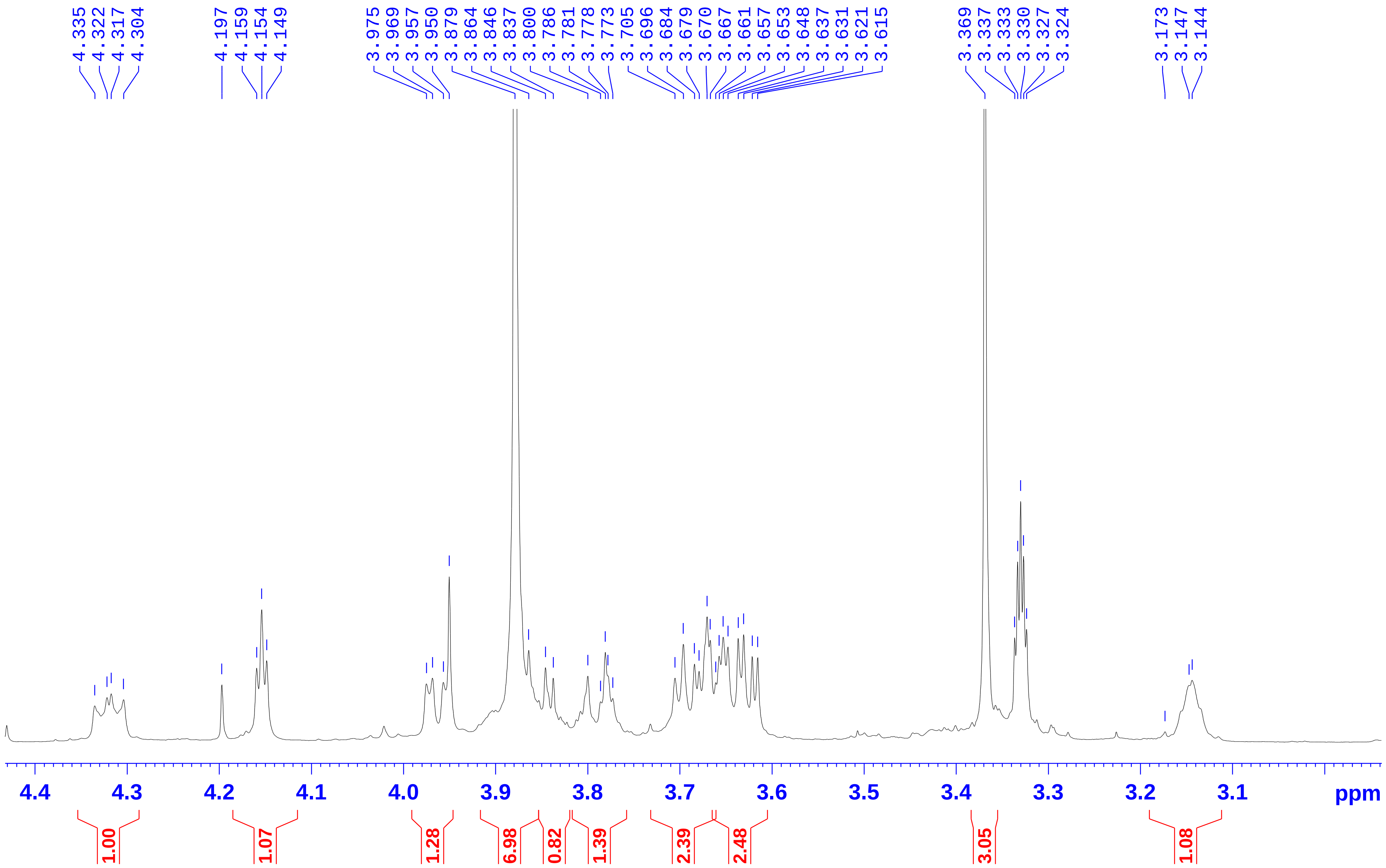

4.335
4.322
4.317
4.304
4.197
4.159
4.154
4.149
3.975
3.969
3.957
3.950
3.879
3.864
3.846
3.837
3.800
3.786
3.781
3.778
3.773
3.705
3.696
3.684
3.679
3.670
3.667
3.661
3.657
3.653
3.648
3.637
3.631
3.621
3.615
3.369
3.337
3.333
3.330
3.327
3.324
3.173
3.147
3.144
4.4
4.3
4.2
4.1
4.0
3.9
3.8
3.7
3.6
3.5
3.4
3.3
3.2
3.1
ppm
1.00
1.07
1.28
6.98
0.82
1.39
2.39
2.48
3.05
1.08
