# Supplementary material for: Chemical Constituents from Cimicifuga dahurica and Their Anti-Proliferative Effects on MCF-7 Breast Cancer Cells
Source: Molecules. 2018 May 4;23(5):1083. doi: 10.3390/molecules23051083 (PMC6102574; doi:10.3390/molecules23051083)
Supplement: Supplementary file 1 [file molecules-23-01083-s001.zip › Supplementary Materials_liping/Figure S8. 13C spectrum of compound 4.pdf]

CB4-MeOD-C13CPD

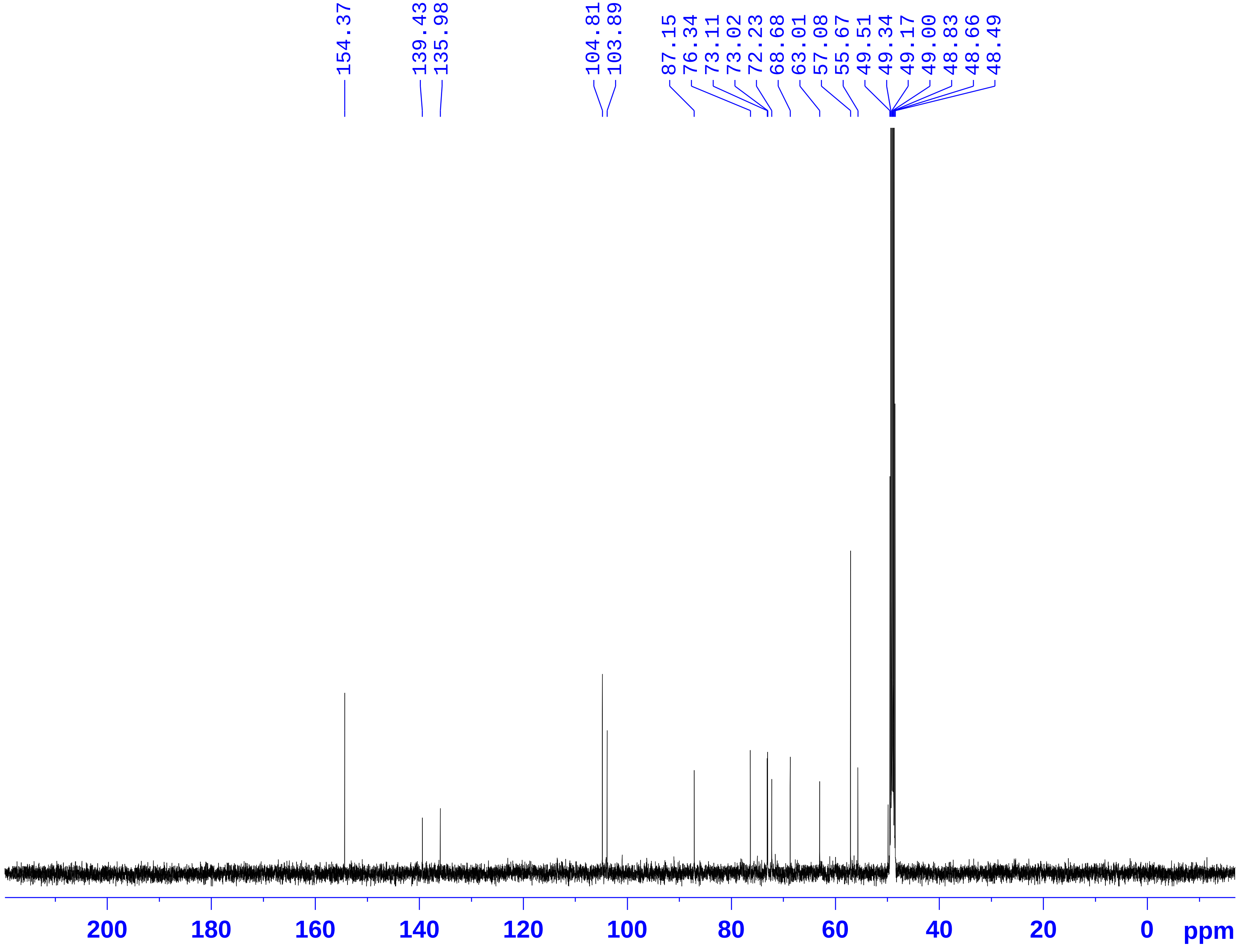

BRUKER

Current Data Parameters
NAME 11D_CB4
EXPNO 2
PROCNO 1

F2 - Acquisition Parameters
Date_ 20170602
Time 16.28
INSTRUM spect
PROBHD 5 mm PABBO BB/
PULPROG zgpg30
TD 65536
SOLVENT MeOD
NS 128
DS 4
SWH 29761.904 Hz
FIDRES 0.454131 Hz
AQ 1.1010048 sec
RG 198.57
DW 16.800 usec
DE 6.50 usec
TE 303.2 K
D1 2.00000000 sec
D11 0.03000000 sec
TD0 1

======== CHANNEL f1 ========
SFO1 125.7879670 MHz
NUC1 13C
P1 10.00 usec
PLW1 88.00000000 W

======== CHANNEL f2 ========
SFO2 500.2020008 MHz
NUC2 1H
CPDPRG[2 waltz16
PCPD2 80.00 usec
PLW2 22.00000000 W
PLW12 0.34375000 W
PLW13 0.22000000 W

F2 - Processing parameters
SI 32768
SF 125.7752160 MHz
WDW EM
SSB 0
LB 1.00 Hz
GB 0
PC 1.40

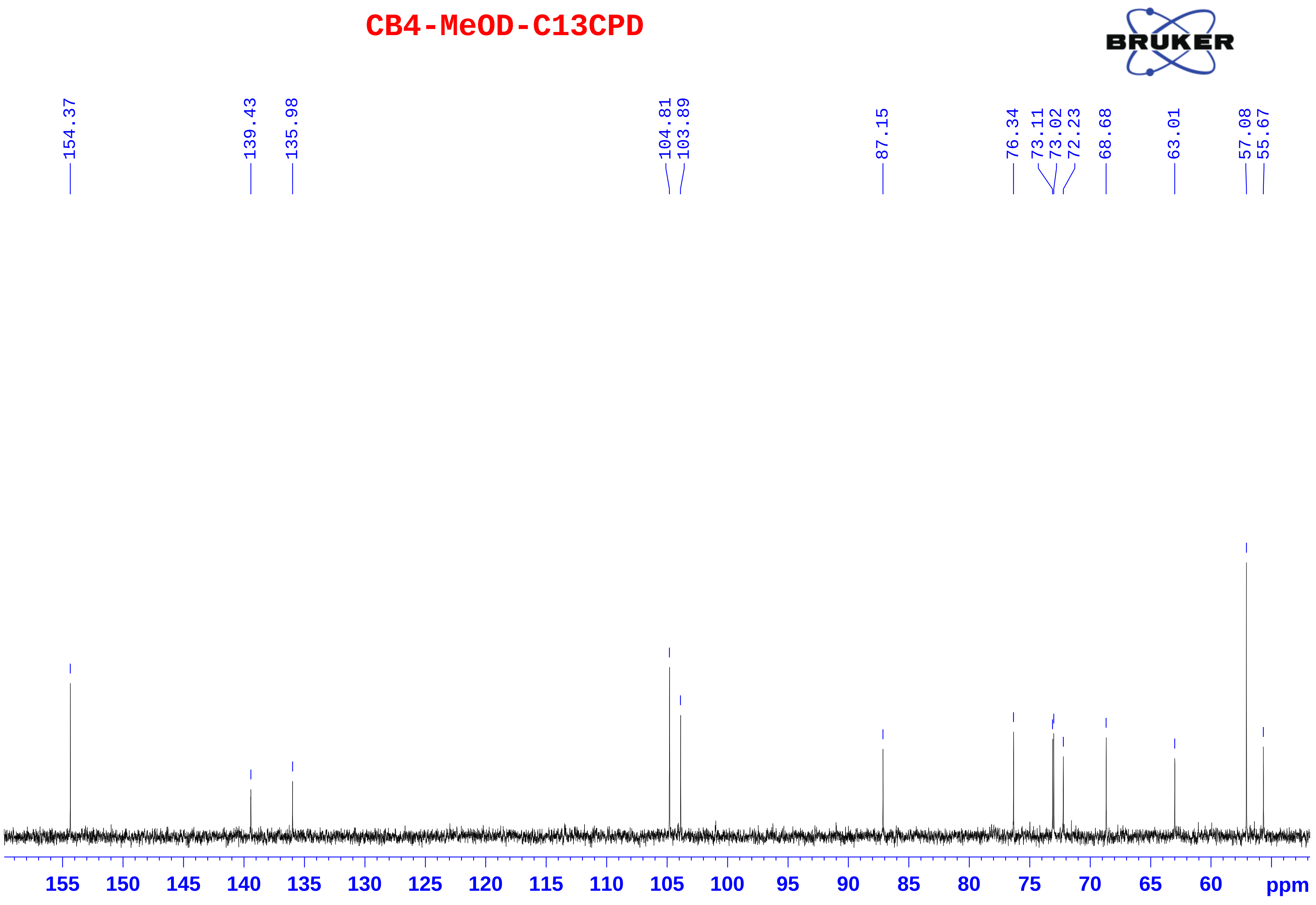
CB4-MeOD-C13CPD
BRUKER
154.37
139.43
135.98
104.81
103.89
87.15
76.34
73.11
73.02
72.23
68.68
63.01
57.08
55.67
155
150
145
140
135
130
125
120
115
110
105
100
95
90
85
80
75
70
65
60
ppm
