# Supplementary material for: Chemical Constituents from Cimicifuga dahurica and Their Anti-Proliferative Effects on MCF-7 Breast Cancer Cells
Source: Molecules. 2018 May 4;23(5):1083. doi: 10.3390/molecules23051083 (PMC6102574; doi:10.3390/molecules23051083)
Supplement: Supplementary file 1 [file molecules-23-01083-s001.zip › Supplementary Materials_liping/Figure S12. ROESY spectrum of compound 4.pdf]

CB4-MeOD-ROESY

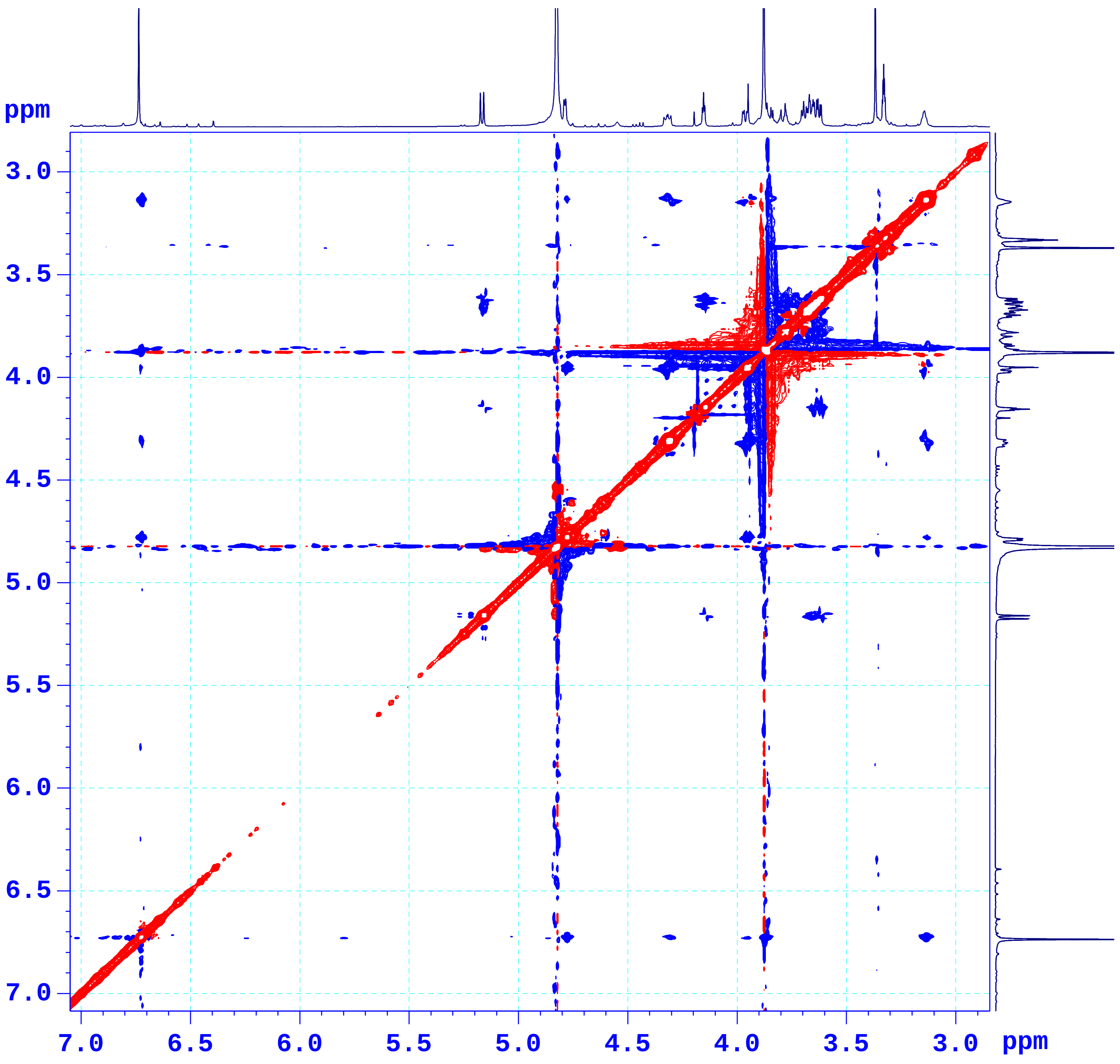

BRUKER

Current Data Parameters

NAME	11D_CB4
EXPNO	7
PROCNO	1

F2 - Acquisition Parameters

Date_	20170608
Time	14.42
INSTRUM	spect
PROBHD	5 mm PABBO BB/
PULPROG	roesyphpp.2
TD	2048
SOLVENT	MeOD
NS	4
DS	32
SWH	3816.794 Hz
FIDRES	1.863669 Hz
AQ	0.2682880 sec
RG	44.31
DW	131.000 usec
DE	6.50 usec
TE	302.0 K
D0	0.00012063 sec
D1	1.93651199 sec
D11	0.03000000 sec
D12	0.00002000 sec
IN0	0.00026200 sec
L4	1000
P15	200000.00 usec

======== CHANNEL f1 ========

SFO1	500.2020570 MHz
NUC1	1H
P1	10.00 usec
P17	2500.00 usec
P25	100.00 usec
PLW1	22.00000000 W
PLW10	3.25440001 W
PLW27	0.88000000 W

F1 - Acquisition parameters

TD	256
SFO1	500.2021 MHz
FIDRES	14.909351 Hz
SW	7.631 ppm
FnMODE	States-TPPI

F2 - Processing parameters

SI	1024
SF	500.2000000 MHz
WDW	QSINE
SSB	2
LB	0 Hz
GB	0
PC	1.00

F1 - Processing parameters

SI	1024
MC2	States-TPPI
SF	500.2000000 MHz
WDW	QSINE
SSB	2
LB	0 Hz
GB	0

CB4-MeOD-ROESY

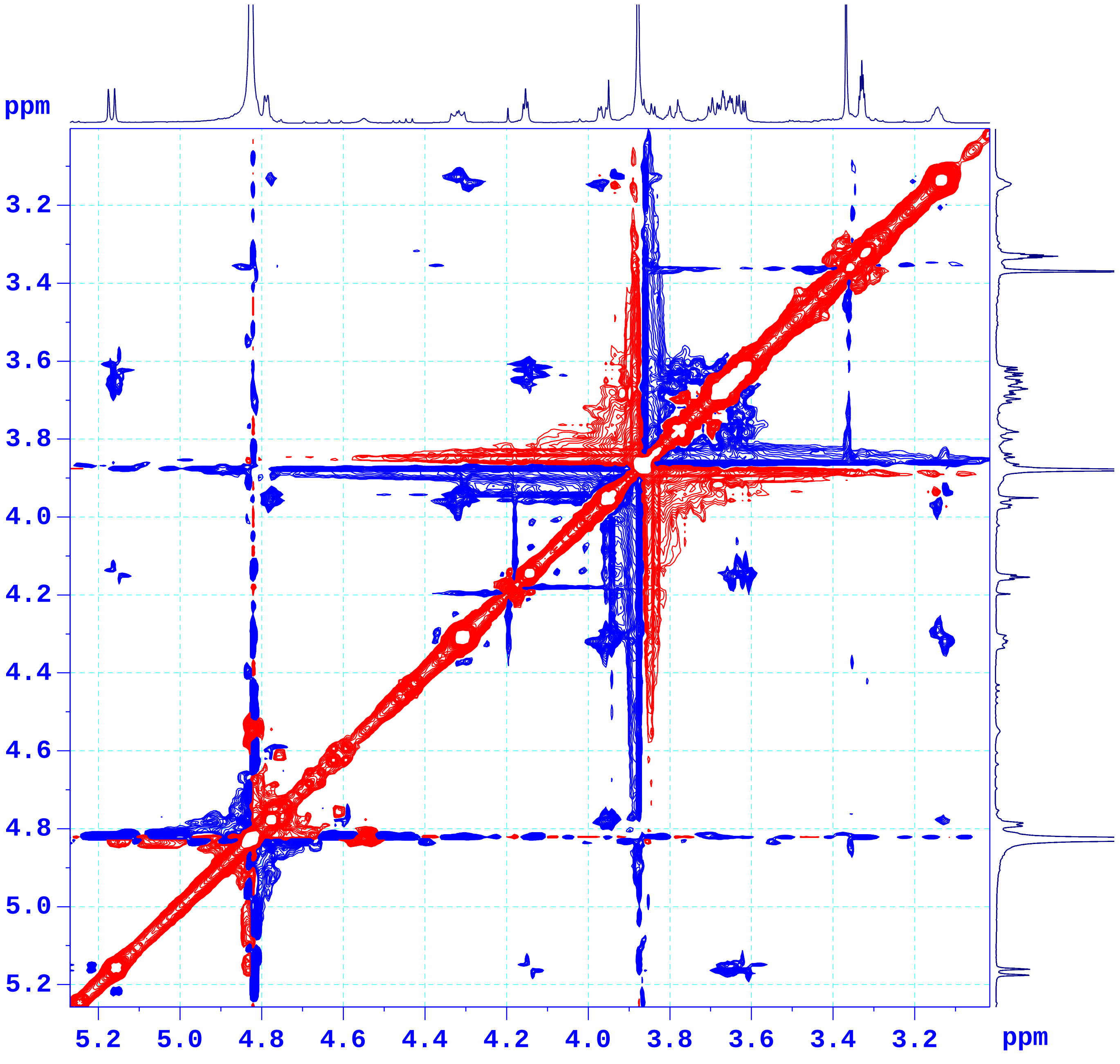

BRUKER

Current Data Parameters

NAME	11D_CB4
EXPNO	7
PROCNO	1

F2 - Acquisition Parameters

Date_	20170608
Time	14.42
INSTRUM	spect
PROBHD	5 mm PABBO BB/
PULPROG	roesyphpp.2
TD	2048
SOLVENT	MeOD
NS	4
DS	32
SWH	3816.794 Hz
FIDRES	1.863669 Hz
AQ	0.2682880 sec
RG	44.31
DW	131.000 usec
DE	6.50 usec
TE	302.0 K
D0	0.00012063 sec
D1	1.93651199 sec
D11	0.03000000 sec
D12	0.00002000 sec
IN0	0.00026200 sec
L4	1000
P15	200000.00 usec

======== CHANNEL f1 ========

SFO1	500.2020570 MHz
NUC1	1H
P1	10.00 usec
P17	2500.00 usec
P25	100.00 usec
PLW1	22.00000000 W
PLW10	3.25440001 W
PLW27	0.88000000 W

F1 - Acquisition parameters

TD	256
SFO1	500.2021 MHz
FIDRES	14.909351 Hz
SW	7.631 ppm
FnMODE	States-TPPI

F2 - Processing parameters

SI	1024
SF	500.2000000 MHz
WDW	QSINE
SSB	2
LB	0 Hz
GB	0
PC	1.00

F1 - Processing parameters

SI	1024
MC2	States-TPPI
SF	500.2000000 MHz
WDW	QSINE
SSB	2
LB	0 Hz
GB	0

CB4-MeOD-ROESY

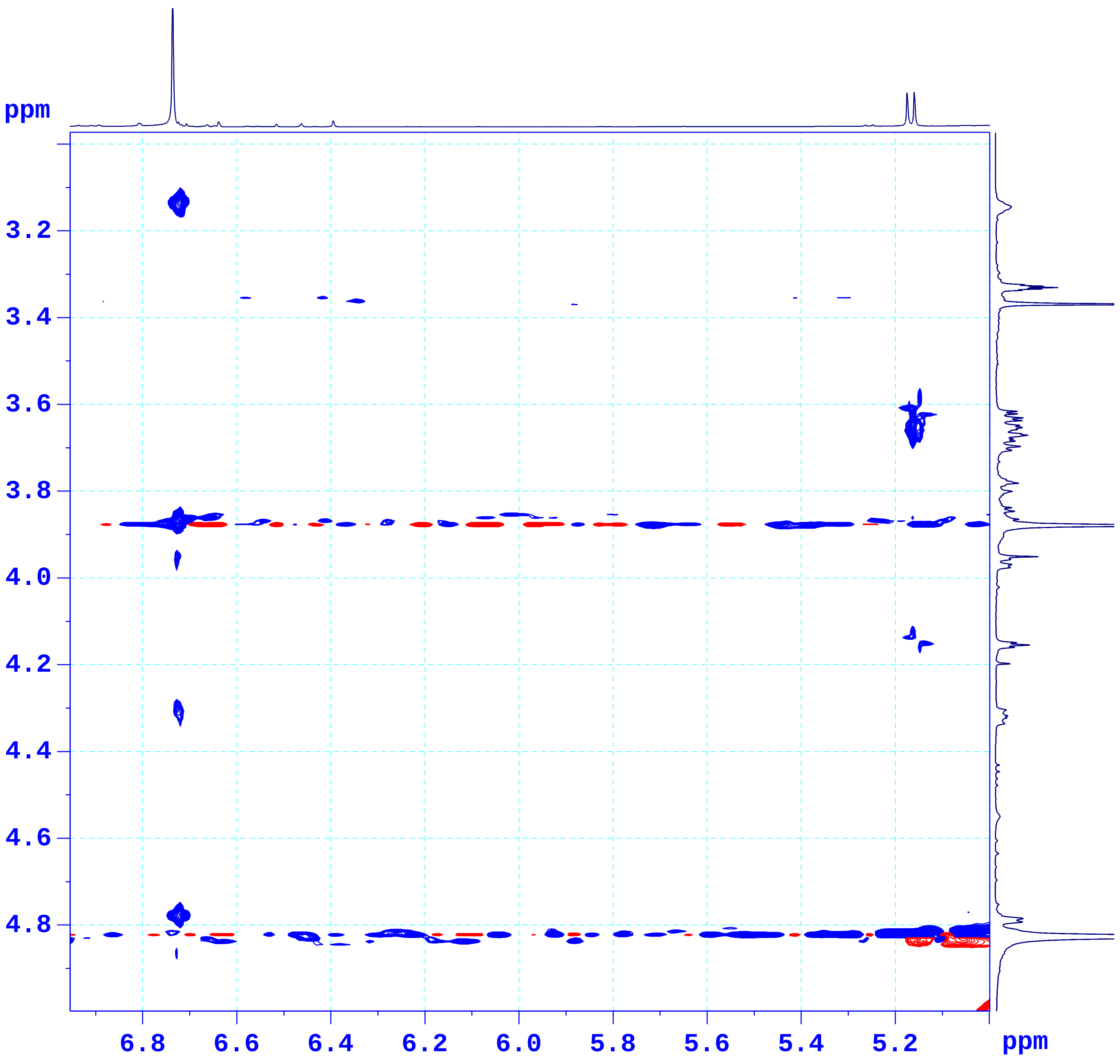


```
Current Data Parameters
NAME                 11D_CB4
EXPNO                      7
PROCNO                     1

F2 - Acquisition Parameters
Date_               20170608
Time                   14.42
INSTRUM                spect
PROBHD     5 mm PABBO BB/
PULPROG         roesyphpp.2
TD                      2048
SOLVENT                 MeOD
NS                         4
DS                        32
SWH                 3816.794 Hz
FIDRES              1.863669 Hz
AQ                 0.2682880 sec
RG                     44.31
DW                   131.000 usec
DE                      6.50 usec
TE                     302.0 K
D0                0.00012063 sec
D1                1.93651199 sec
D11               0.03000000 sec
D12               0.00002000 sec
IN0               0.00026200 sec
L4                      1000
P15                200000.00 usec

======== CHANNEL f1 ========
SFO1             500.2020570 MHz
NUC1                      1H
P1                     10.00 usec
P17                  2500.00 usec
P25                   100.00 usec
PLW1             22.00000000 W
PLW10             3.25440001 W
PLW27             0.88000000 W

F1 - Acquisition parameters
TD                       256
SFO1                500.2021 MHz
FIDRES             14.909351 Hz
SW                     7.631 ppm
FnMODE            States-TPPI

F2 - Processing parameters
SI                      1024
SF               500.2000000 MHz
WDW                    QSINE
SSB                        2
LB            0 Hz
GB            0
PC                      1.00

F1 - Processing parameters
SI                      1024
MC2               States-TPPI
SF               500.2000000 MHz
WDW                    QSINE
SSB                        2
LB            0 Hz
GB            0
```
