# Supplementary material for: Chemical Constituents from Cimicifuga dahurica and Their Anti-Proliferative Effects on MCF-7 Breast Cancer Cells
Source: Molecules. 2018 May 4;23(5):1083. doi: 10.3390/molecules23051083 (PMC6102574; doi:10.3390/molecules23051083)
Supplement: Supplementary file 1 [file molecules-23-01083-s001.zip › Supplementary Materials_liping/Figure S13. 1H spectrum of compound 6.pdf]

CB6-MeOD-1H

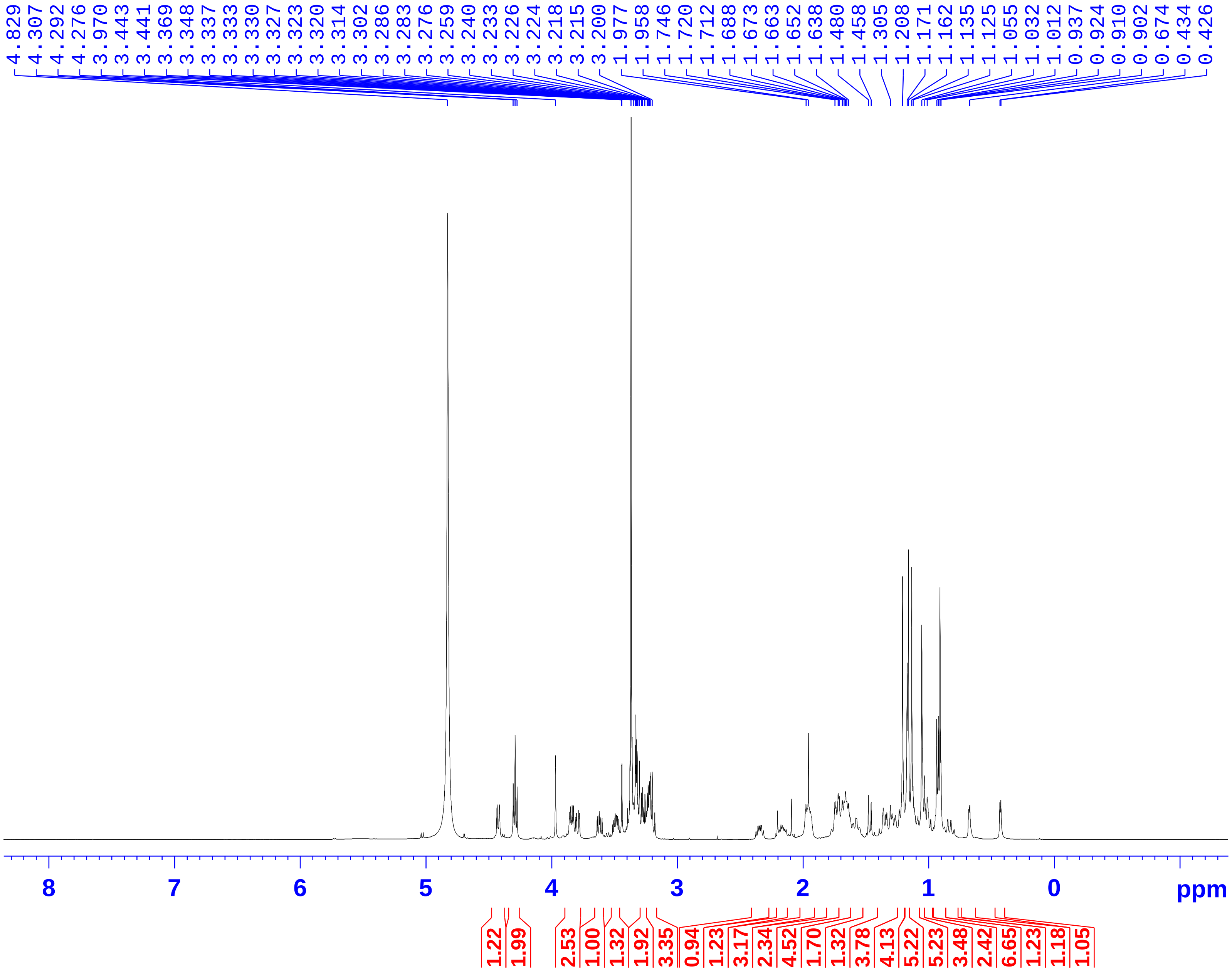

Current Data Parameters

NAME	11D__CB6
EXPNO	1
PROCNO	1

F2 - Acquisition Parameters

Date_	20170610
Time	16.48
INSTRUM	spect
PROBHD	5 mm PABBO BB/
PULPROG	zg30
TD	65536
SOLVENT	MeOD
NS	16
DS	2
SWH	10000.000 Hz
FIDRES	0.152588 Hz
AQ	3.2767999 sec
RG	30.85
DW	50.000 usec
DE	6.50 usec
TE	303.0 K
D1	1.00000000 sec
TD0	1

======== CHANNEL f1 ========

SFO1	500.2030889 MHz
NUC1	1H
P1	10.00 usec
PLW1	22.00000000 W

F2 - Processing parameters

SI	65536
SF	500.2000007 MHz
WDW	EM
SSB	0
LB	0.30 Hz
GB	0
PC	1.00

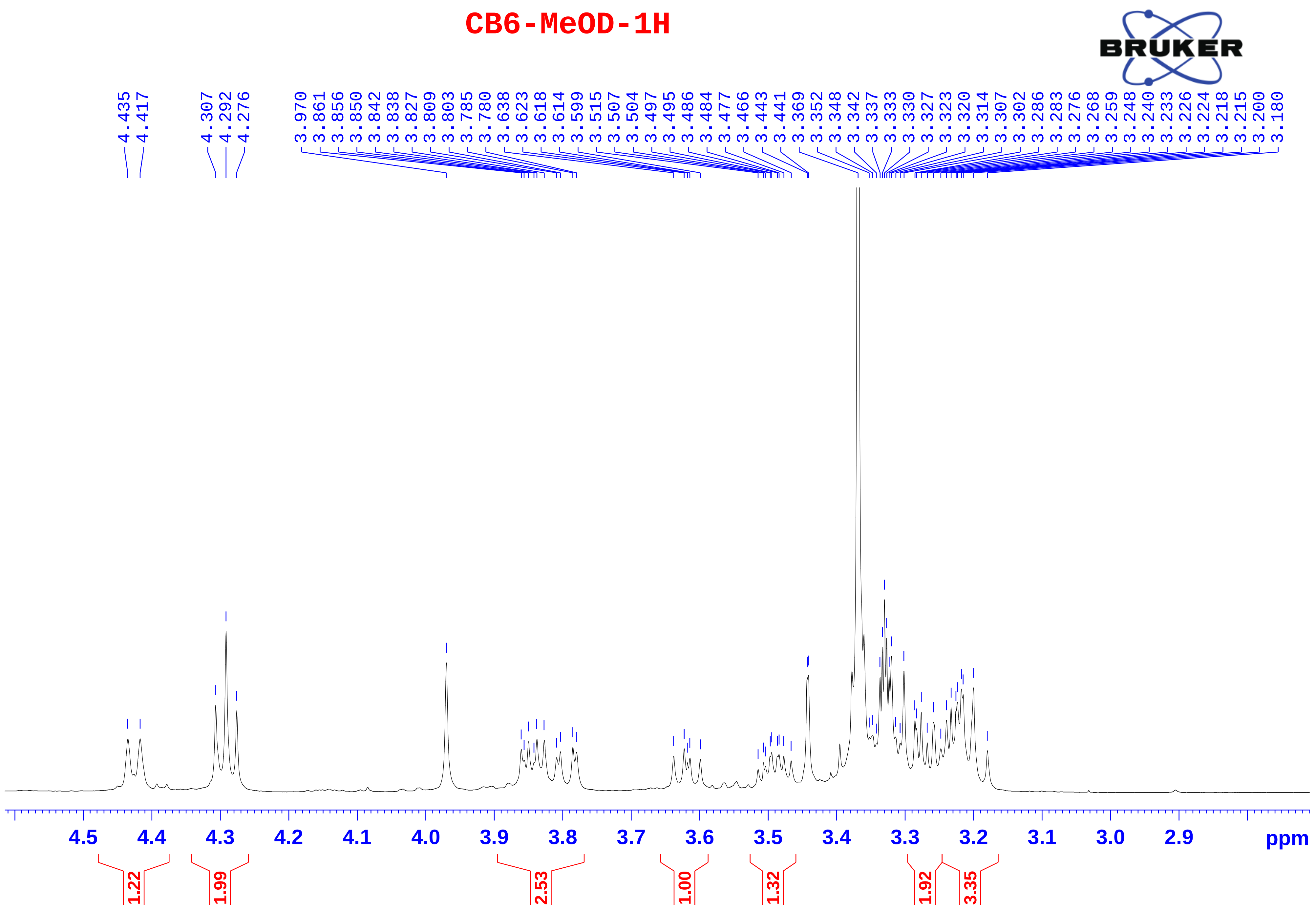
CB6-MeOD-1H
4.435
4.417
4.307
4.292
4.276
3.970
3.861
3.856
3.850
3.842
3.838
3.827
3.809
3.803
3.785
3.780
3.638
3.623
3.618
3.614
3.599
3.515
3.507
3.504
3.497
3.495
3.486
3.484
3.477
3.466
3.443
3.441
3.369
3.352
3.348
3.342
3.337
3.333
3.330
3.327
3.323
3.320
3.314
3.307
3.302
3.286
3.283
3.276
3.268
3.259
3.248
3.240
3.233
3.226
3.224
3.218
3.215
3.200
3.180
4.5
4.4
4.3
4.2
4.1
4.0
3.9
3.8
3.7
3.6
3.5
3.4
3.3
3.2
3.1
3.0
2.9
ppm
1.22
1.99
2.53
1.00
1.32
1.92
3.35

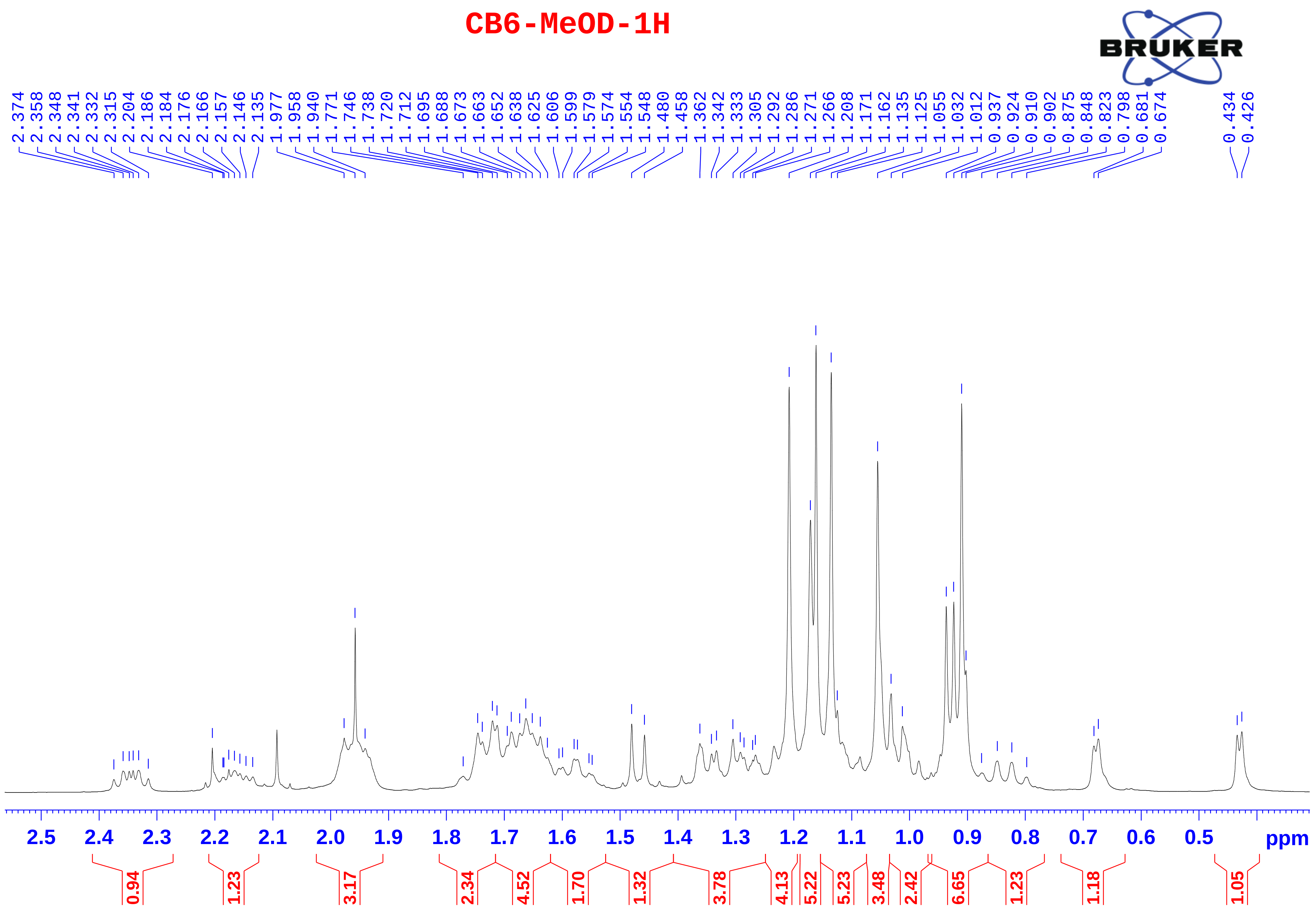

CB6-MeOD-1H
2.374
2.358
2.348
2.341
2.332
2.315
2.204
2.186
2.184
2.176
2.166
2.157
2.146
2.135
1.977
1.958
1.940
1.771
1.746
1.738
1.720
1.712
1.695
1.688
1.673
1.663
1.652
1.638
1.625
1.606
1.599
1.579
1.574
1.554
1.548
1.480
1.458
1.362
1.342
1.333
1.305
1.292
1.286
1.271
1.266
1.208
1.171
1.162
1.135
1.125
1.055
1.032
1.012
0.937
0.924
0.910
0.902
0.875
0.848
0.823
0.798
0.681
0.674
0.434
0.426
2.5
2.4
2.3
2.2
2.1
2.0
1.9
1.8
1.7
1.6
1.5
1.4
1.3
1.2
1.1
1.0
0.9
0.8
0.7
0.6
0.5
ppm
0.94
1.23
3.17
2.34
4.52
1.70
1.32
3.78
4.13
5.22
5.23
3.48
2.42
6.65
1.23
1.18
1.05
