# Supplementary material for: Chemical Constituents from Cimicifuga dahurica and Their Anti-Proliferative Effects on MCF-7 Breast Cancer Cells
Source: Molecules. 2018 May 4;23(5):1083. doi: 10.3390/molecules23051083 (PMC6102574; doi:10.3390/molecules23051083)

CB6-MeOD-C13CPD

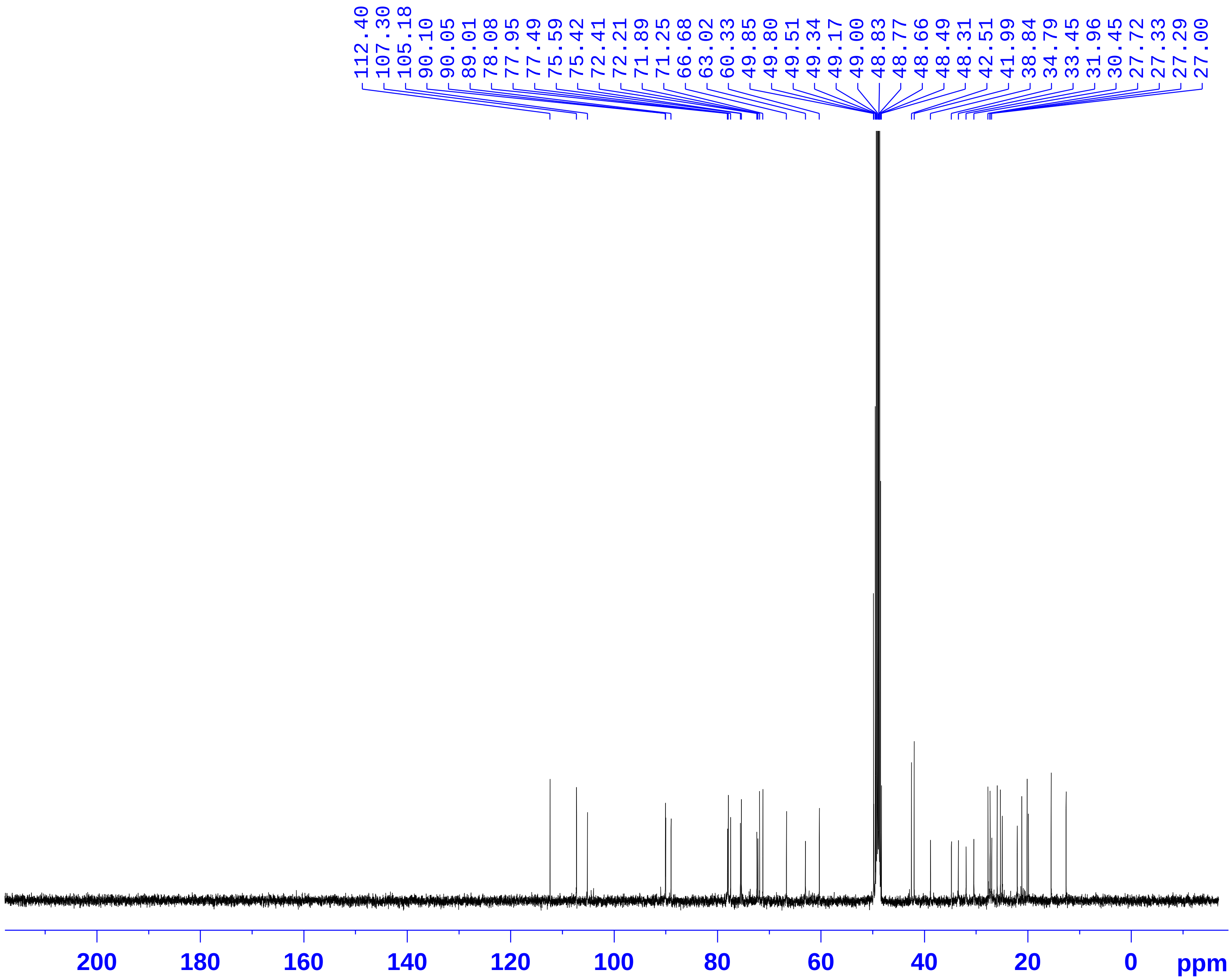

BRUKER

Current Data Parameters

NAME	11D_CB6
EXPNO	2
PROCNO	1

F2 - Acquisition Parameters

Date_	20170610
Time	17.06
INSTRUM	spect
PROBHD	5 mm PABBO BB/
PULPROG	zgpg30
TD	65536
SOLVENT	MeOD
NS	256
DS	4
SWH	29761.904 Hz
FIDRES	0.454131 Hz
AQ	1.1010048 sec
RG	198.57
DW	16.800 usec
DE	6.50 usec
TE	303.0 K
D1	2.00000000 sec
D11	0.03000000 sec
TD0	1

======== CHANNEL f1 ========

SFO1	125.7879670 MHz
NUC1	13C
P1	10.00 usec
PLW1	88.00000000 W

======== CHANNEL f2 ========

SFO2	500.2020008 MHz
NUC2	1H
CPDPRG[2	waltz16
PCPD2	80.00 usec
PLW2	22.00000000 W
PLW12	0.34375000 W
PLW13	0.22000000 W

F2 - Processing parameters

SI	32768
SF	125.7752188 MHz
WDW	EM
SSB	0
LB	1.00 Hz
GB	0
PC	1.40

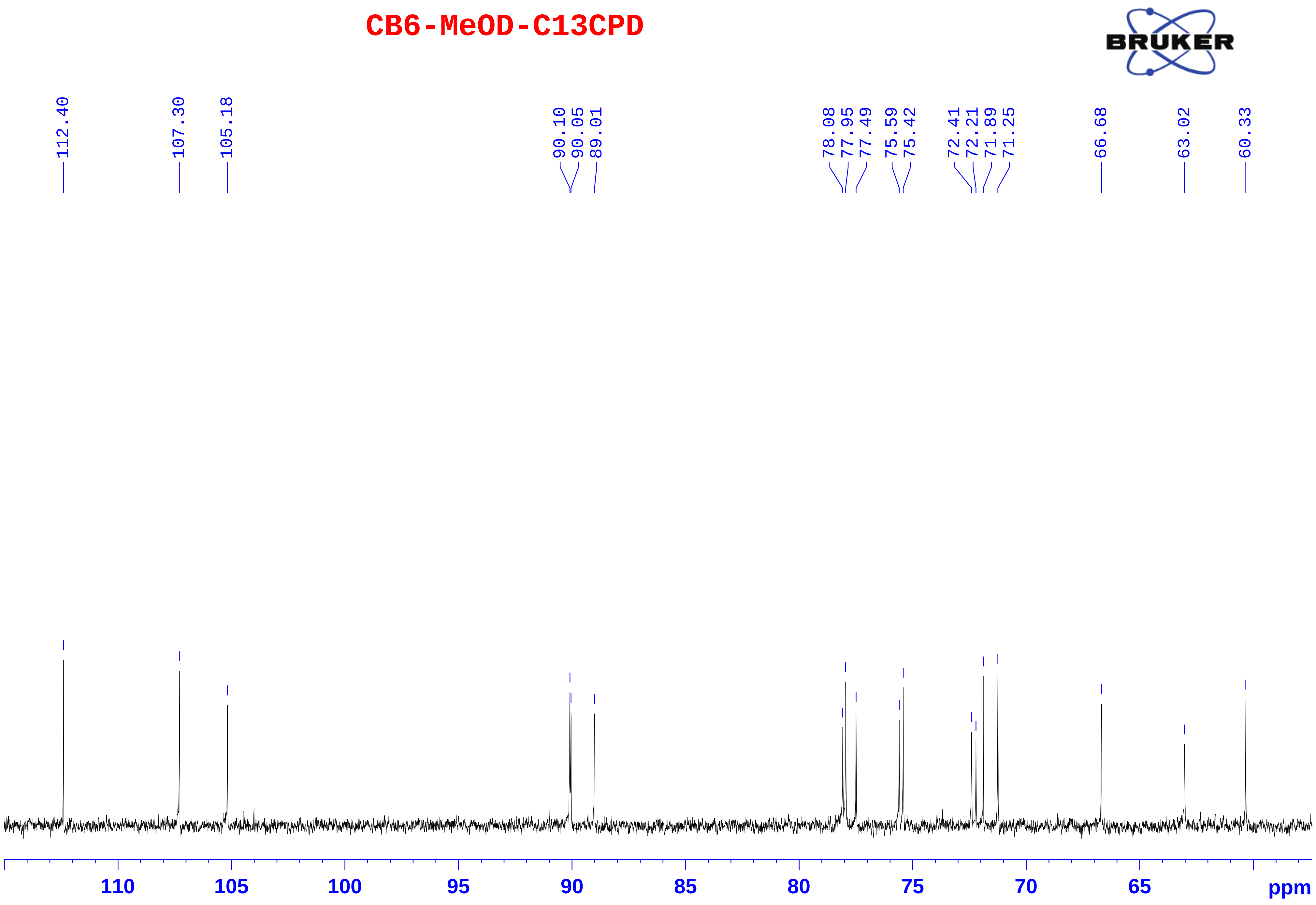
CB6-MeOD-C13CPD
112.40
107.30
105.18
90.10
90.05
89.01
78.08
77.95
77.49
75.59
75.42
72.41
72.21
71.89
71.25
66.68
63.02
60.33
110
105
100
95
90
85
80
75
70
65
ppm

CB6-MeOD-C13CPD

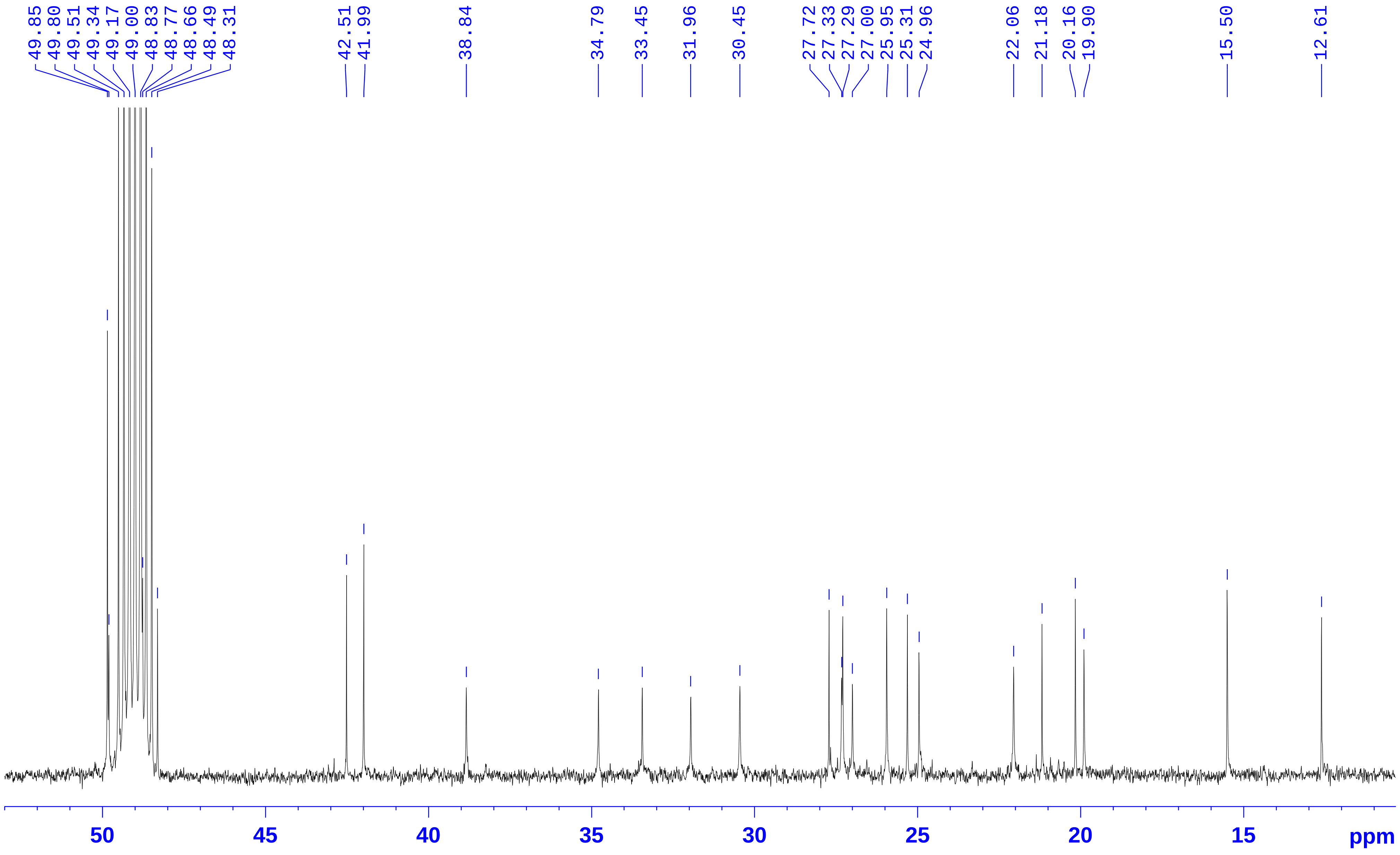

Supplement: Supplementary file 1 [file molecules-23-01083-s001.zip › Supplementary Materials_liping/Figure S14. 13C spectrum of compound 6.pdf]
