# Supplementary material for: Chemical Constituents from Cimicifuga dahurica and Their Anti-Proliferative Effects on MCF-7 Breast Cancer Cells
Source: Molecules. 2018 May 4;23(5):1083. doi: 10.3390/molecules23051083 (PMC6102574; doi:10.3390/molecules23051083)

CB6-MeOD-C13CPD&DEPT

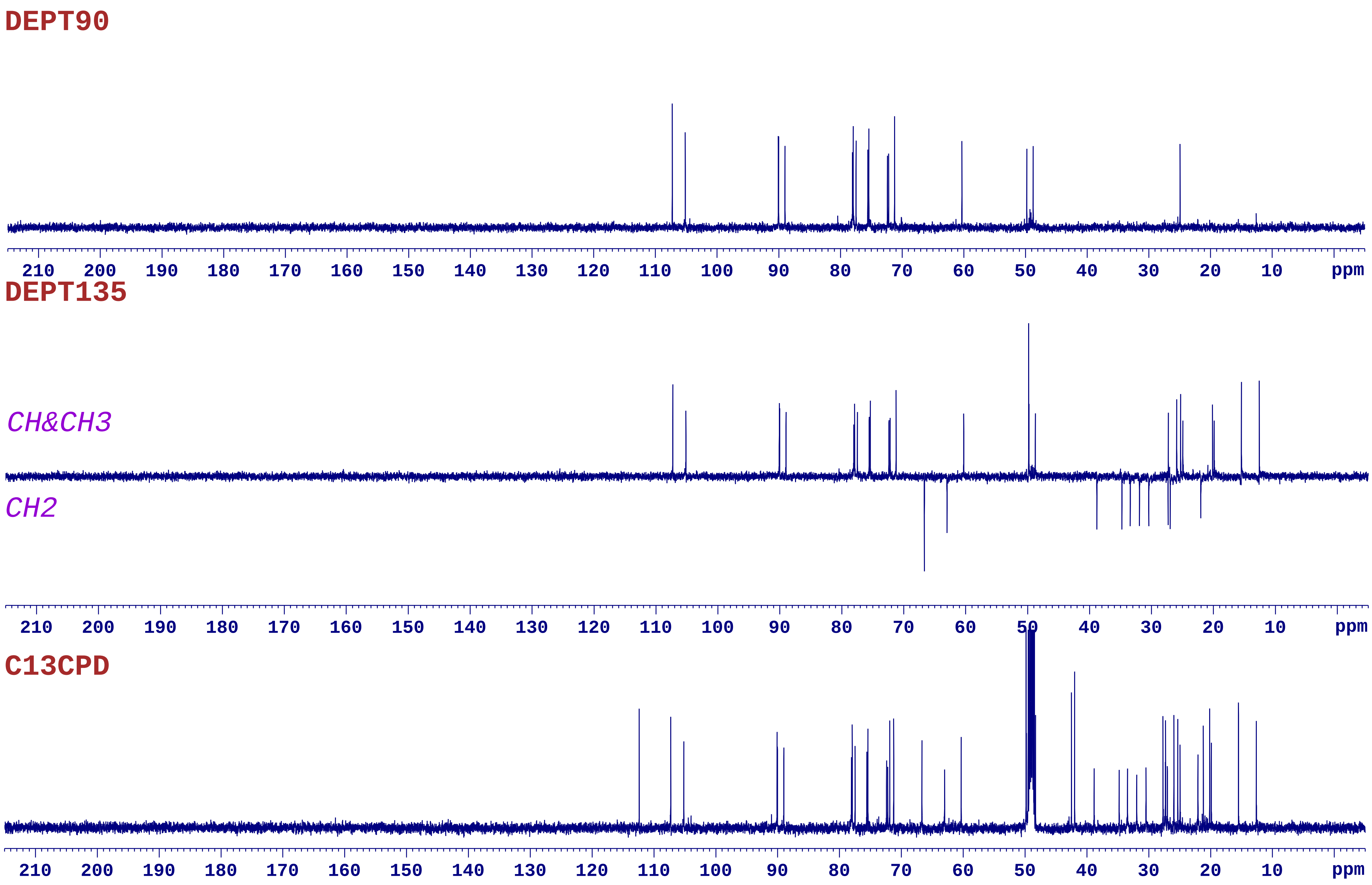

CB6-MeOD-C13CPD&DEPT

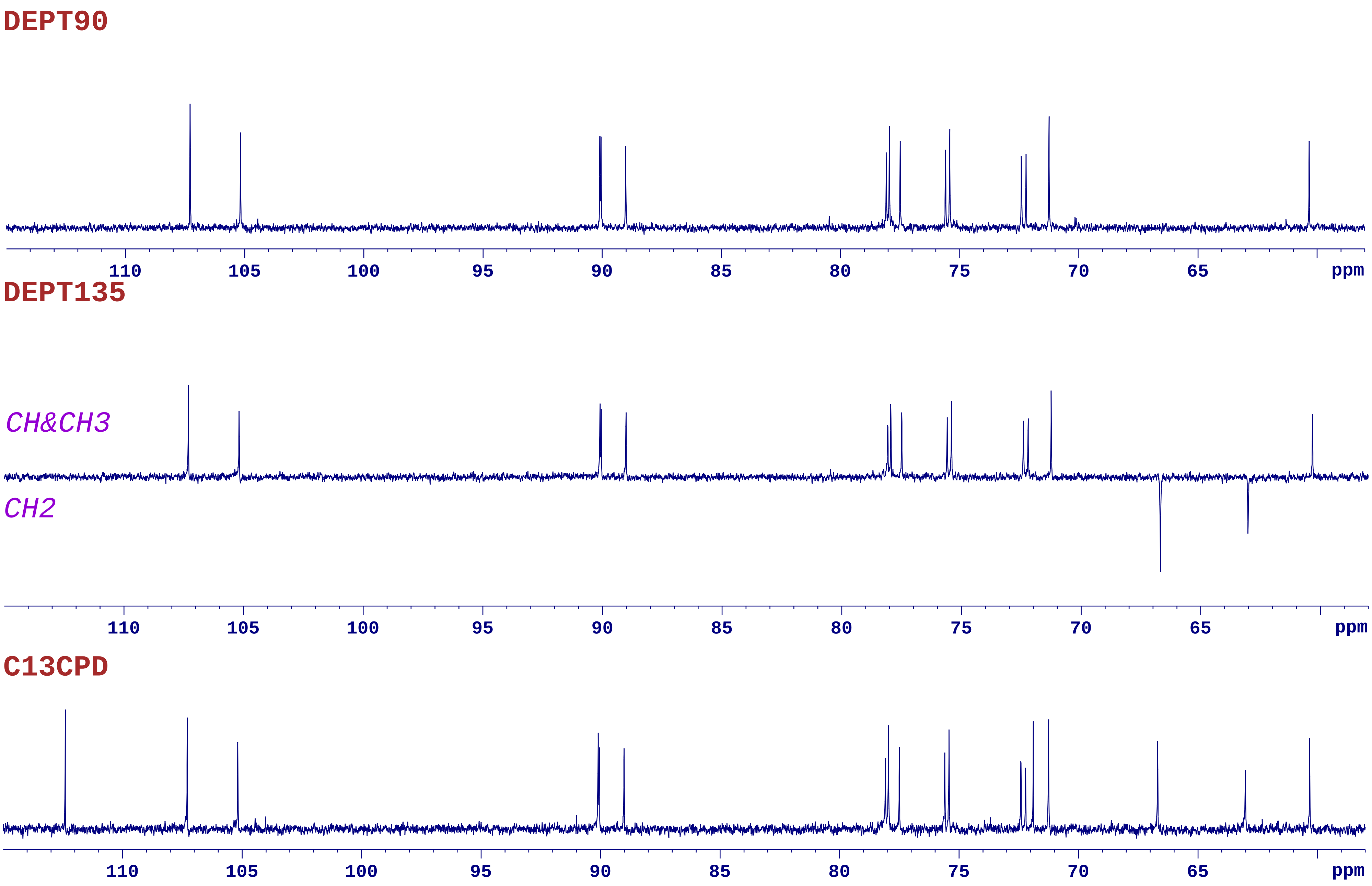

CB6-MeOD-C13CPD&DEPT

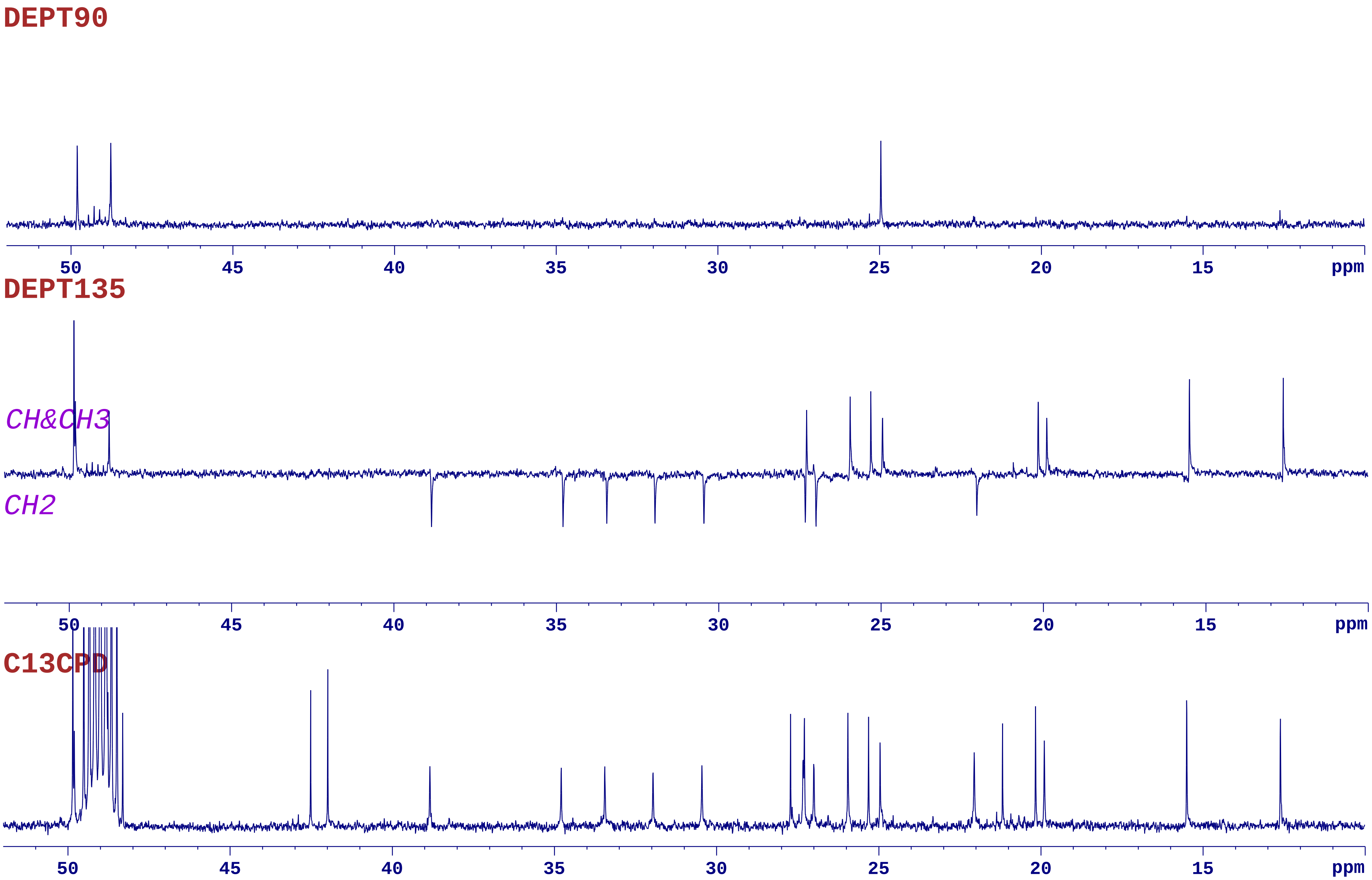

Supplement: Supplementary file 1 [file molecules-23-01083-s001.zip › Supplementary Materials_liping/Figure S15. DEPT spectrum of compound 6.pdf]
