# Supplementary material for: Chemical Constituents from Cimicifuga dahurica and Their Anti-Proliferative Effects on MCF-7 Breast Cancer Cells
Source: Molecules. 2018 May 4;23(5):1083. doi: 10.3390/molecules23051083 (PMC6102574; doi:10.3390/molecules23051083)
Supplement: Supplementary file 1 [file molecules-23-01083-s001.zip › Supplementary Materials_liping/Figure S18. COSY spectrum of compound 6.pdf]

CB6-MeOD-COSYGP

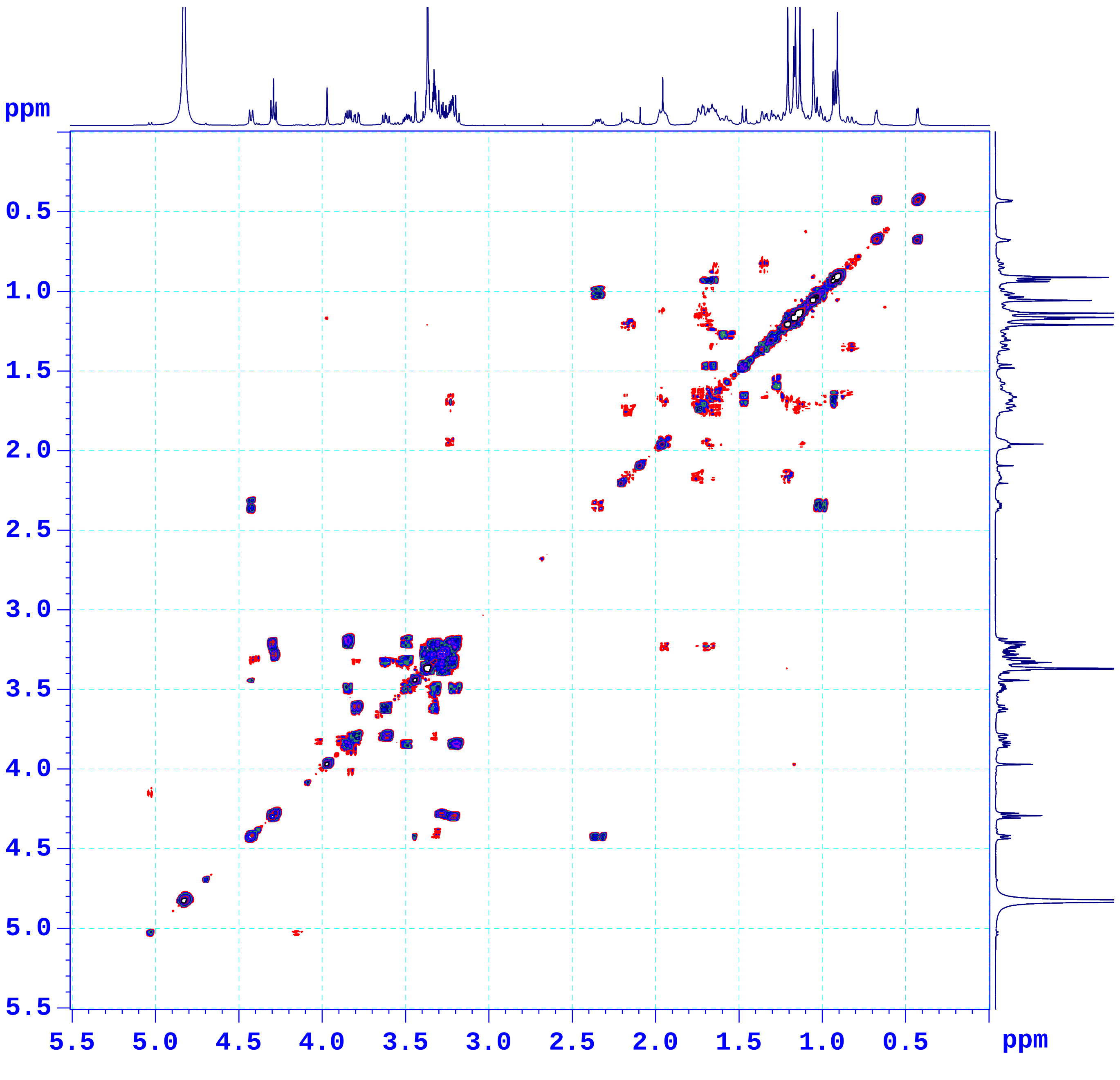


```
Current Data Parameters
NAME              11D_CB6
EXPNO                   7
PROCNO                  1

F2 - Acquisition Parameters
Date_            20170616
Time                20.32
INSTRUM             spect
PROBHD     5 mm PABBO BB/
PULPROG        cosygpppqf
TD                   2048
SOLVENT              MeOD
NS                      2
DS                      8
SWH              2762.431 Hz
FIDRES           1.348843 Hz
AQ              0.3706880 sec
RG                  17.93
DW                181.000 usec
DE                   6.50 usec
TE                  303.0 K
D0             0.00000300 sec
D1             1.78250206 sec
D11            0.03000000 sec
D12            0.00002000 sec
D13            0.00000400 sec
D16            0.00020000 sec
IN0            0.00036200 sec

======== CHANNEL f1 ========
SFO1          500.2013814 MHz
NUC1                   1H
P0                  10.00 usec
P1                  10.00 usec
P17               2500.00 usec
PLW1          22.00000000 W
PLW10          3.25440001 W

====== GRADIENT CHANNEL =====
GPNAM[1]       SMSQ10.100
GPZ1                10.00 %
P16               1000.00 usec

F1 - Acquisition parameters
TD                    128
SFO1             500.2014 MHz
FIDRES          21.581491 Hz
SW                  5.523 ppm
FnMODE                 QF

F2 - Processing parameters
SI                   1024
SF            500.2000054 MHz
WDW                 QSINE
SSB        0
LB         0 Hz
GB         0
PC                   1.40

F1 - Processing parameters
SI                   1024
MC2                    QF
SF            500.2000063 MHz
WDW                 QSINE
SSB        0
LB         0 Hz
GB         0
```

CB6-MeOD-COSYGP

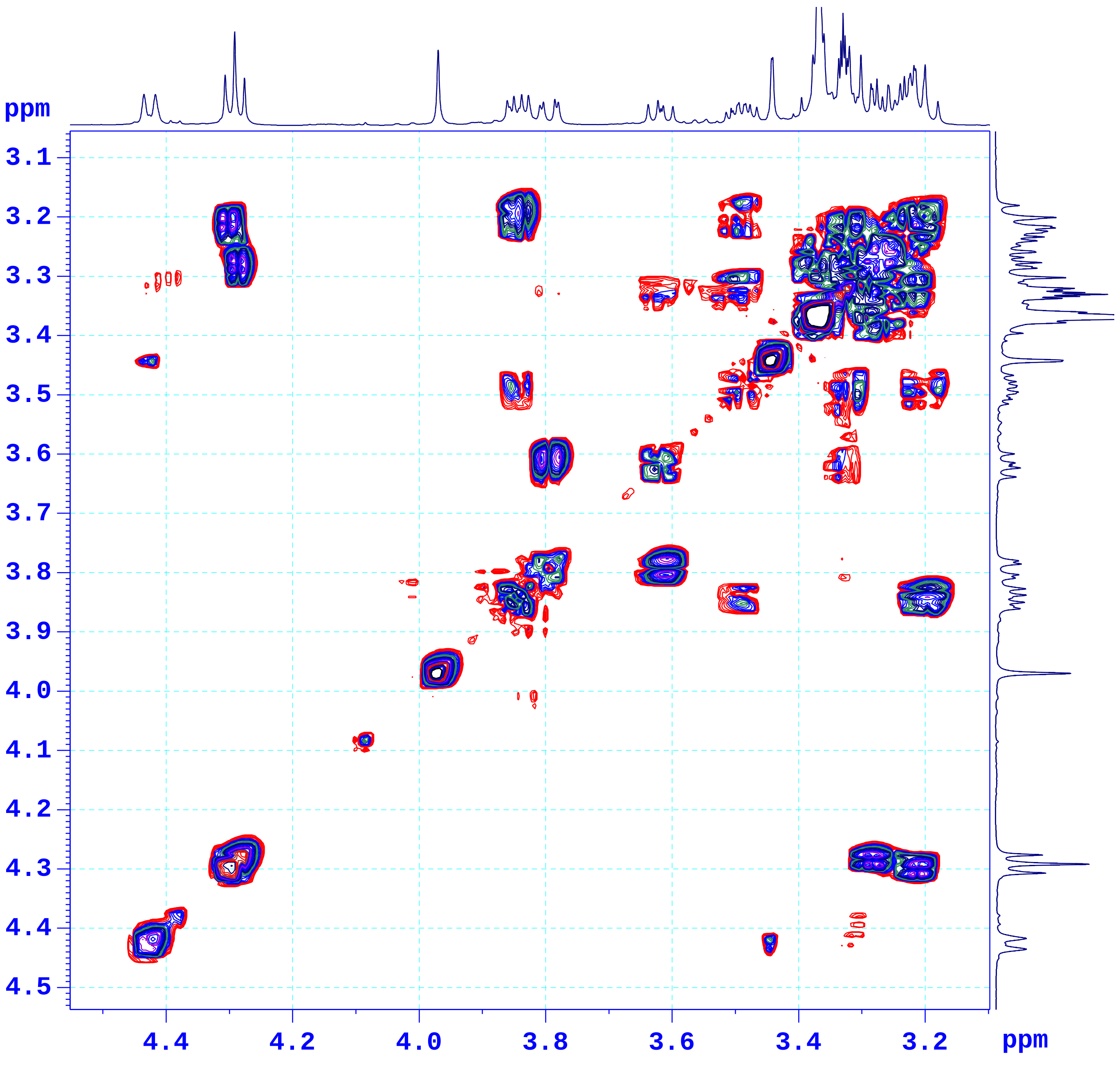


```
Current Data Parameters
NAME                11D_CB6
EXPNO                     7
PROCNO                    1

F2 - Acquisition Parameters
Date_              20170616
Time                  20.32
INSTRUM               spect
PROBHD    5 mm PABBO BB/
PULPROG          cosygpppqf
TD                     2048
SOLVENT                MeOD
NS                        2
DS                        8
SWH              2762.431 Hz
FIDRES           1.348843 Hz
AQ              0.3706880 sec
RG                    17.93
DW                  181.000 usec
DE                    6.50 usec
TE                   303.0 K
D0               0.00000300 sec
D1               1.78250206 sec
D11              0.03000000 sec
D12              0.00002000 sec
D13              0.00000400 sec
D16              0.00020000 sec
IN0              0.00036200 sec

======== CHANNEL f1 ========
SFO1          500.2013814 MHz
NUC1                    1H
P0                  10.00 usec
P1                  10.00 usec
P17               2500.00 usec
PLW1           22.00000000 W
PLW10           3.25440001 W

====== GRADIENT CHANNEL =====
GPNAM[1]      SMSQ10.100
GPZ1                10.00 %
P16               1000.00 usec

F1 - Acquisition parameters
TD                      128
SFO1             500.2014 MHz
FIDRES          21.581491 Hz
SW                   5.523 ppm
FnMODE                   QF

F2 - Processing parameters
SI                     1024
SF            500.2000054 MHz
WDW                   QSINE
SSB          0
LB           0 Hz
GB           0
PC                     1.40

F1 - Processing parameters
SI                     1024
MC2                      QF
SF            500.2000063 MHz
WDW                   QSINE
SSB          0
LB           0 Hz
GB           0
```

CB6-MeOD-COSYGP

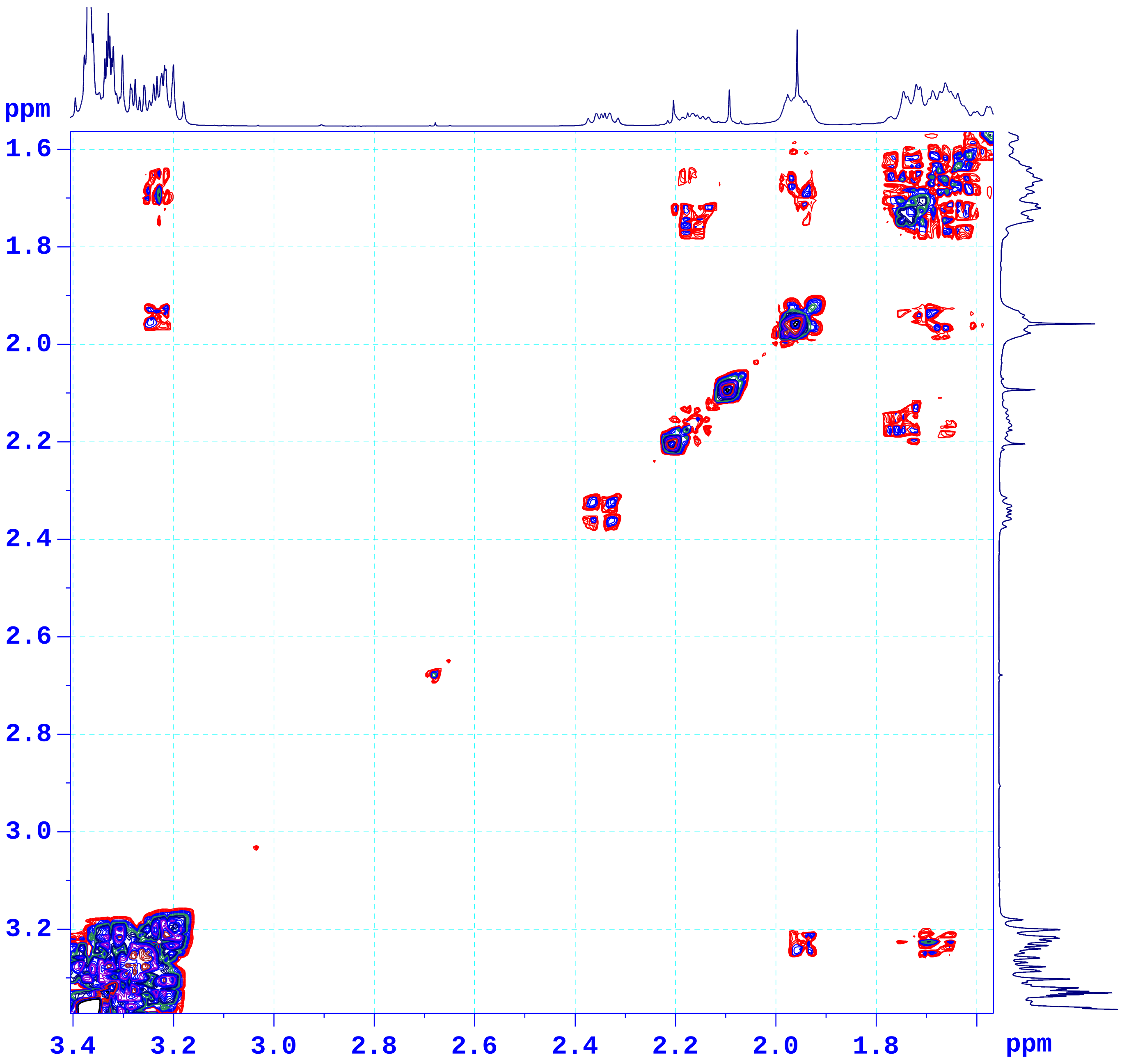

Current Data Parameters
NAME 11D_CB6
EXPNO 7
PROCNO 1

F2 - Acquisition Parameters
Date_ 20170616
Time 20.32
INSTRUM spect
PROBHD 5 mm PABBO BB/
PULPROG cosygpppqf
TD 2048
SOLVENT MeOD
NS 2
DS 8
SWH 2762.431 Hz
FIDRES 1.348843 Hz
AQ 0.3706880 sec
RG 17.93
DW 181.000 usec
DE 6.50 usec
TE 303.0 K
D0 0.00000300 sec
D1 1.78250206 sec
D11 0.03000000 sec
D12 0.00002000 sec
D13 0.00000400 sec
D16 0.00020000 sec
IN0 0.00036200 sec

======== CHANNEL f1 ========
SFO1 500.2013814 MHz
NUC1 1H
P0 10.00 usec
P1 10.00 usec
P17 2500.00 usec
PLW1 22.00000000 W
PLW10 3.25440001 W

====== GRADIENT CHANNEL =====
GPNAM[1] SMSQ10.100
GPZ1 10.00 %
P16 1000.00 usec

F1 - Acquisition parameters
TD 128
SFO1 500.2014 MHz
FIDRES 21.581491 Hz
SW 5.523 ppm
FnMODE QF

F2 - Processing parameters
SI 1024
SF 500.2000054 MHz
WDW QSINE
SSB 0
LB 0 Hz
GB 0
PC 1.40

F1 - Processing parameters
SI 1024
MC2 QF
SF 500.2000063 MHz
WDW QSINE
SSB 0
LB 0 Hz
GB 0

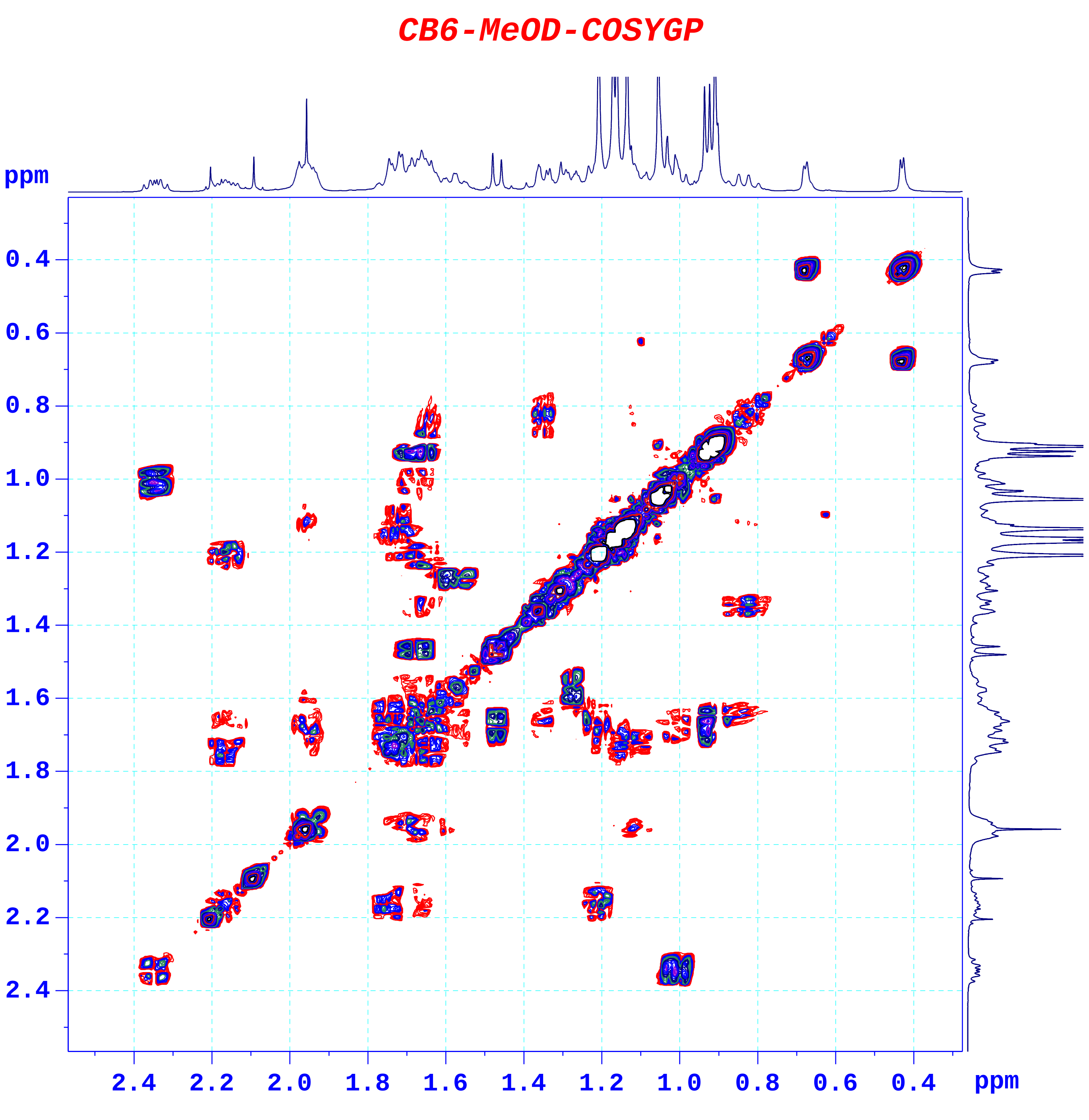

BRUKER

```
Current Data Parameters
NAME                11D_CB6
EXPNO                     7
PROCNO                    1

F2 - Acquisition Parameters
Date_              20170616
Time                  20.32
INSTRUM               spect
PROBHD    5 mm PABBO BB/
PULPROG         cosygpppqf
TD                     2048
SOLVENT                MeOD
NS                        2
DS                        8
SWH                2762.431 Hz
FIDRES             1.348843 Hz
AQ                0.3706880 sec
RG                    17.93
DW                  181.000 usec
DE                     6.50 usec
TE                    303.0 K
D0               0.00000300 sec
D1               1.78250206 sec
D11              0.03000000 sec
D12              0.00002000 sec
D13              0.00000400 sec
D16              0.00020000 sec
IN0              0.00036200 sec

======== CHANNEL f1 ========
SFO1            500.2013814 MHz
NUC1                     1H
P0                    10.00 usec
P1                    10.00 usec
P17                 2500.00 usec
PLW1            22.00000000 W
PLW10            3.25440001 W

====== GRADIENT CHANNEL =====
GPNAM[1]         SMSQ10.100
GPZ1                  10.00 %
P16                 1000.00 usec

F1 - Acquisition parameters
TD                      128
SFO1               500.2014 MHz
FIDRES            21.581491 Hz
SW                    5.523 ppm
FnMODE                   QF

F2 - Processing parameters
SI                     1024
SF              500.2000054 MHz
WDW                   QSINE
SSB      0
LB       0 Hz
GB       0
PC                     1.40

F1 - Processing parameters
SI                     1024
MC2                      QF
SF              500.2000063 MHz
WDW                   QSINE
SSB      0
LB       0 Hz
GB       0
```
