# Supplementary material for: Chemical Constituents from Cimicifuga dahurica and Their Anti-Proliferative Effects on MCF-7 Breast Cancer Cells
Source: Molecules. 2018 May 4;23(5):1083. doi: 10.3390/molecules23051083 (PMC6102574; doi:10.3390/molecules23051083)
Supplement: Supplementary file 1 [file molecules-23-01083-s001.zip › Supplementary Materials_liping/Figure S19. ROESY spectrum of compound 6.pdf]

# CB6-MeOD-ROESY

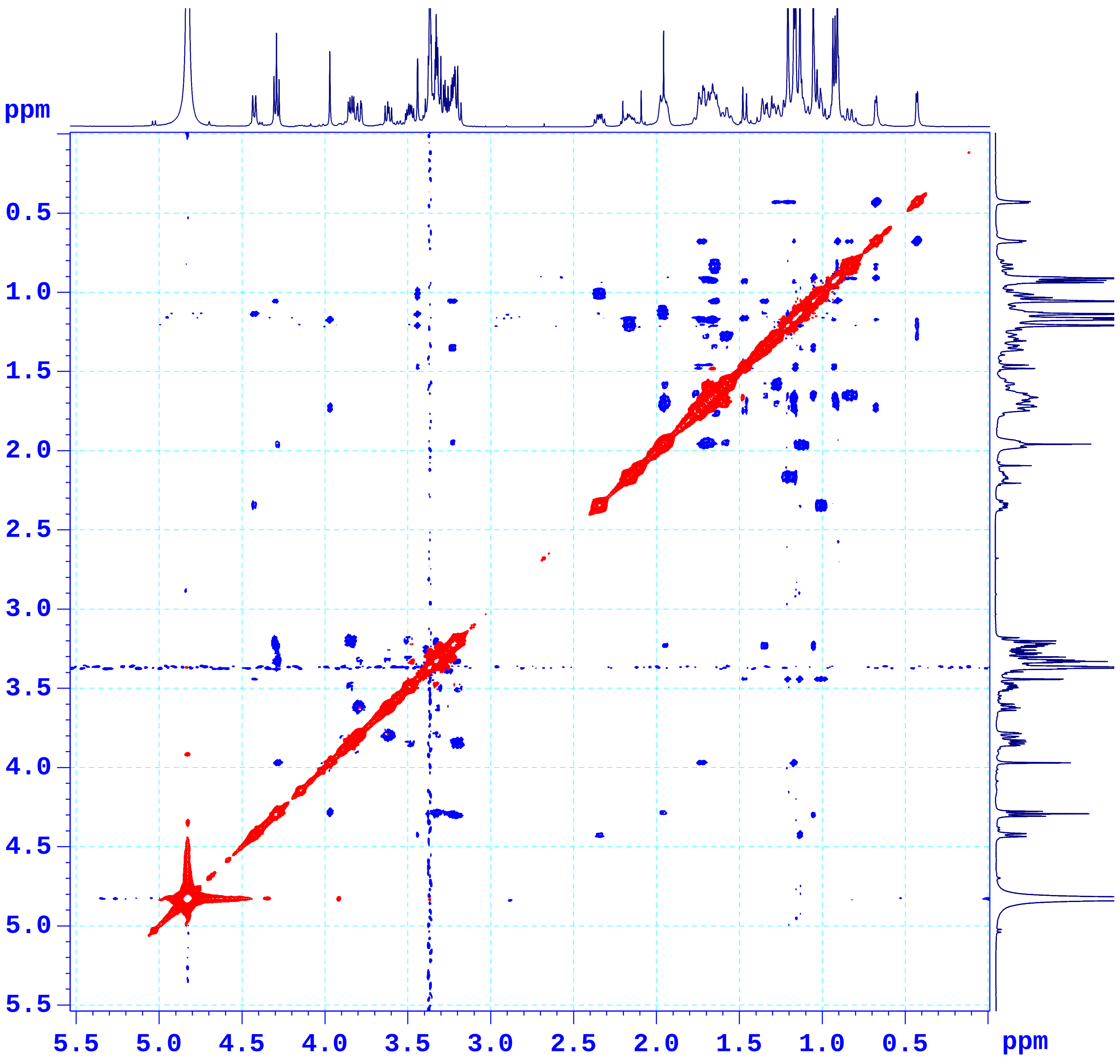


BRUKER

```
Current Data Parameters
NAME              11D_CB6
EXPNO                   8
PROCNO                  1

F2 - Acquisition Parameters
Date_            20170616
Time                20.43
INSTRUM             spect
PROBHD     5 mm PABBO BB/
PULPROG       roesyphpp.2
TD                   2048
SOLVENT              MeOD
NS                      4
DS                     32
SWH              2777.778 Hz
FIDRES           1.356337 Hz
AQ              0.3686400 sec
RG                  17.93
DW                180.000 usec
DE                   6.50 usec
TE                  303.0 K
D0             0.00016963 sec
D1             1.83615994 sec
D11            0.03000000 sec
D12            0.00002000 sec
IN0            0.00036000 sec
L4                   1000
P15             200000.00 usec

======== CHANNEL f1 ========
SFO1          500.2013822 MHz
NUC1                   1H
P1                  10.00 usec
P17               2500.00 usec
P25                100.00 usec
PLW1          22.00000000 W
PLW10          3.25440001 W
PLW27          0.88000000 W

F1 - Acquisition parameters
TD                    256
SFO1             500.2014 MHz
FIDRES          10.850695 Hz
SW                  5.553 ppm
FnMODE        States-TPPI

F2 - Processing parameters
SI                   1024
SF            500.2000000 MHz
WDW                 QSINE
SSB                     2
LB          0 Hz
GB          0
PC                   1.00

F1 - Processing parameters
SI                   1024
MC2           States-TPPI
SF            500.2000000 MHz
WDW                 QSINE
SSB                     2
LB          0 Hz
GB          0
```

## CB6-MeOD-ROESY

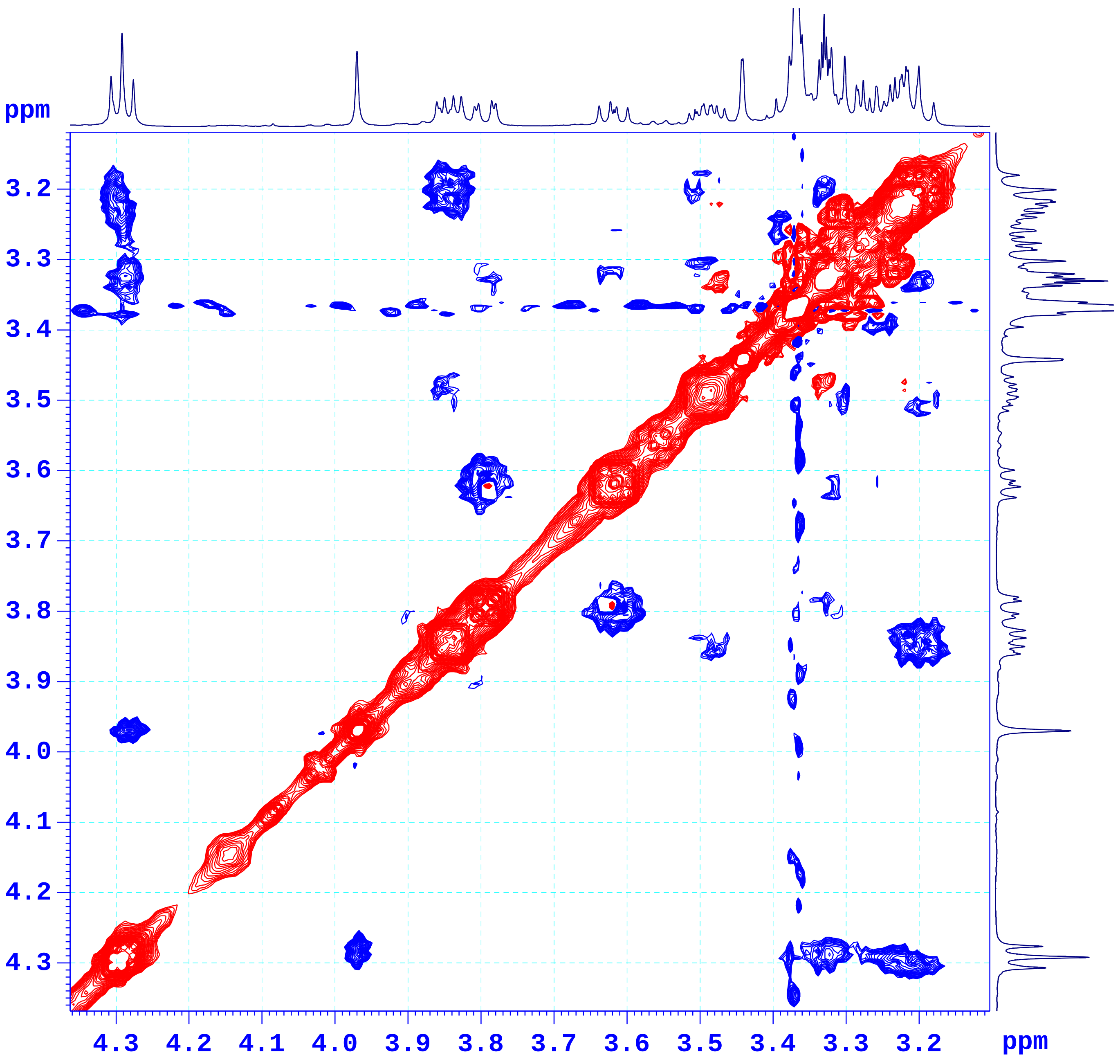


Current Data Parameters
NAME 11D_CB6
EXPNO 8
PROCNO 1

F2 - Acquisition Parameters
Date_ 20170616
Time 20.43
INSTRUM spect
PROBHD 5 mm PABBO BB/
PULPROG roesyphpp.2
TD 2048
SOLVENT MeOD
NS 4
DS 32
SWH 2777.778 Hz
FIDRES 1.356337 Hz
AQ 0.3686400 sec
RG 17.93
DW 180.000 usec
DE 6.50 usec
TE 303.0 K
D0 0.00016963 sec
D1 1.83615994 sec
D11 0.03000000 sec
D12 0.00002000 sec
IN0 0.00036000 sec
L4 1000
P15 200000.00 usec

======== CHANNEL f1 ========
SFO1 500.2013822 MHz
NUC1 1H
P1 10.00 usec
P17 2500.00 usec
P25 100.00 usec
PLW1 22.00000000 W
PLW10 3.25440001 W
PLW27 0.88000000 W

F1 - Acquisition parameters
TD 256
SFO1 500.2014 MHz
FIDRES 10.850695 Hz
SW 5.553 ppm
FnMODE States-TPPI

F2 - Processing parameters
SI 1024
SF 500.2000000 MHz
WDW QSINE
SSB 2
LB 0 Hz
GB 0
PC 1.00

F1 - Processing parameters
SI 1024
MC2 States-TPPI
SF 500.2000000 MHz
WDW QSINE
SSB 2
LB 0 Hz
GB 0

## CB6-MeOD-ROESY

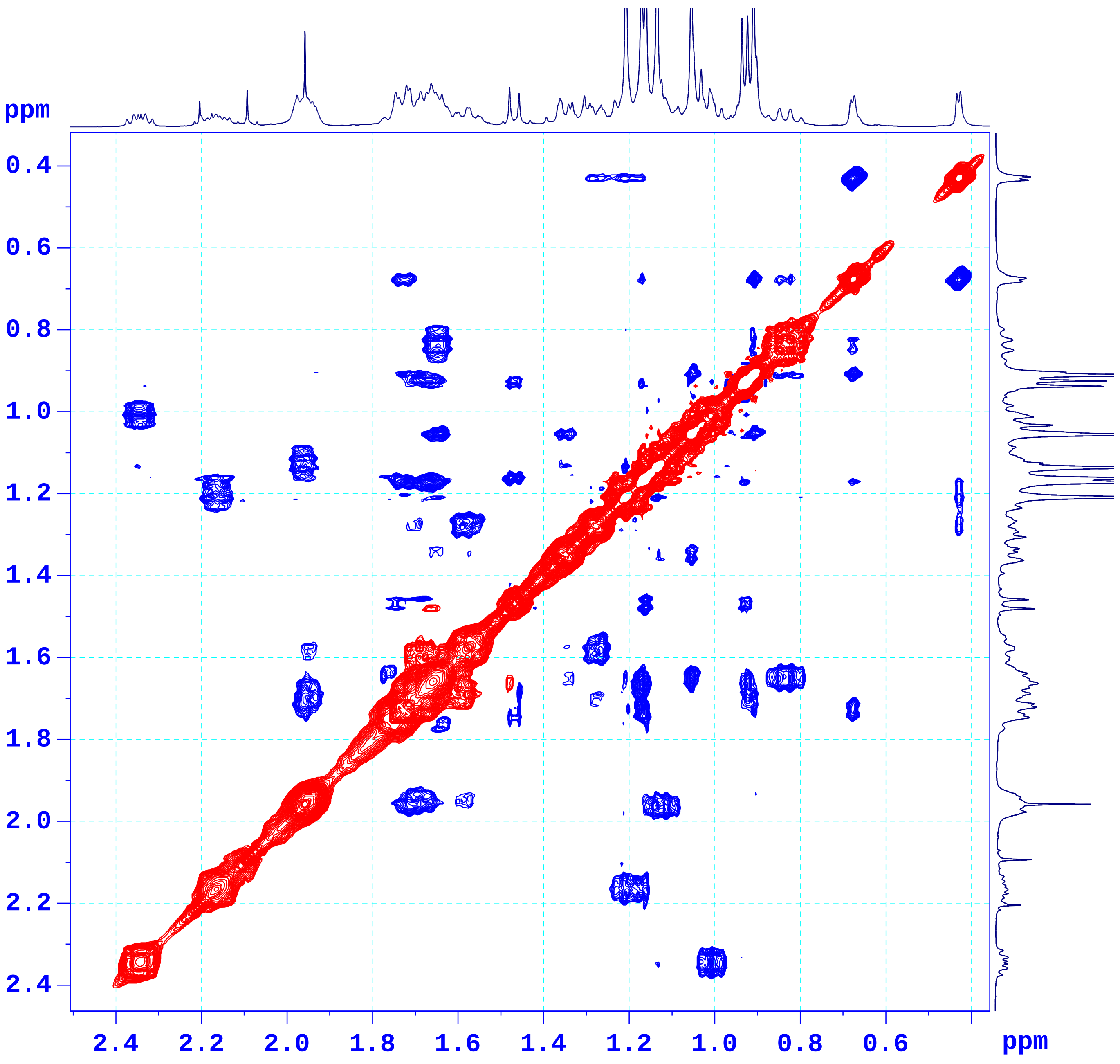


```
Current Data Parameters
NAME                11D_CB6
EXPNO                     8
PROCNO                    1

F2 - Acquisition Parameters
Date_              20170616
Time                  20.43
INSTRUM               spect
PROBHD   5 mm PABBO BB/
PULPROG        roesyphpp.2
TD                     2048
SOLVENT                MeOD
NS                        4
DS                       32
SWH                2777.778 Hz
FIDRES             1.356337 Hz
AQ                0.3686400 sec
RG                    17.93
DW                  180.000 usec
DE                     6.50 usec
TE                    303.0 K
D0               0.00016963 sec
D1               1.83615994 sec
D11              0.03000000 sec
D12              0.00002000 sec
IN0              0.00036000 sec
L4                     1000
P15               200000.00 usec

======== CHANNEL f1 ========
SFO1            500.2013822 MHz
NUC1                     1H
P1                    10.00 usec
P17                 2500.00 usec
P25                  100.00 usec
PLW1            22.00000000 W
PLW10            3.25440001 W
PLW27            0.88000000 W

F1 - Acquisition parameters
TD                      256
SFO1               500.2014 MHz
FIDRES            10.850695 Hz
SW                    5.553 ppm
FnMODE          States-TPPI

F2 - Processing parameters
SI                     1024
SF              500.2000000 MHz
WDW                   QSINE
SSB                       2
LB           0 Hz
GB           0
PC                     1.00

F1 - Processing parameters
SI                     1024
MC2             States-TPPI
SF              500.2000000 MHz
WDW                   QSINE
SSB                       2
LB           0 Hz
GB           0
```

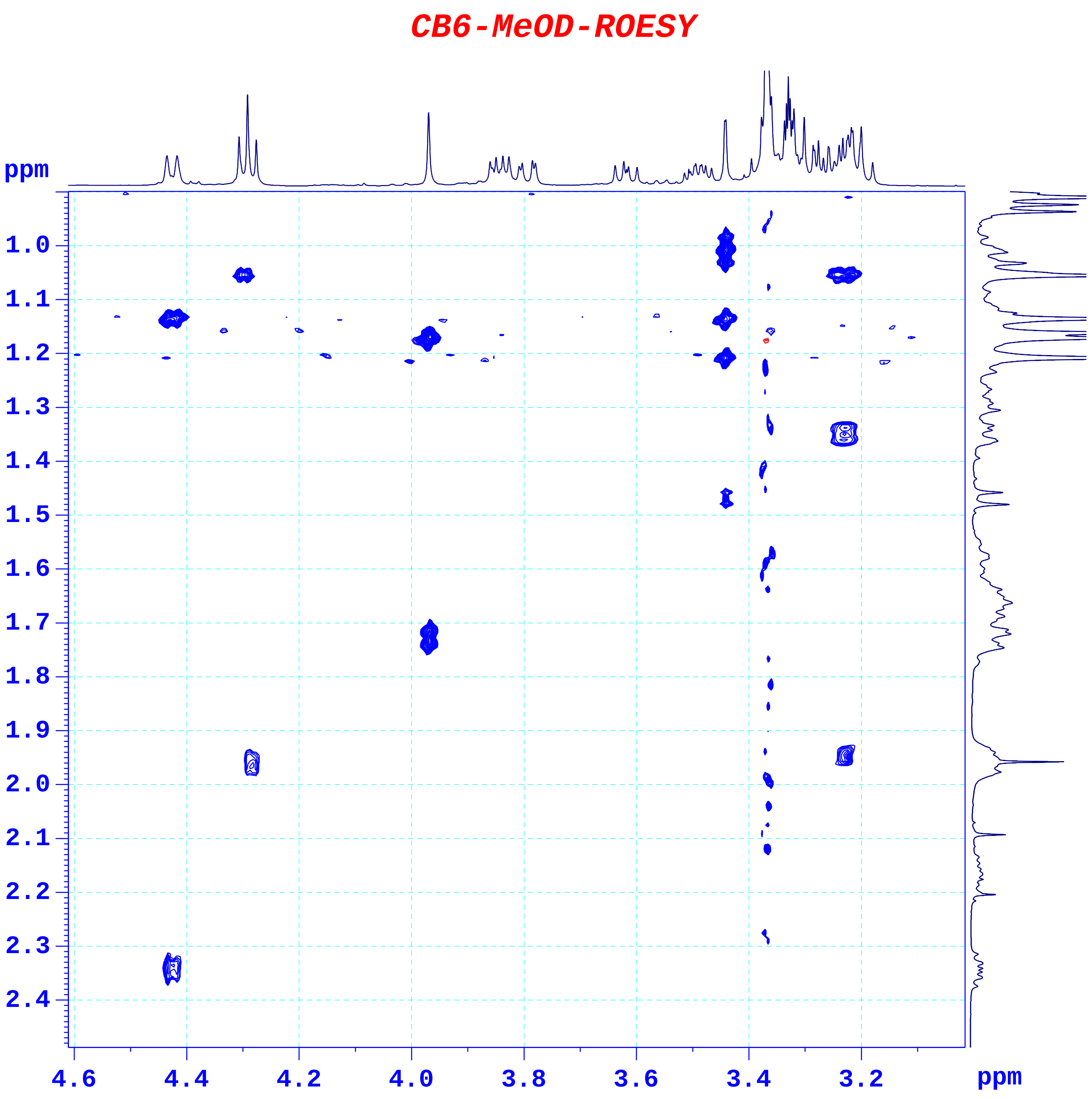


Current Data Parameters
NAME 11D_CB6
EXPNO 8
PROCNO 1

F2 - Acquisition Parameters
Date_ 20170616
Time 20.43
INSTRUM spect
PROBHD 5 mm PABBO BB/
PULPROG roesyphpp.2
TD 2048
SOLVENT MeOD
NS 4
DS 32
SWH 2777.778 Hz
FIDRES 1.356337 Hz
AQ 0.3686400 sec
RG 17.93
DW 180.000 usec
DE 6.50 usec
TE 303.0 K
D0 0.00016963 sec
D1 1.83615994 sec
D11 0.03000000 sec
D12 0.00002000 sec
IN0 0.00036000 sec
L4 1000
P15 200000.00 usec

======== CHANNEL f1 ========
SFO1 500.2013822 MHz
NUC1 1H
P1 10.00 usec
P17 2500.00 usec
P25 100.00 usec
PLW1 22.00000000 W
PLW10 3.25440001 W
PLW27 0.88000000 W

F1 - Acquisition parameters
TD 256
SFO1 500.2014 MHz
FIDRES 10.850695 Hz
SW 5.553 ppm
FnMODE States-TPPI

F2 - Processing parameters
SI 1024
SF 500.2000000 MHz
WDW QSINE
SSB 2
LB 0 Hz
GB 0
PC 1.00

F1 - Processing parameters
SI 1024
MC2 States-TPPI
SF 500.2000000 MHz
WDW QSINE
SSB 2
LB 0 Hz
GB 0
