# Supplementary material for: Chemical Constituents from Cimicifuga dahurica and Their Anti-Proliferative Effects on MCF-7 Breast Cancer Cells
Source: Molecules. 2018 May 4;23(5):1083. doi: 10.3390/molecules23051083 (PMC6102574; doi:10.3390/molecules23051083)
Supplement: Supplementary file 1 [file molecules-23-01083-s001.zip › Supplementary Materials_liping/Figure S20. 1H spectrum of compound 7.pdf]

CB7-MeOD-1H

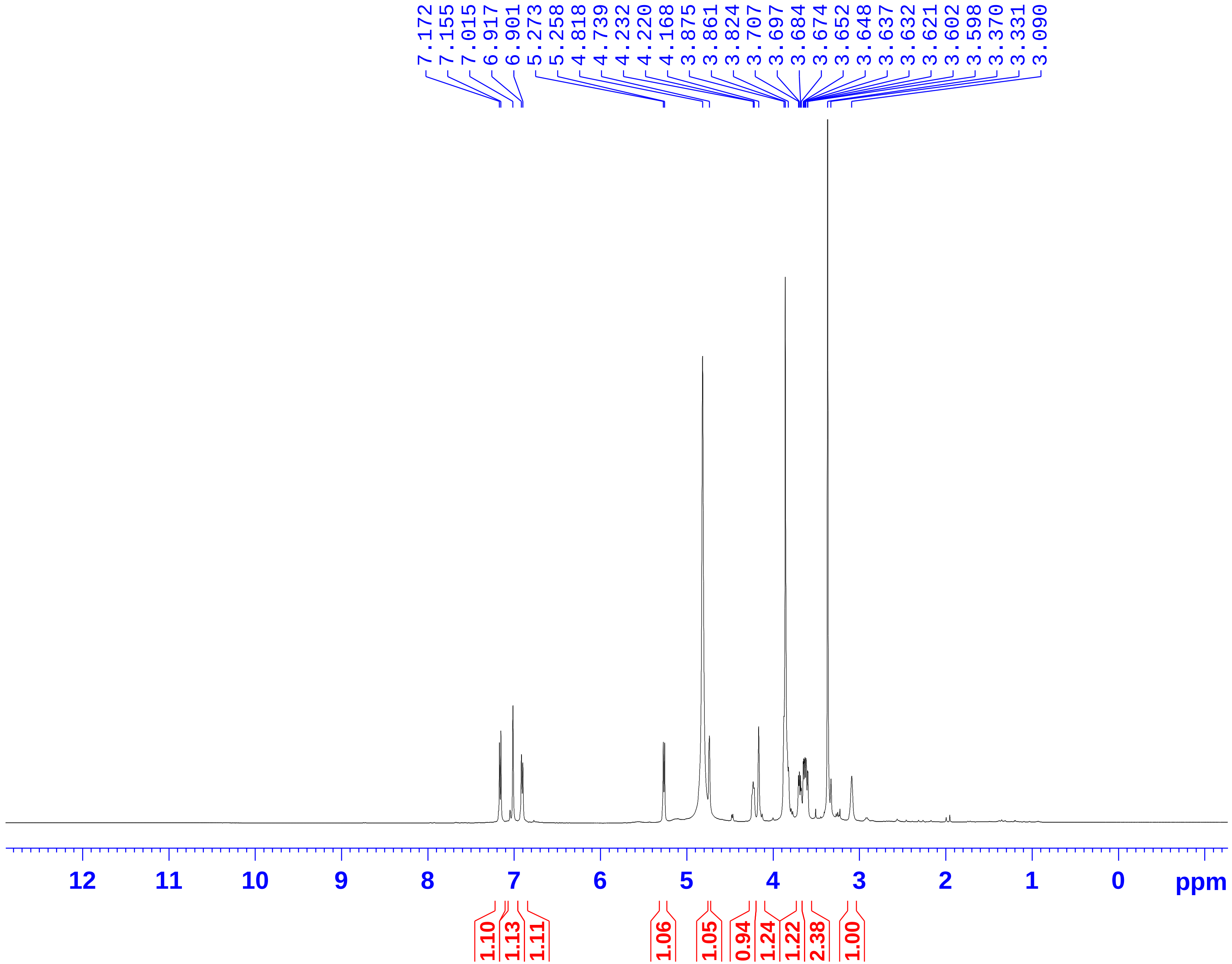

BRUKER

Current Data Parameters
NAME 11D_CB7
EXPNO 1
PROCNO 1

F2 - Acquisition Parameters
Date_ 20170623
Time 16.22
INSTRUM spect
PROBHD 5 mm PABBO BB/
PULPROG zg30
TD 65536
SOLVENT MeOD
NS 16
DS 2
SWH 10000.000 Hz
FIDRES 0.152588 Hz
AQ 3.2767999 sec
RG 22.03
DW 50.000 usec
DE 6.50 usec
TE 302.4 K
D1 1.00000000 sec
TD0 1

======== CHANNEL f1 ========
SFO1 500.2030889 MHz
NUC1 1H
P1 10.00 usec
PLW1 22.00000000 W

F2 - Processing parameters
SI 65536
SF 500.2000000 MHz
WDW EM
SSB 0
LB 0.30 Hz
GB 0
PC 1.00

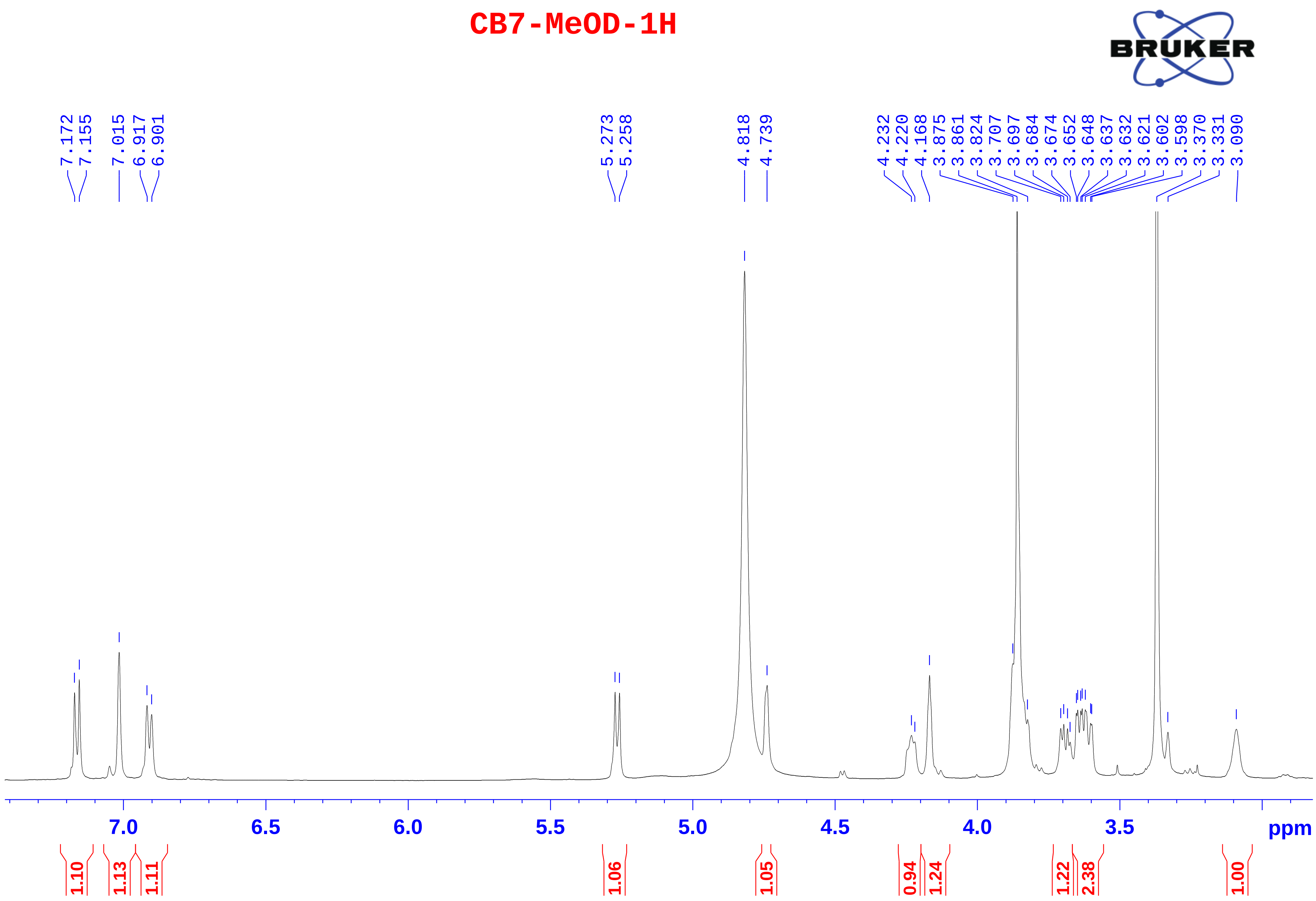
CB7-MeOD-1H
7.172
7.155
7.015
6.917
6.901
5.273
5.258
4.818
4.739
4.232
4.220
4.168
3.875
3.861
3.824
3.707
3.697
3.684
3.674
3.652
3.648
3.637
3.632
3.621
3.602
3.598
3.370
3.331
3.090
7.0
6.5
6.0
5.5
5.0
4.5
4.0
3.5
ppm
1.10
1.13
1.11
1.06
1.05
0.94
1.24
1.22
2.38
1.00
