# Supplementary material for: Chemical Constituents from Cimicifuga dahurica and Their Anti-Proliferative Effects on MCF-7 Breast Cancer Cells
Source: Molecules. 2018 May 4;23(5):1083. doi: 10.3390/molecules23051083 (PMC6102574; doi:10.3390/molecules23051083)
Supplement: Supplementary file 1 [file molecules-23-01083-s001.zip › Supplementary Materials_liping/Figure S21. 13C spectrum of compound 7.pdf]

CB7-MeOD-C13CPD

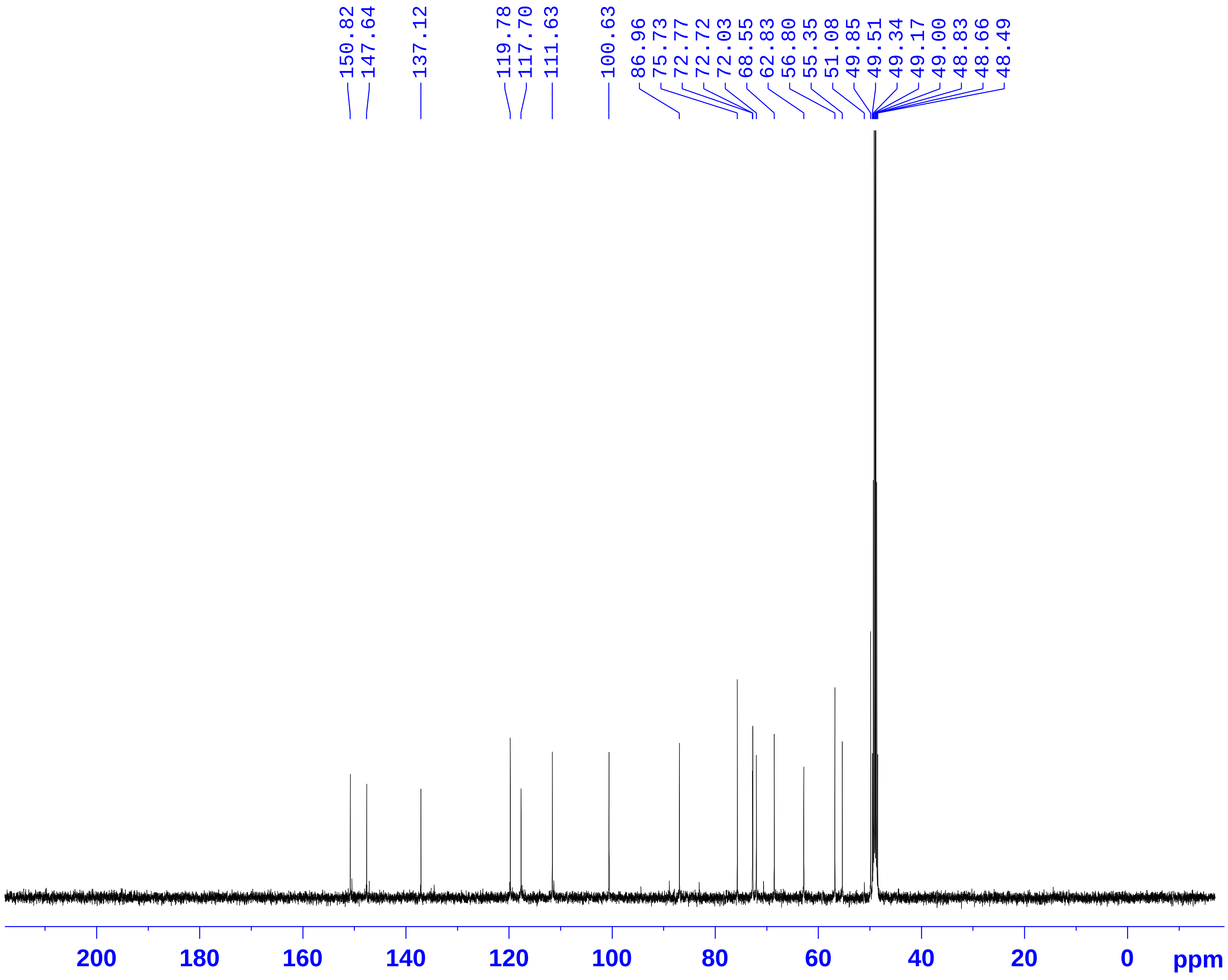


```
Current Data Parameters
NAME              11D_CB7
EXPNO                   2
PROCNO                  1

F2 - Acquisition Parameters
Date_            20170624
Time                12.55
INSTRUM             spect
PROBHD   5 mm PABBO BB/
PULPROG           zgpg30
TD                  65536
SOLVENT              MeOD
NS                     64
DS                      4
SWH             29761.904 Hz
FIDRES           0.454131 Hz
AQ              1.1010048 sec
RG                 198.57
DW                 16.800 usec
DE                   6.50 usec
TE                  303.5 K
D1             2.00000000 sec
D11            0.03000000 sec
TD0                     1

======== CHANNEL f1 ========
SFO1          125.7879670 MHz
NUC1                  13C
P1                  10.00 usec
PLW1          88.00000000 W

======== CHANNEL f2 ========
SFO2          500.2020008 MHz
NUC2                   1H
CPDPRG[2          waltz16
PCPD2               80.00 usec
PLW2          22.00000000 W
PLW12          0.34375000 W
PLW13          0.22000000 W

F2 - Processing parameters
SI                  32768
SF            125.7752268 MHz
WDW                    EM
SSB      0
LB                   1.00 Hz
GB       0
PC                   1.40
```

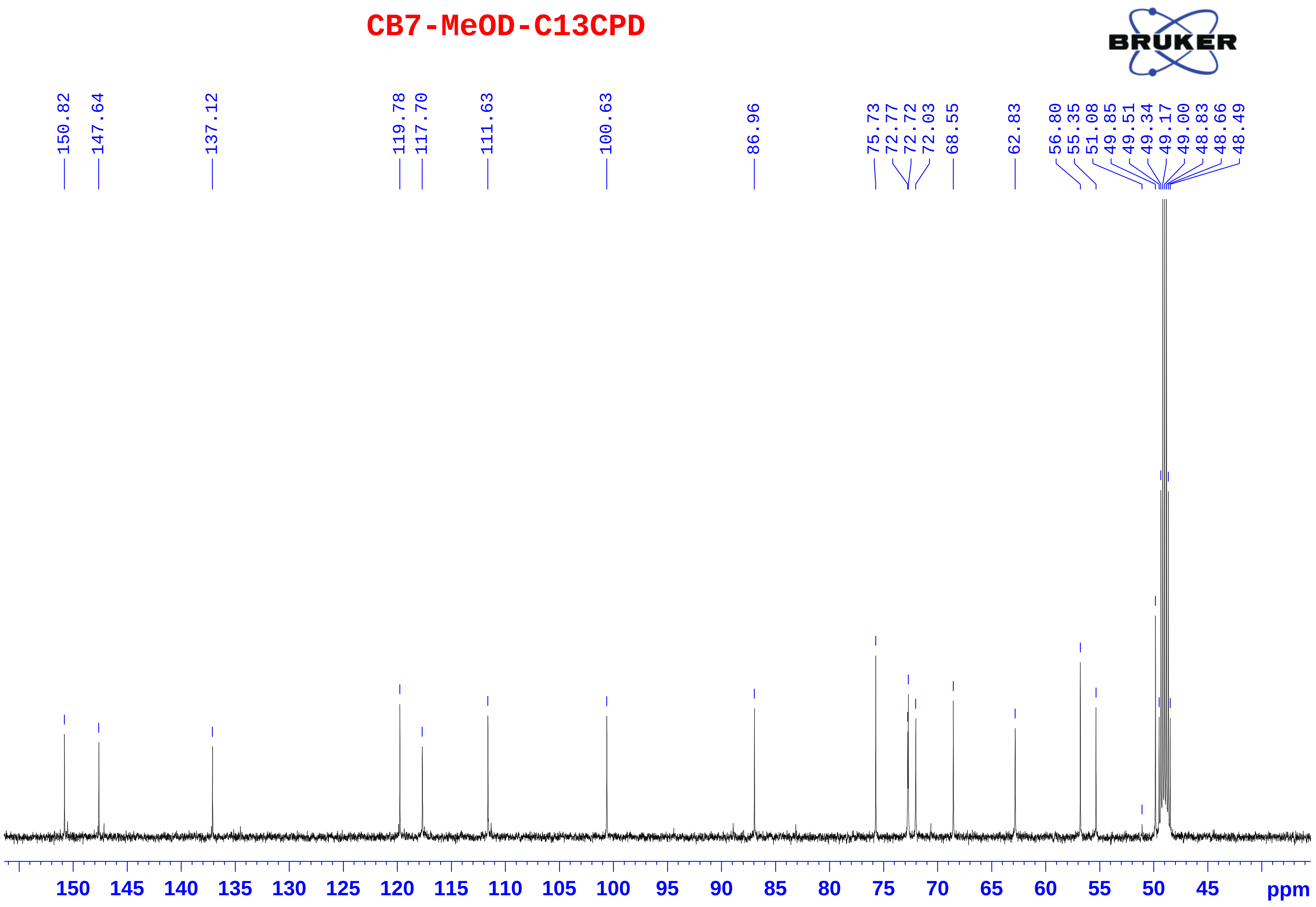

CB7-MeOD-C13CPD
150.82
147.64
137.12
119.78
117.70
111.63
100.63
86.96
75.73
72.77
72.72
72.03
68.55
62.83
56.80
55.35
51.08
49.85
49.51
49.34
49.17
49.00
48.83
48.66
48.49
150 145 140 135 130 125 120 115 110 105 100 95 90 85 80 75 70 65 60 55 50 45
ppm
