# Supplementary material for: Chemical Constituents from Cimicifuga dahurica and Their Anti-Proliferative Effects on MCF-7 Breast Cancer Cells
Source: Molecules. 2018 May 4;23(5):1083. doi: 10.3390/molecules23051083 (PMC6102574; doi:10.3390/molecules23051083)
Supplement: Supplementary file 1 [file molecules-23-01083-s001.zip › Supplementary Materials_liping/Figure S25. COSY spectrum of compound 7.pdf]

CB7-MeOD-COSYGP

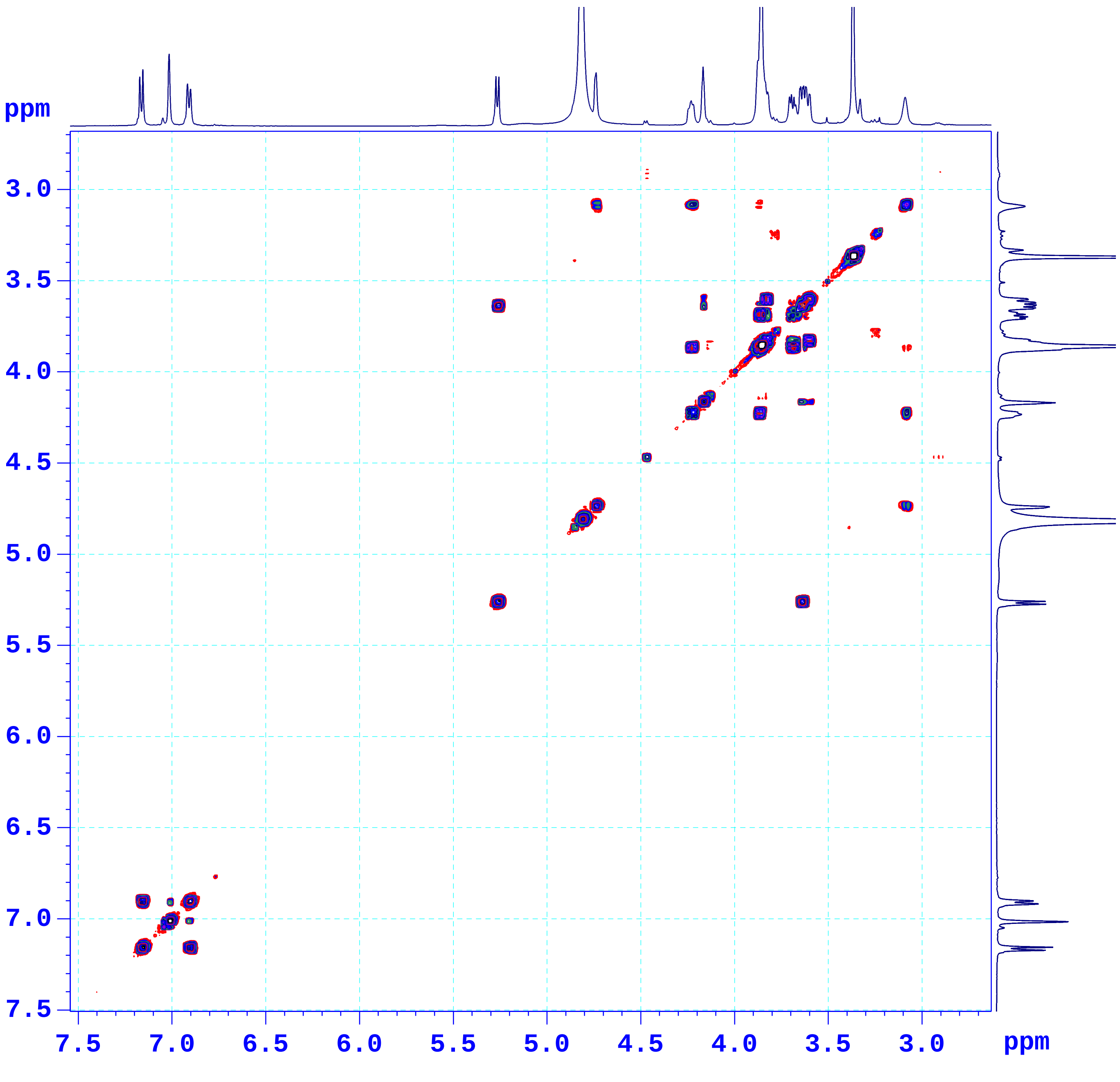

BRUKER

Current Data Parameters

NAME	11D_CB7
EXPNO	7
PROCNO	1

F2 - Acquisition Parameters

Date_	20170703
Time	5.47
INSTRUM	spect
PROBHD	5 mm PABBO BB/
PULPROG	cosygpppqf
TD	2048
SOLVENT	MeOD
NS	2
DS	8
SWH	3496.503 Hz
FIDRES	1.707277 Hz
AQ	0.2928640 sec
RG	14.29
DW	143.000 usec
DE	6.50 usec
TE	303.0 K
D0	0.00000300 sec
D1	1.86032605 sec
D11	0.03000000 sec
D12	0.00002000 sec
D13	0.00000400 sec
D16	0.00020000 sec
IN0	0.00028600 sec

======== CHANNEL f1 ========

SFO1	500.2024863 MHz
NUC1	1H
P0	10.00 usec
P1	10.00 usec
P17	2500.00 usec
PLW1	22.00000000 W
PLW10	3.25440001 W

====== GRADIENT CHANNEL =====

GPNAM[1]	SMSQ10.100
GPZ1	10.00 %
P16	1000.00 usec

F1 - Acquisition parameters

TD	128
SFO1	500.2025 MHz
FIDRES	27.316433 Hz
SW	6.990 ppm
FnMODE	QF

F2 - Processing parameters

SI	1024
SF	500.2000000 MHz
WDW	QSINE
SSB	0
LB	0 Hz
GB	0
PC	1.40

F1 - Processing parameters

SI	1024
MC2	QF
SF	500.2000000 MHz
WDW	QSINE
SSB	0
LB	0 Hz
GB	0

CB7-MeOD-COSYGP

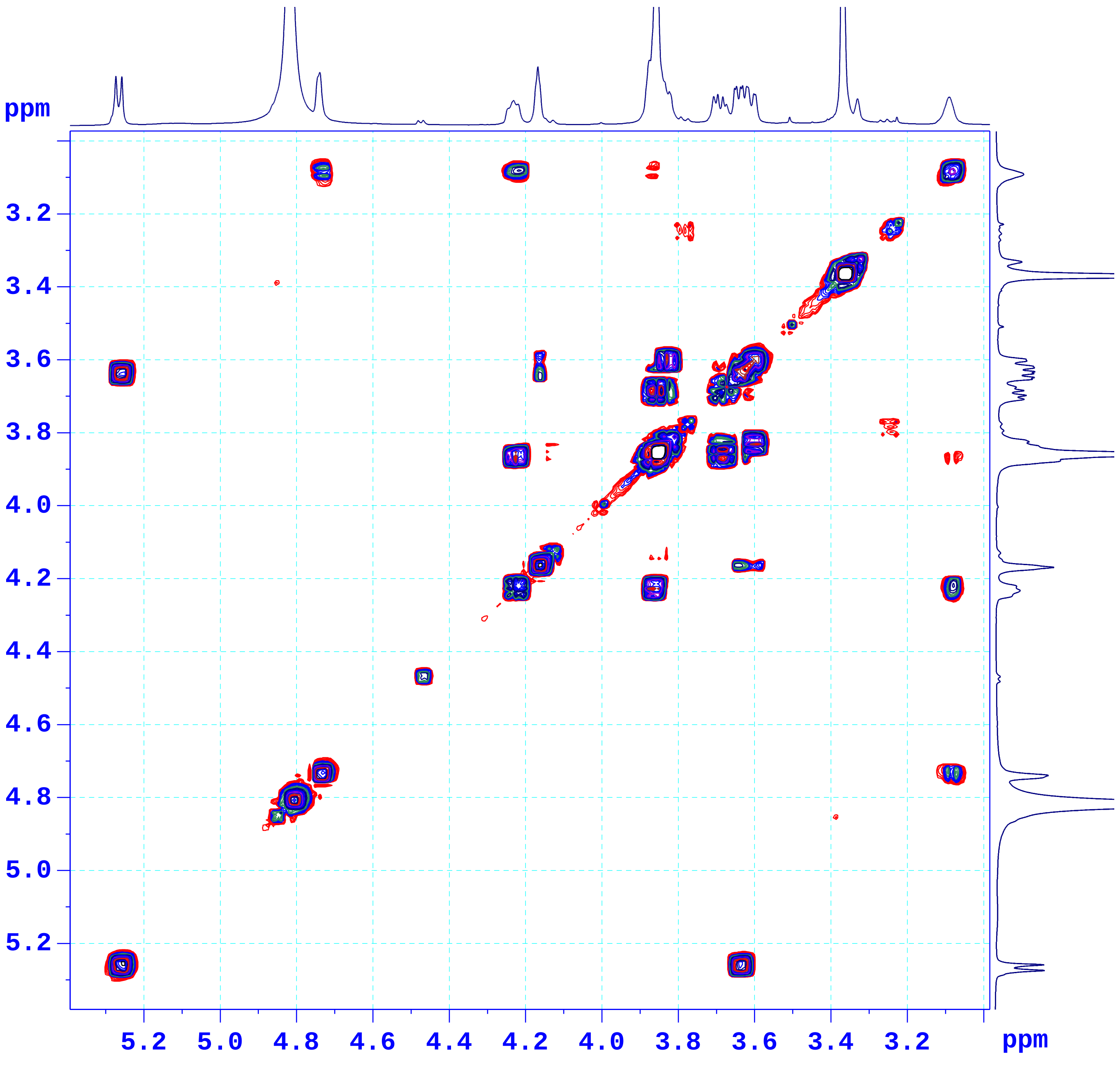

Current Data Parameters

NAME	11D_CB7
EXPNO	7
PROCNO	1

F2 - Acquisition Parameters

Date_	20170703
Time	5.47
INSTRUM	spect
PROBHD	5 mm PABBO BB/
PULPROG	cosygpppqf
TD	2048
SOLVENT	MeOD
NS	2
DS	8
SWH	3496.503 Hz
FIDRES	1.707277 Hz
AQ	0.2928640 sec
RG	14.29
DW	143.000 usec
DE	6.50 usec
TE	303.0 K
D0	0.00000300 sec
D1	1.86032605 sec
D11	0.03000000 sec
D12	0.00002000 sec
D13	0.00000400 sec
D16	0.00020000 sec
IN0	0.00028600 sec

======== CHANNEL f1 ========

SFO1	500.2024863 MHz
NUC1	1H
P0	10.00 usec
P1	10.00 usec
P17	2500.00 usec
PLW1	22.00000000 W
PLW10	3.25440001 W

====== GRADIENT CHANNEL =====

GPNAM[1]	SMSQ10.100
GPZ1	10.00 %
P16	1000.00 usec

F1 - Acquisition parameters

TD	128
SFO1	500.2025 MHz
FIDRES	27.316433 Hz
SW	6.990 ppm
FnMODE	QF

F2 - Processing parameters

SI	1024
SF	500.2000000 MHz
WDW	QSINE
SSB	0
LB	0 Hz
GB	0
PC	1.40

F1 - Processing parameters

SI	1024
MC2	QF
SF	500.2000000 MHz
WDW	QSINE
SSB	0
LB	0 Hz
GB	0

CB7-MeOD-COSYGP

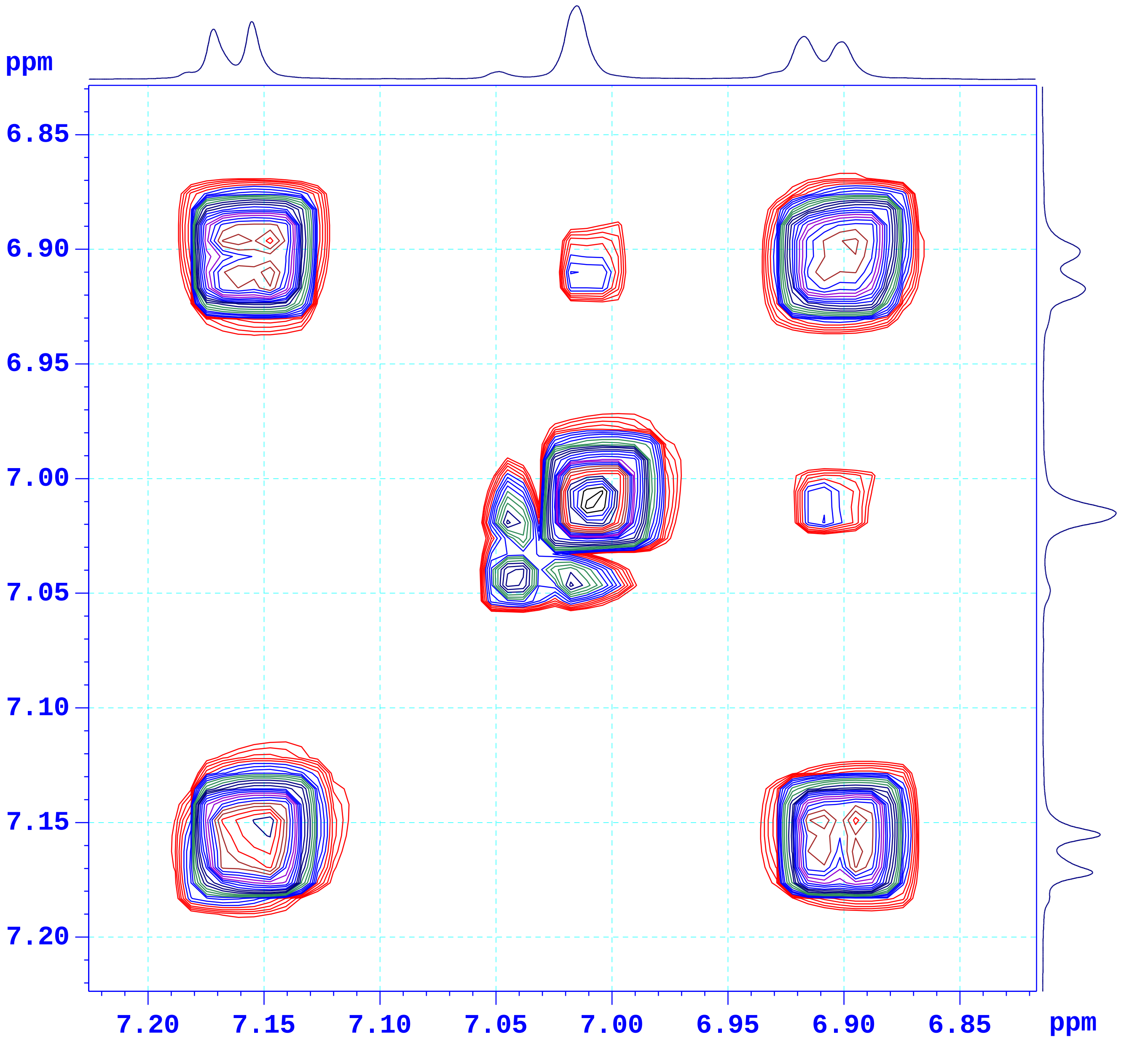

BRUKER

Current Data Parameters
NAME 11D_CB7
EXPNO 7
PROCNO 1

F2 - Acquisition Parameters
Date_ 20170703
Time 5.47
INSTRUM spect
PROBHD 5 mm PABBO BB/
PULPROG cosygpppqf
TD 2048
SOLVENT MeOD
NS 2
DS 8
SWH 3496.503 Hz
FIDRES 1.707277 Hz
AQ 0.2928640 sec
RG 14.29
DW 143.000 usec
DE 6.50 usec
TE 303.0 K
D0 0.00000300 sec
D1 1.86032605 sec
D11 0.03000000 sec
D12 0.00002000 sec
D13 0.00000400 sec
D16 0.00020000 sec
IN0 0.00028600 sec

======== CHANNEL f1 ========
SFO1 500.2024863 MHz
NUC1 1H
P0 10.00 usec
P1 10.00 usec
P17 2500.00 usec
PLW1 22.00000000 W
PLW10 3.25440001 W

====== GRADIENT CHANNEL =====
GPNAM[1] SMSQ10.100
GPZ1 10.00 %
P16 1000.00 usec

F1 - Acquisition parameters
TD 128
SFO1 500.2025 MHz
FIDRES 27.316433 Hz
SW 6.990 ppm
FnMODE QF

F2 - Processing parameters
SI 1024
SF 500.2000000 MHz
WDW QSINE
SSB 0
LB 0 Hz
GB 0
PC 1.40

F1 - Processing parameters
SI 1024
MC2 QF
SF 500.2000000 MHz
WDW QSINE
SSB 0
LB 0 Hz
GB 0
