# Supplementary material for: Chemical Constituents from Cimicifuga dahurica and Their Anti-Proliferative Effects on MCF-7 Breast Cancer Cells
Source: Molecules. 2018 May 4;23(5):1083. doi: 10.3390/molecules23051083 (PMC6102574; doi:10.3390/molecules23051083)
Supplement: Supplementary file 1 [file molecules-23-01083-s001.zip › Supplementary Materials_liping/Figure S26. ROESY spectrum of compound 7.pdf]

CB7-MeOD-ROESY

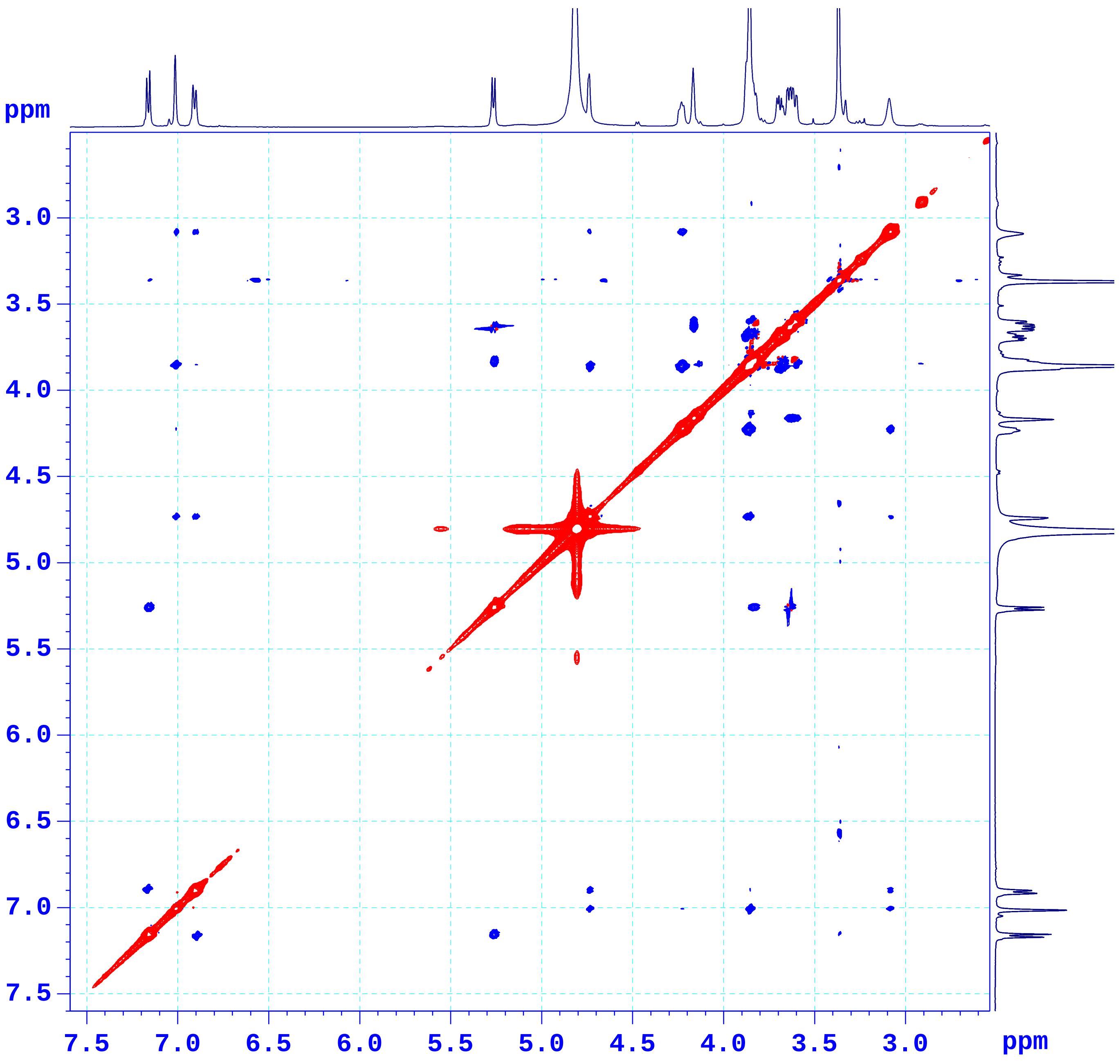

Current Data Parameters
NAME 11D_CB7
EXPNO 8
PROCNO 1

F2 - Acquisition Parameters
Date_ 20170703
Time 5.59
INSTRUM spect
PROBHD 5 mm PABBO BB/
PULPROG roesyphpp.2
TD 2048
SOLVENT MeOD
NS 4
DS 32
SWH 3521.127 Hz
FIDRES 1.719300 Hz
AQ 0.2908160 sec
RG 14.29
DW 142.000 usec
DE 6.50 usec
TE 303.0 K
D0 0.00013163 sec
D1 1.91398394 sec
D11 0.03000000 sec
D12 0.00002000 sec
IN0 0.00028400 sec
L4 1000
P15 200000.00 usec

======== CHANNEL f1 ========
SFO1 500.2024895 MHz
NUC1 1H
P1 10.00 usec
P17 2500.00 usec
P25 100.00 usec
PLW1 22.00000000 W
PLW10 3.25440001 W
PLW27 0.88000000 W

F1 - Acquisition parameters
TD 256
SFO1 500.2025 MHz
FIDRES 13.754401 Hz
SW 7.039 ppm
FnMODE States-TPPI

F2 - Processing parameters
SI 1024
SF 500.2000038 MHz
WDW QSINE
SSB 2
LB 0 Hz
GB 0
PC 1.00

F1 - Processing parameters
SI 1024
MC2 States-TPPI
SF 500.2000042 MHz
WDW QSINE
SSB 2
LB 0 Hz
GB 0

CB7-MeOD-ROESY

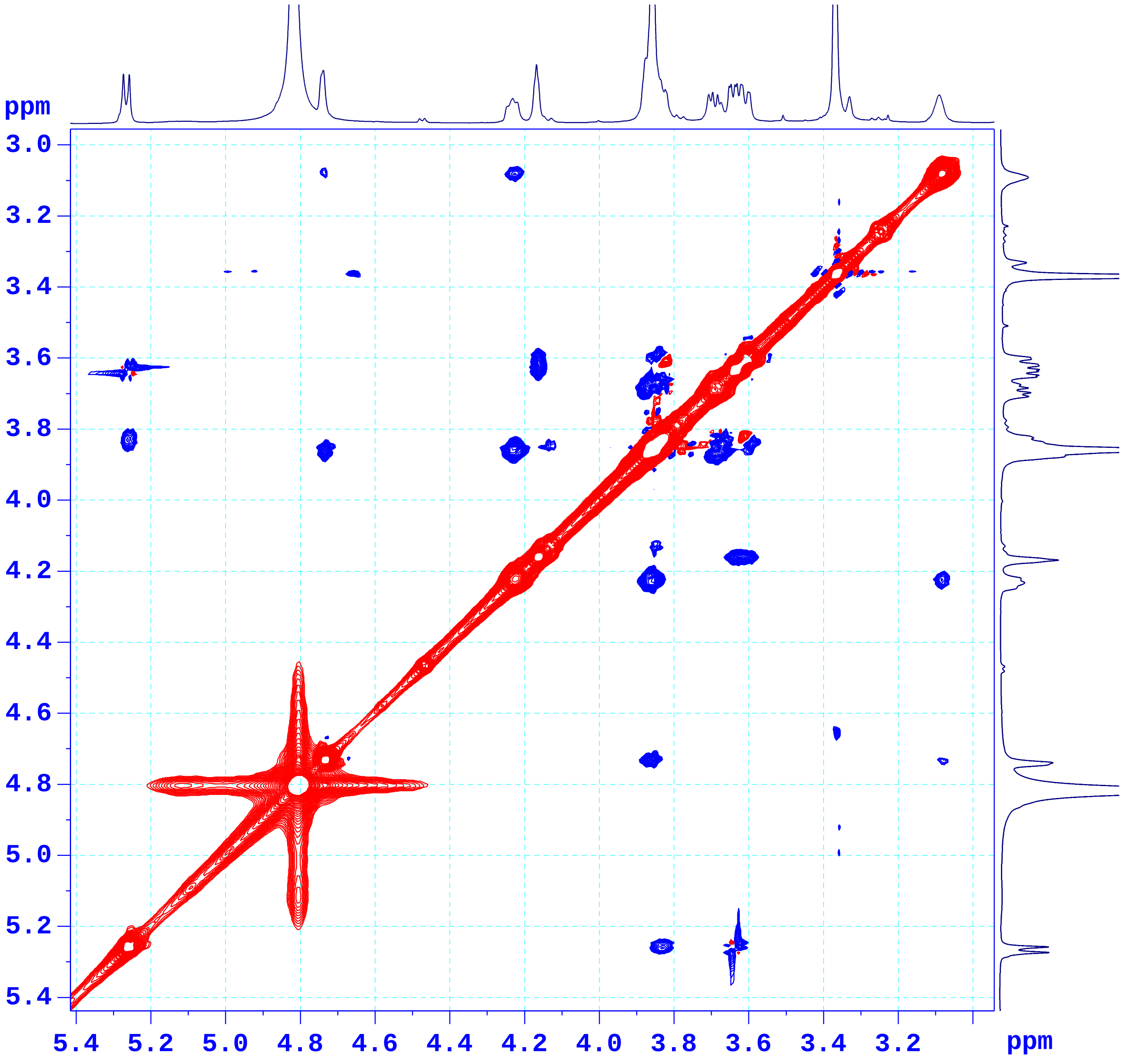

BRUKER

Current Data Parameters	
NAME	11D_CB7
EXPNO	8
PROCNO	1

F2 - Acquisition Parameters	
Date_	20170703
Time	5.59
INSTRUM	spect
PROBHD	5 mm PABBO BB/
PULPROG	roesyphpp.2
TD	2048
SOLVENT	MeOD
NS	4
DS	32
SWH	3521.127 Hz
FIDRES	1.719300 Hz
AQ	0.2908160 sec
RG	14.29
DW	142.000 usec
DE	6.50 usec
TE	303.0 K
D0	0.00013163 sec
D1	1.91398394 sec
D11	0.03000000 sec
D12	0.00002000 sec
IN0	0.00028400 sec
L4	1000
P15	200000.00 usec

======== CHANNEL f1 ========	
SFO1	500.2024895 MHz
NUC1	1H
P1	10.00 usec
P17	2500.00 usec
P25	100.00 usec
PLW1	22.00000000 W
PLW10	3.25440001 W
PLW27	0.88000000 W

F1 - Acquisition parameters	
TD	256
SFO1	500.2025 MHz
FIDRES	13.754401 Hz
SW	7.039 ppm
FnMODE	States-TPPI

F2 - Processing parameters	
SI	1024
SF	500.2000038 MHz
WDW	QSINE
SSB	2
LB	0 Hz
GB	0
PC	1.00

F1 - Processing parameters	
SI	1024
MC2	States-TPPI
SF	500.2000042 MHz
WDW	QSINE
SSB	2
LB	0 Hz
GB	0

CB7-MeOD-ROESY

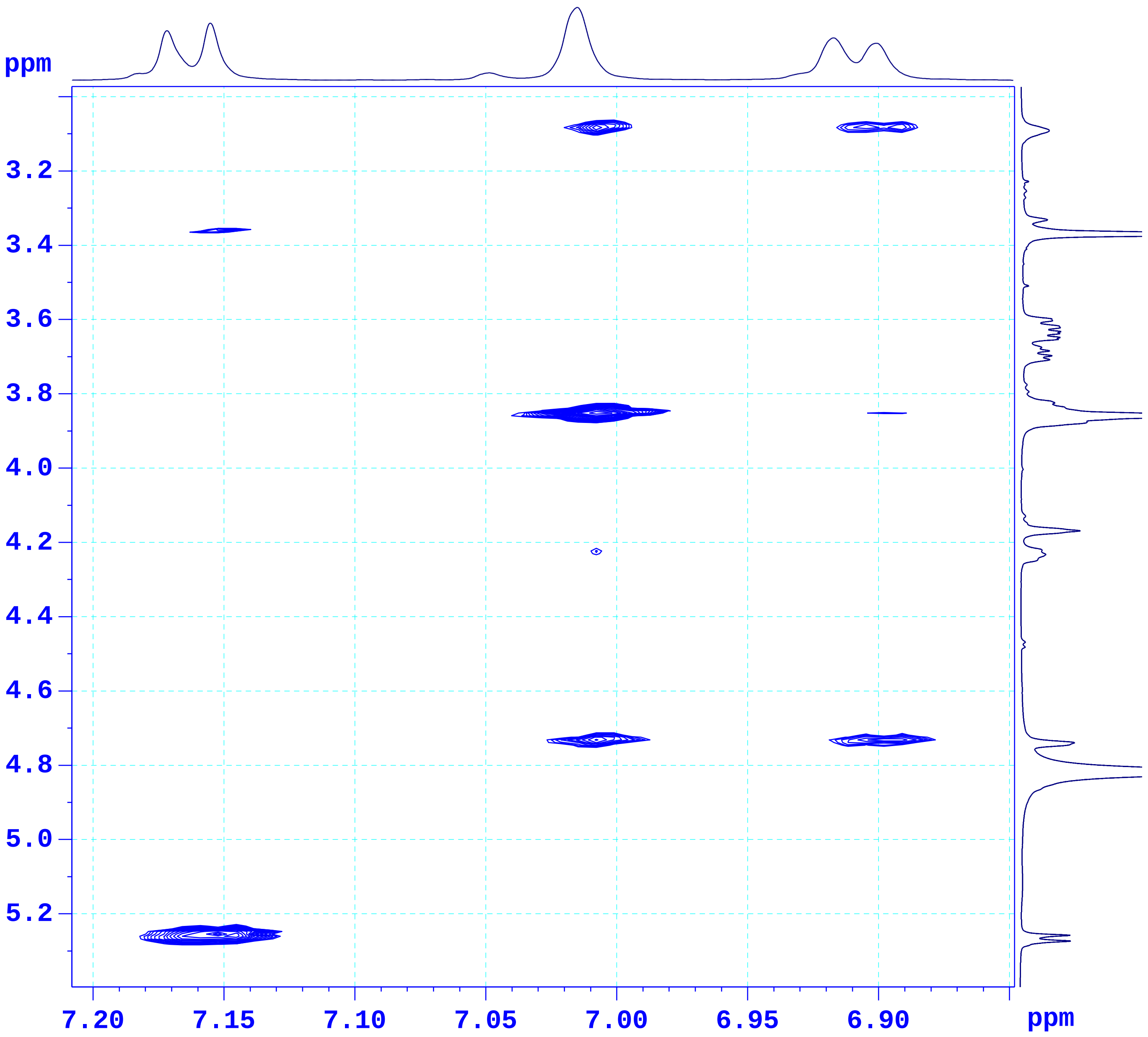

Current Data Parameters

NAME	11D_CB7
EXPNO	8
PROCNO	1

F2 - Acquisition Parameters

Date_	20170703
Time	5.59
INSTRUM	spect
PROBHD	5 mm PABBO BB/
PULPROG	roesyphpp.2
TD	2048
SOLVENT	MeOD
NS	4
DS	32
SWH	3521.127 Hz
FIDRES	1.719300 Hz
AQ	0.2908160 sec
RG	14.29
DW	142.000 usec
DE	6.50 usec
TE	303.0 K
D0	0.00013163 sec
D1	1.91398394 sec
D11	0.03000000 sec
D12	0.00002000 sec
IN0	0.00028400 sec
L4	1000
P15	200000.00 usec

======== CHANNEL f1 ========

SFO1	500.2024895 MHz
NUC1	1H
P1	10.00 usec
P17	2500.00 usec
P25	100.00 usec
PLW1	22.00000000 W
PLW10	3.25440001 W
PLW27	0.88000000 W

F1 - Acquisition parameters

TD	256
SFO1	500.2025 MHz
FIDRES	13.754401 Hz
SW	7.039 ppm
FnMODE	States-TPPI

F2 - Processing parameters

SI	1024
SF	500.2000038 MHz
WDW	QSINE
SSB	2
LB	0 Hz
GB	0
PC	1.00

F1 - Processing parameters

SI	1024
MC2	States-TPPI
SF	500.2000042 MHz
WDW	QSINE
SSB	2
LB	0 Hz
GB	0
