# Supplementary figures and images for: Chemical Constituents from Cimicifuga dahurica and Their Anti-Proliferative Effects on MCF-7 Breast Cancer Cells
Source: Molecules. 2018 May 4;23(5):1083. doi: 10.3390/molecules23051083 (PMC6102574; doi:10.3390/molecules23051083)

# CB3-MeOD-C13CPD &DEPT

DEPT90

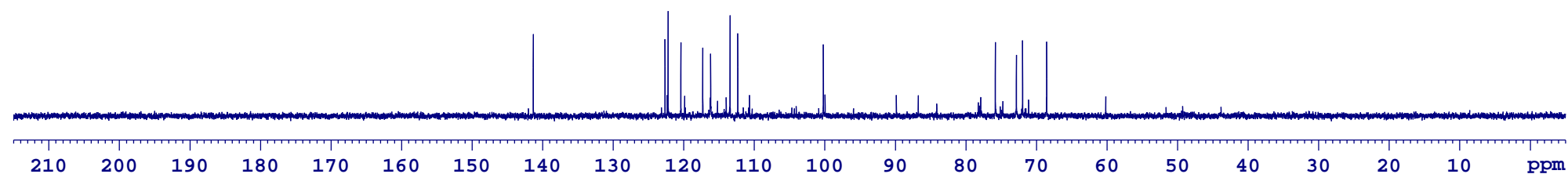

DEPT135

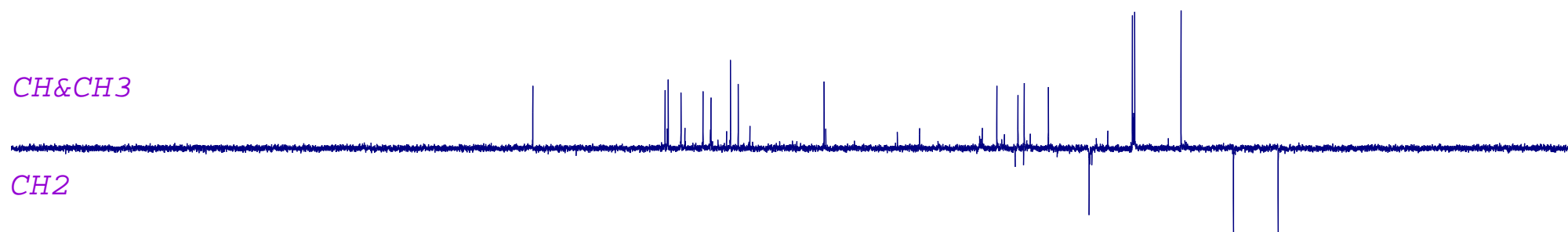

CH&CH3

CH2

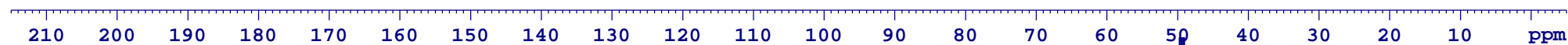

C13CPD

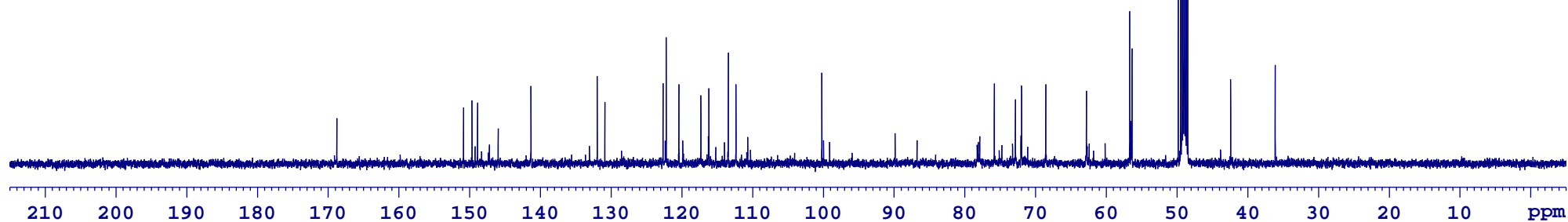

# CB3-MeOD-C13CPD &DEPT

DEPT90

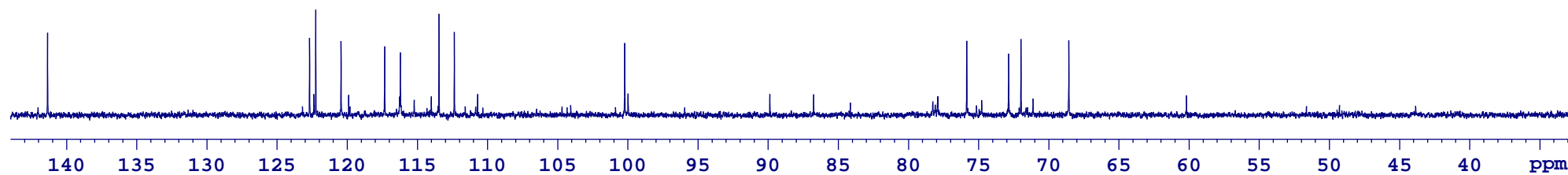

DEPT135

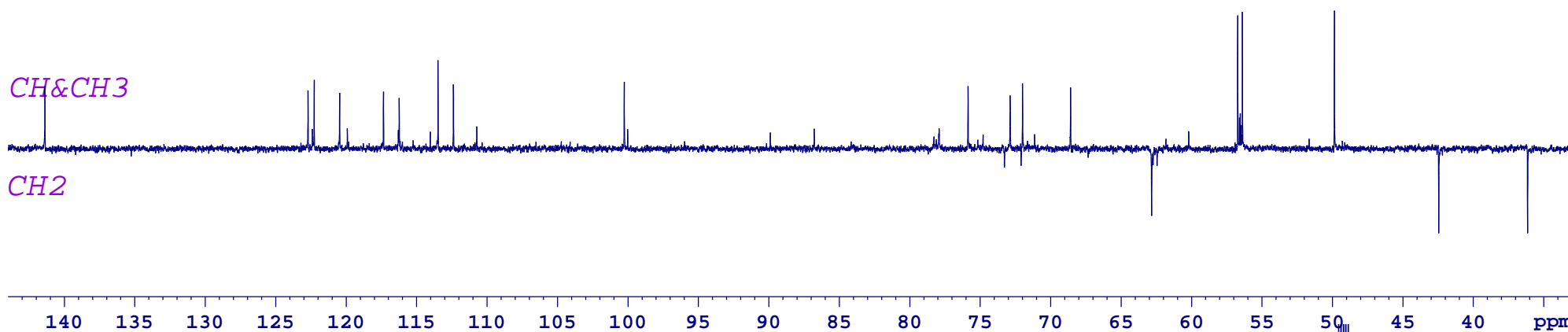

C13CPD

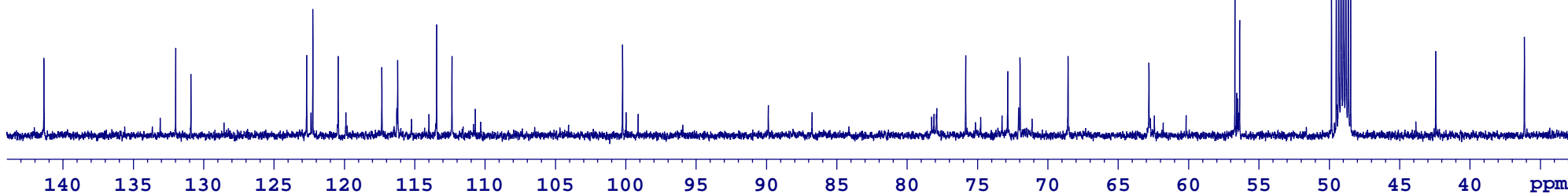

Supplement: Supplementary file 1 [file molecules-23-01083-s001.zip › Supplementary Materials_liping/Figure S3. DEPT spectrum of compound 3.pdf]

*CB3-MeOD-HMBC*

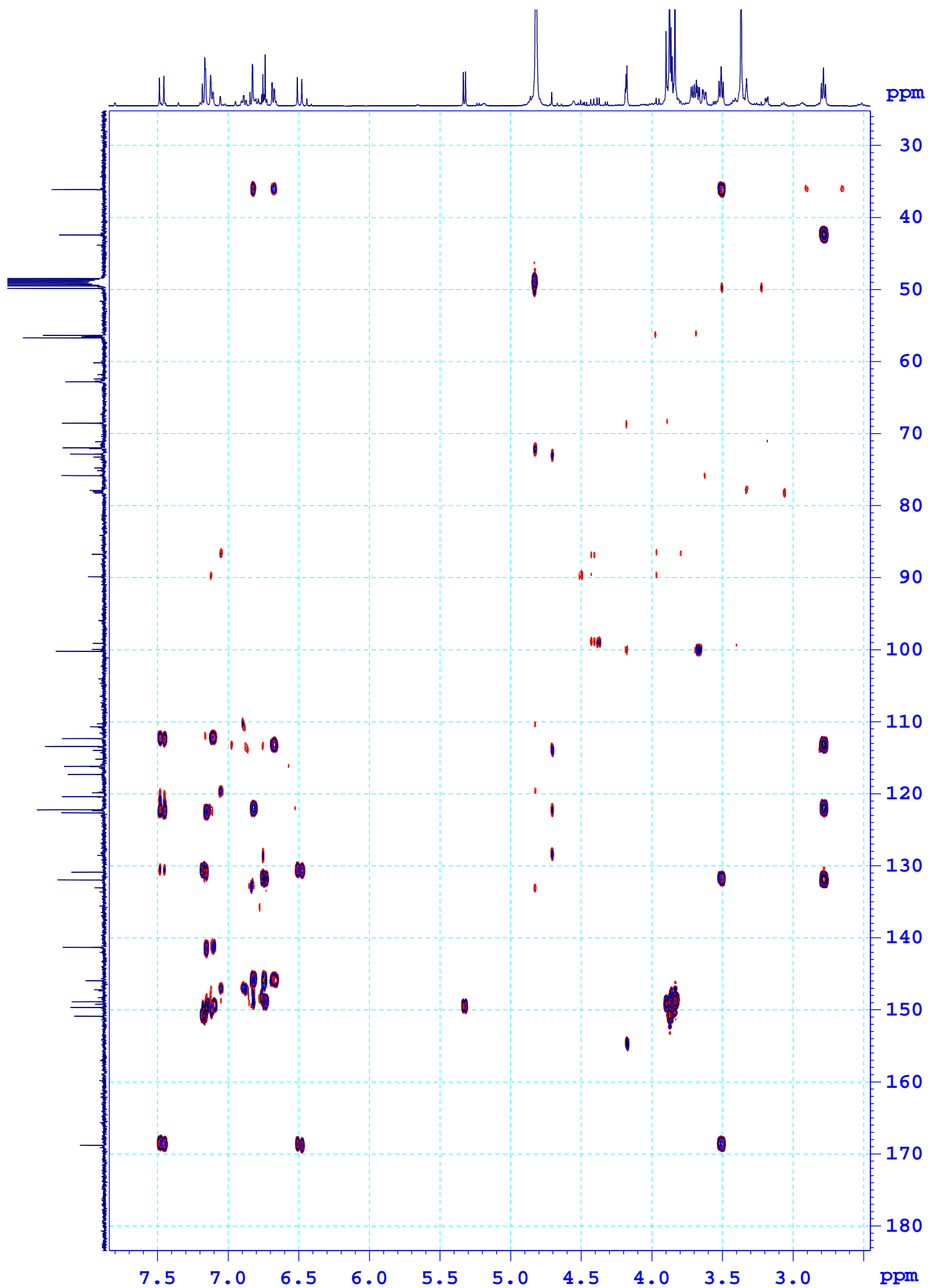

*CB3-MeOD-HMBC*

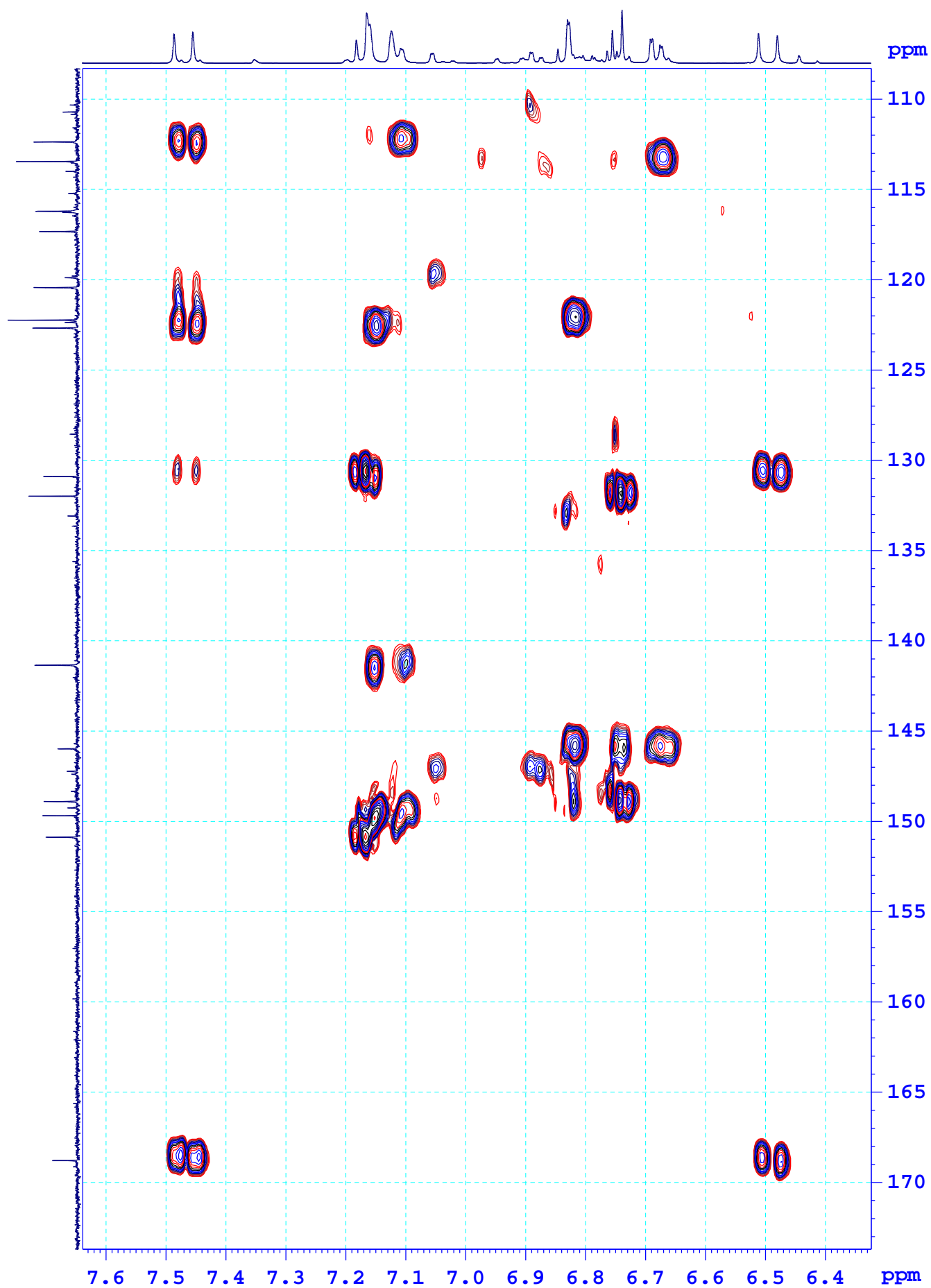

*CB3-MeOD-HMBC*

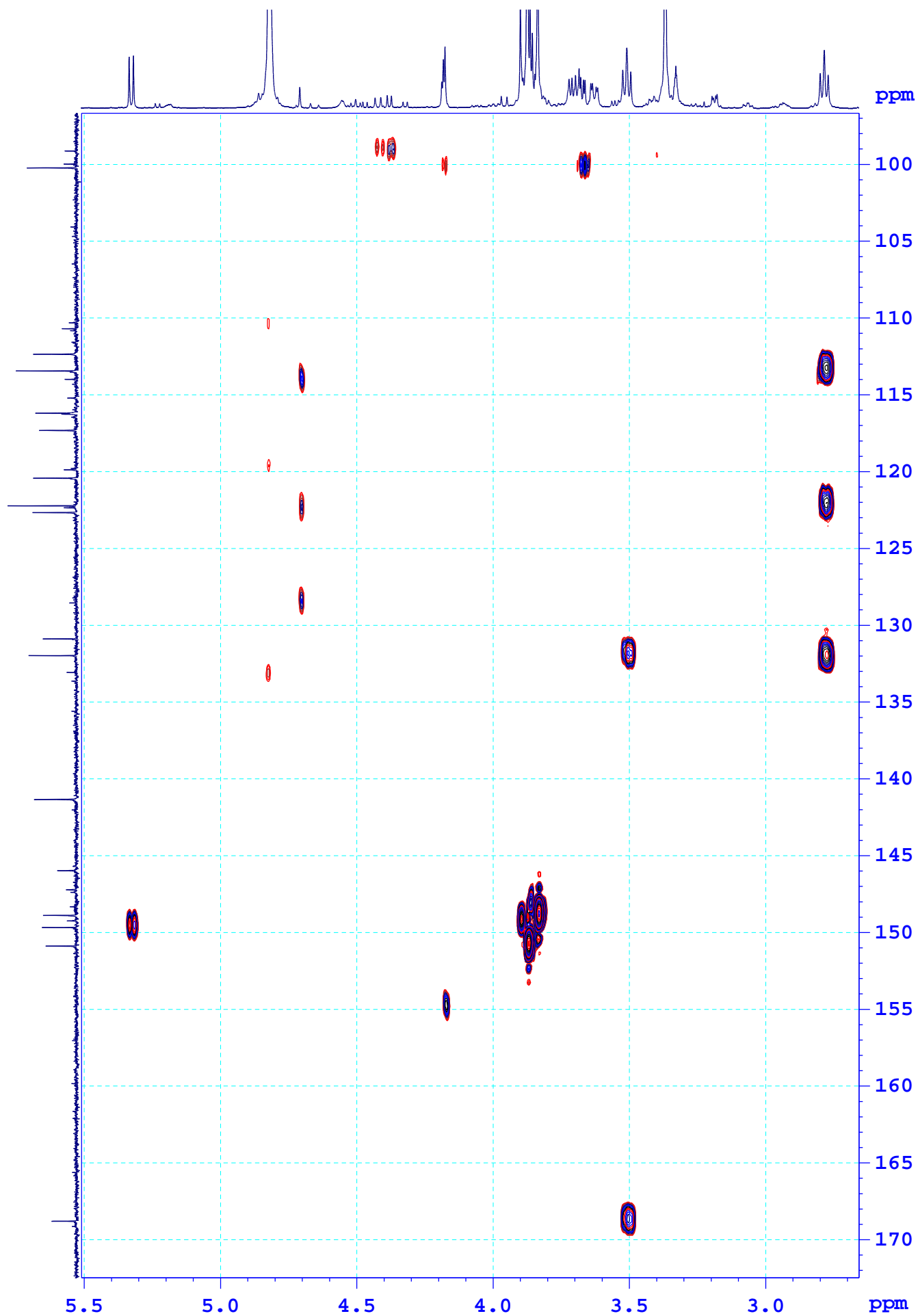

*CB3-MeOD-HMBC*

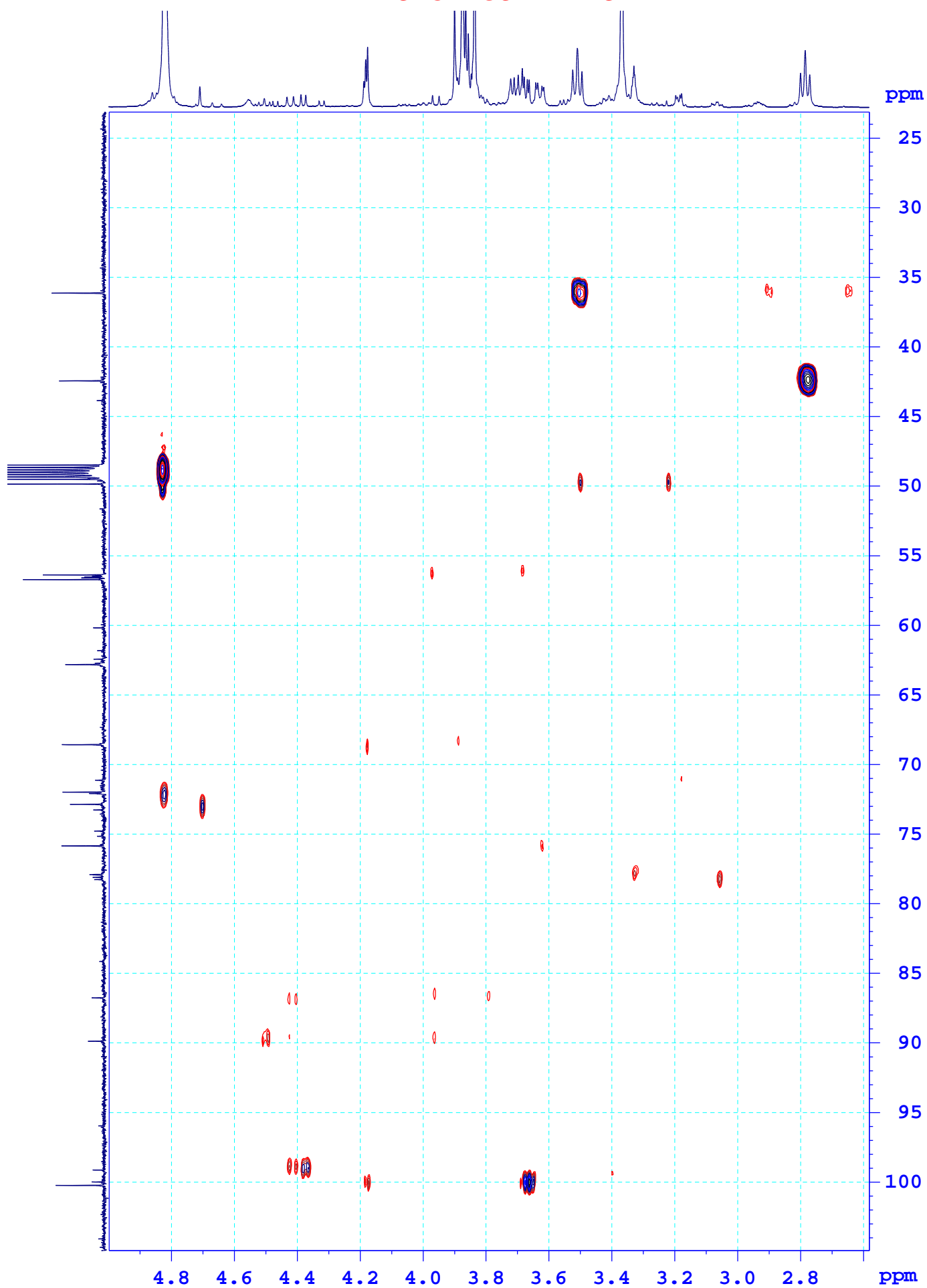

*CB3-MeOD-HMBC*

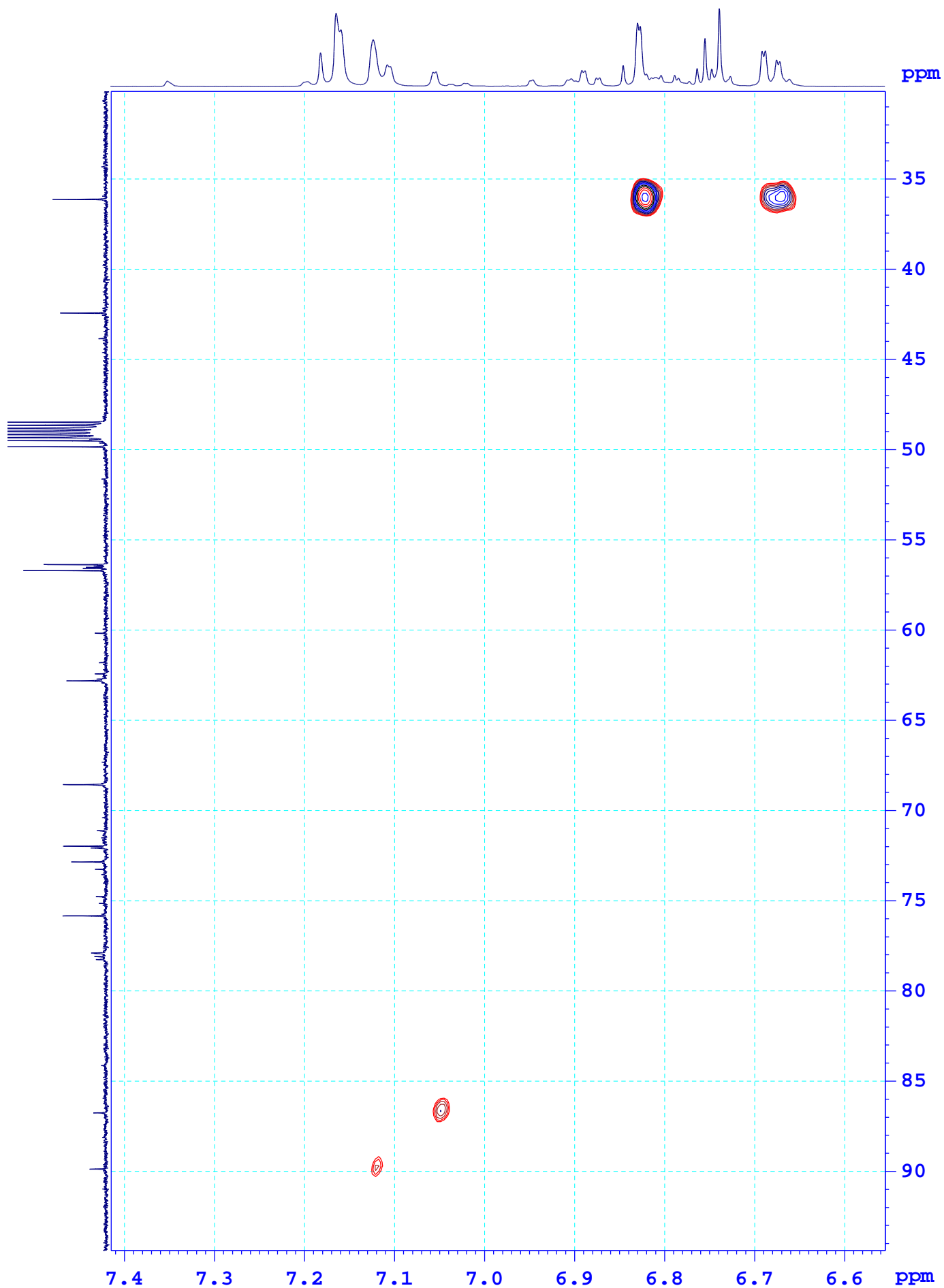

Supplement: Supplementary file 1 [file molecules-23-01083-s001.zip › Supplementary Materials_liping/Figure S4. HMBC spectrum of compound 3.pdf]

*CB3-MeOD-HSQC*

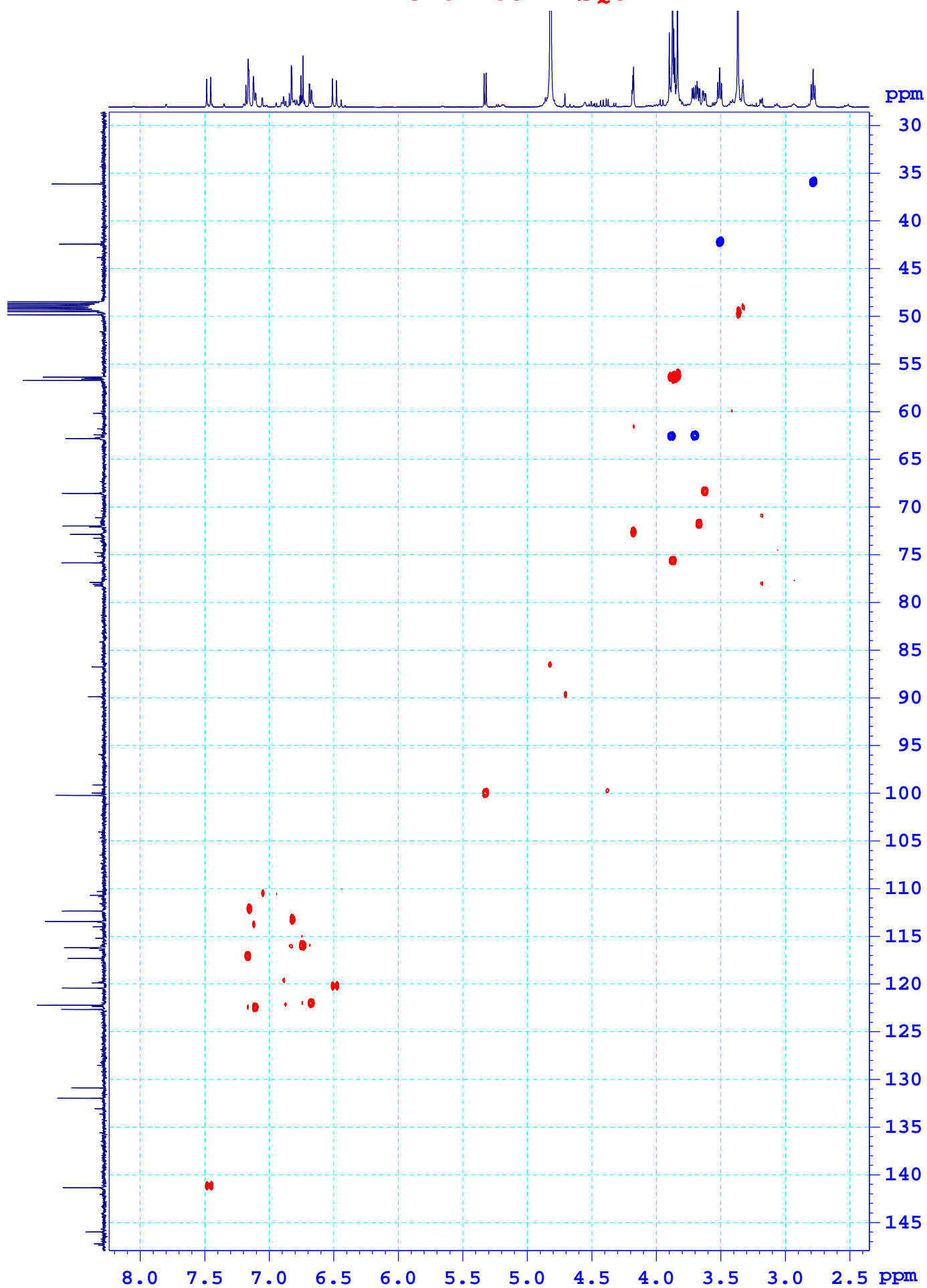

*CB3-MeOD-HSQC*

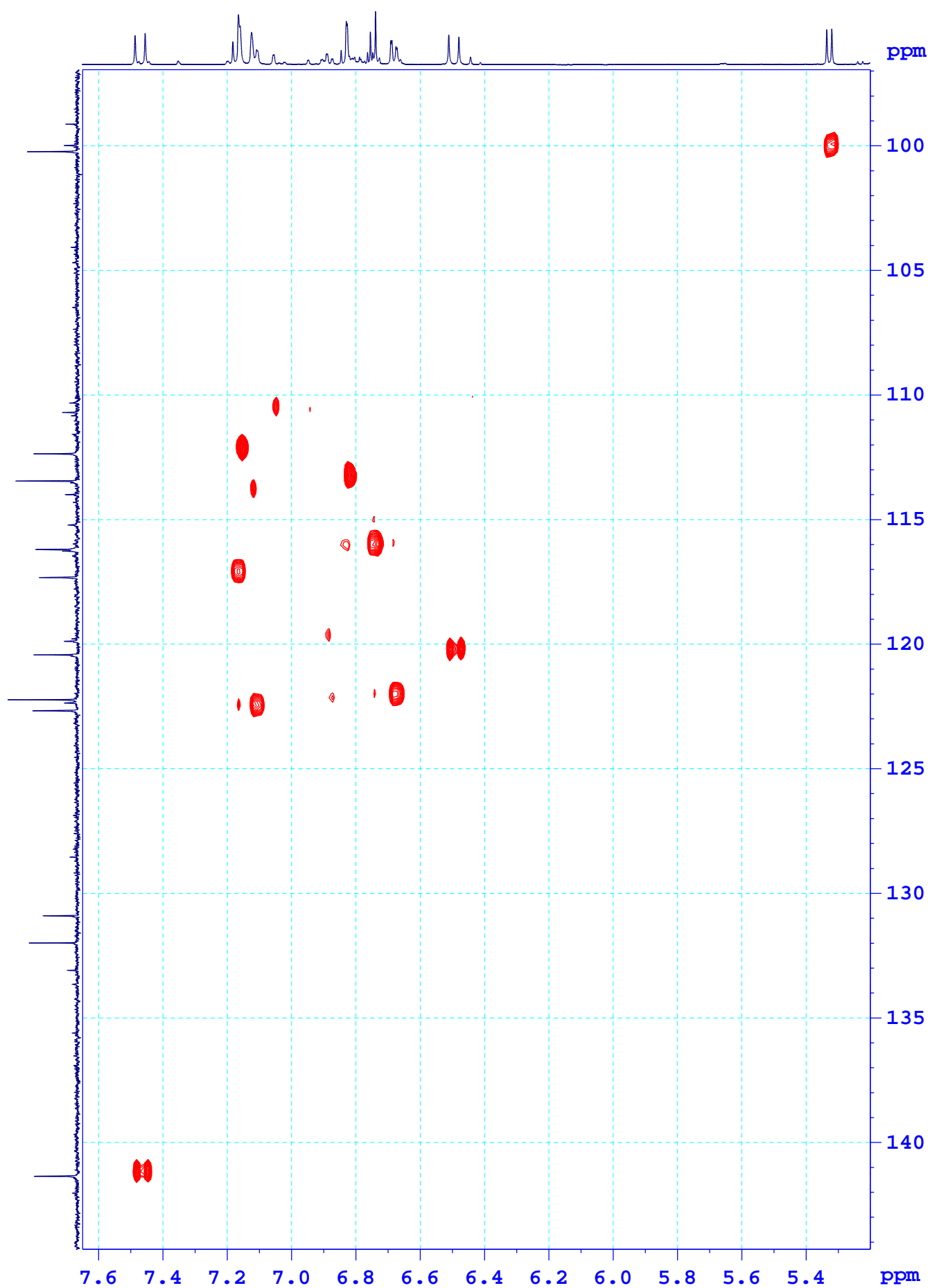

*CB3-MeOD-HSQC*

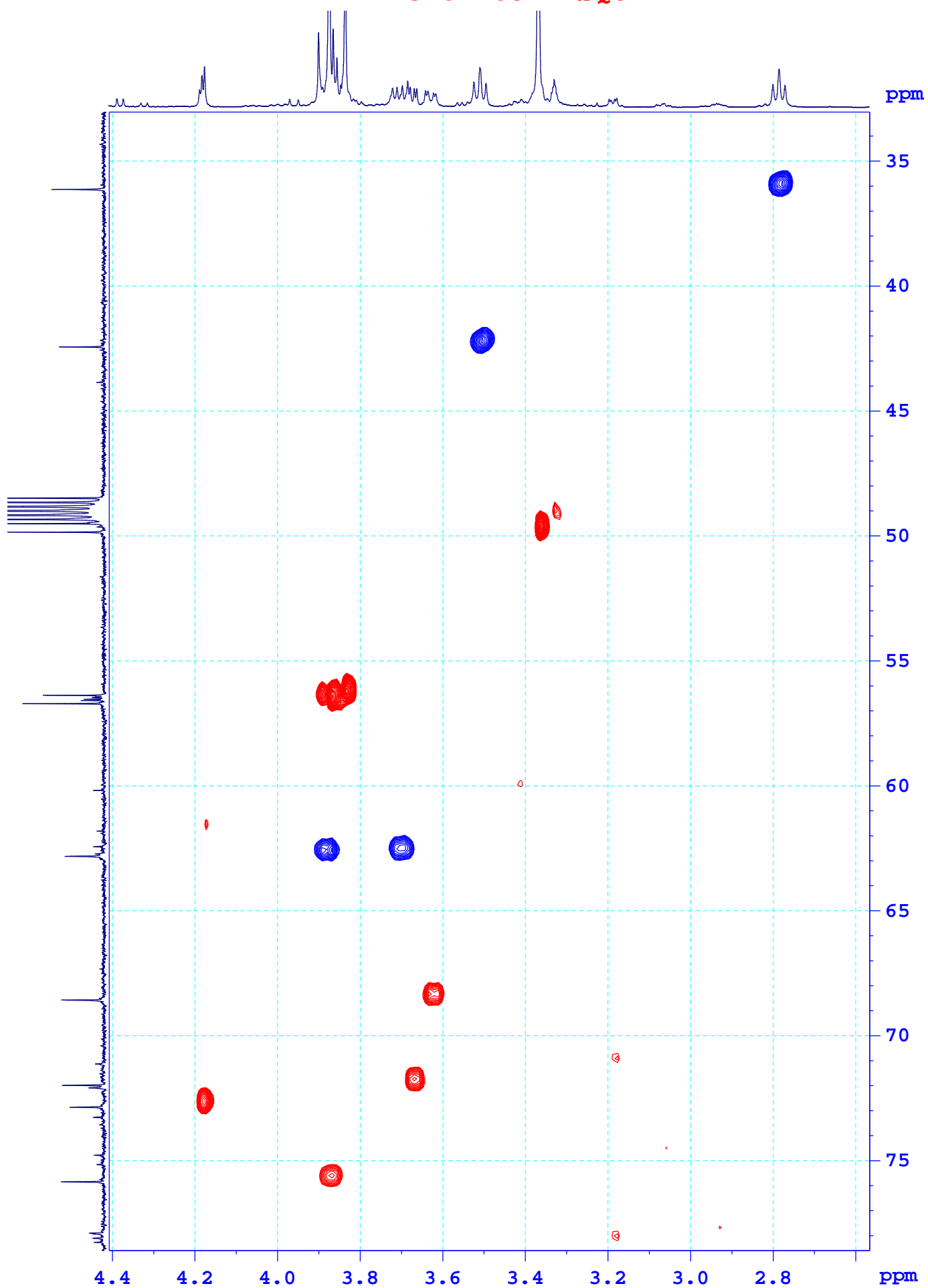

Supplement: Supplementary file 1 [file molecules-23-01083-s001.zip › Supplementary Materials_liping/Figure S5. HSQC spectrum of compound 3.pdf]

# CB4-MeOD-C13CPD &DEPT

DEPT90

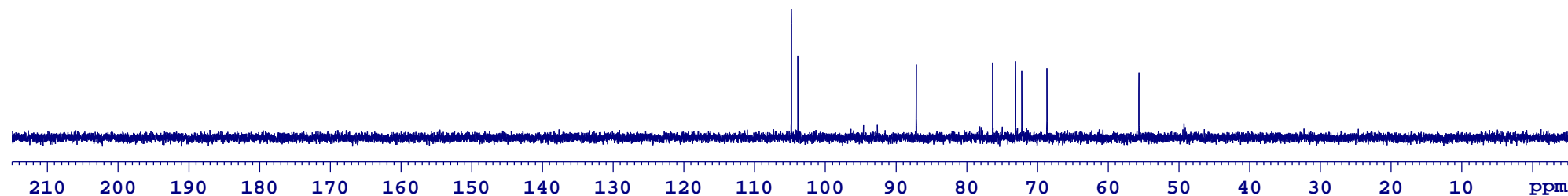

DEPT135

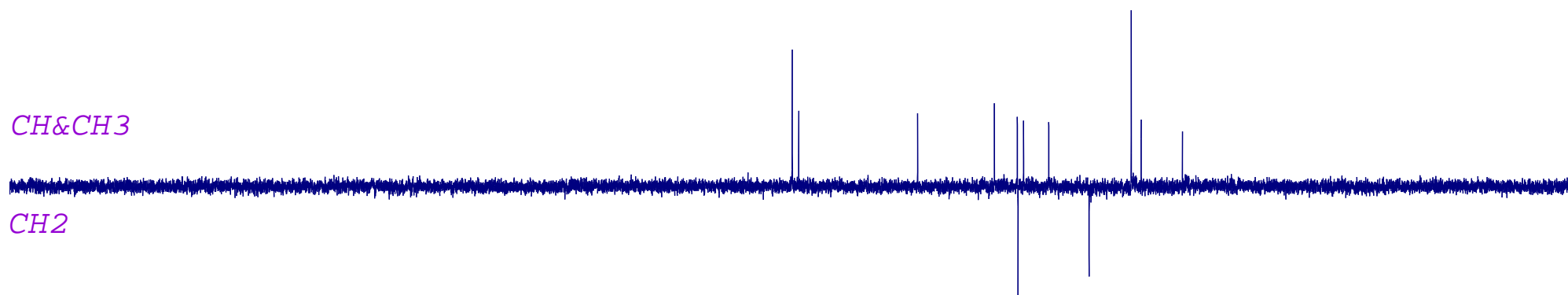

CH&CH3

CH2

C13CPD

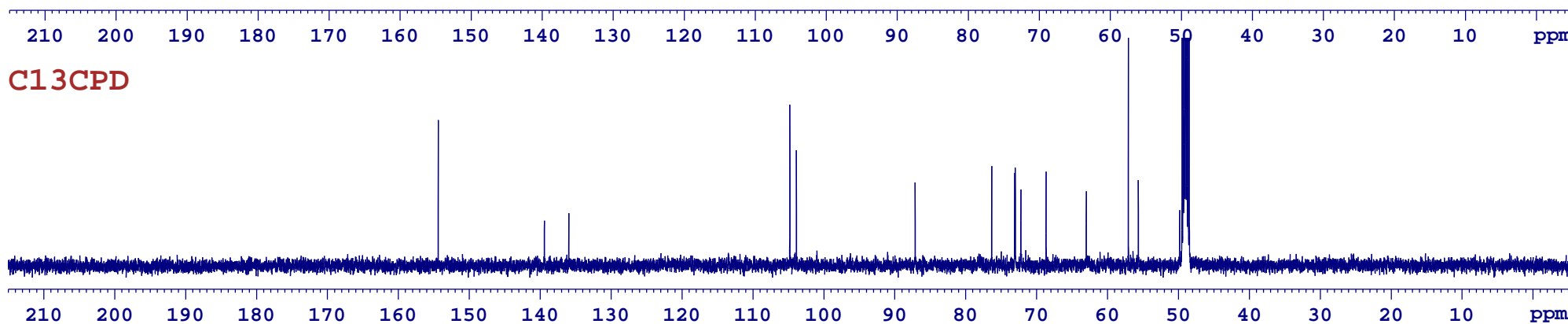

# CB4-MeOD-C13CPD &DEPT

DEPT90

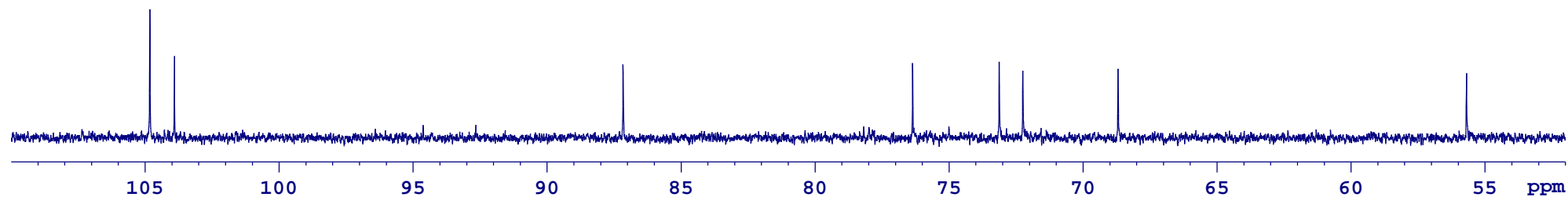

DEPT135

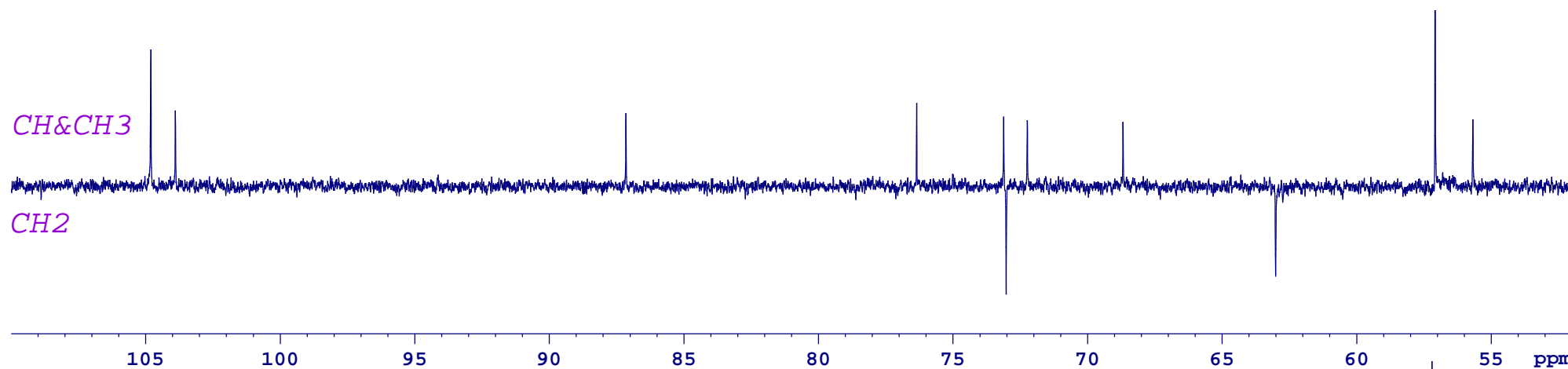

C13CPD

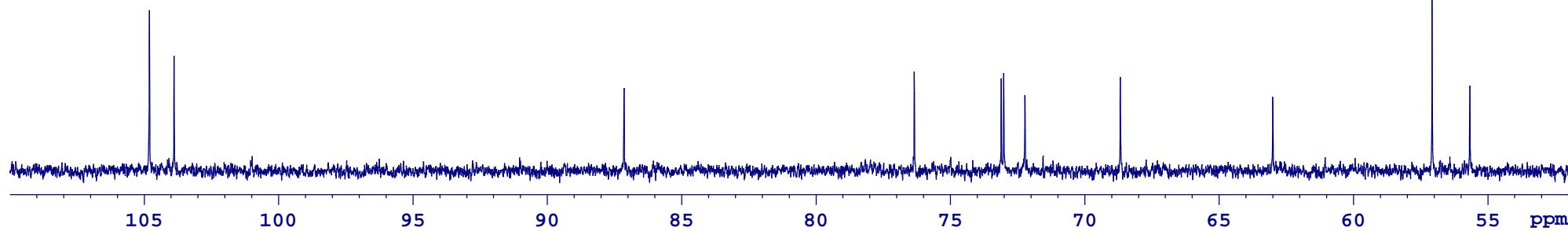

Supplement: Supplementary file 1 [file molecules-23-01083-s001.zip › Supplementary Materials_liping/Figure S9. DEPT spectrum of compound 4.pdf]

*CB4-MeOD-HMBC*

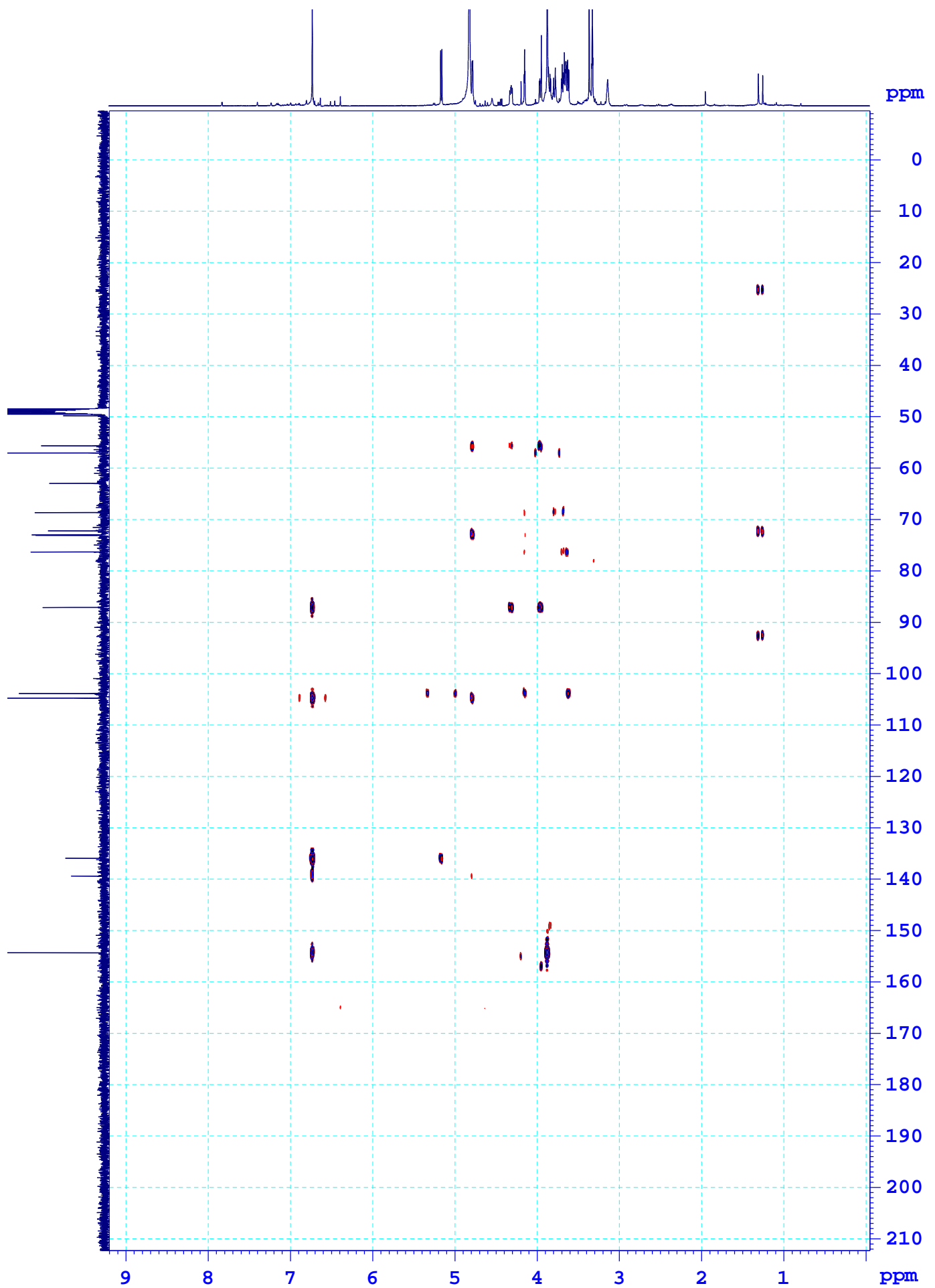

*CB4-MeOD-HMBC*

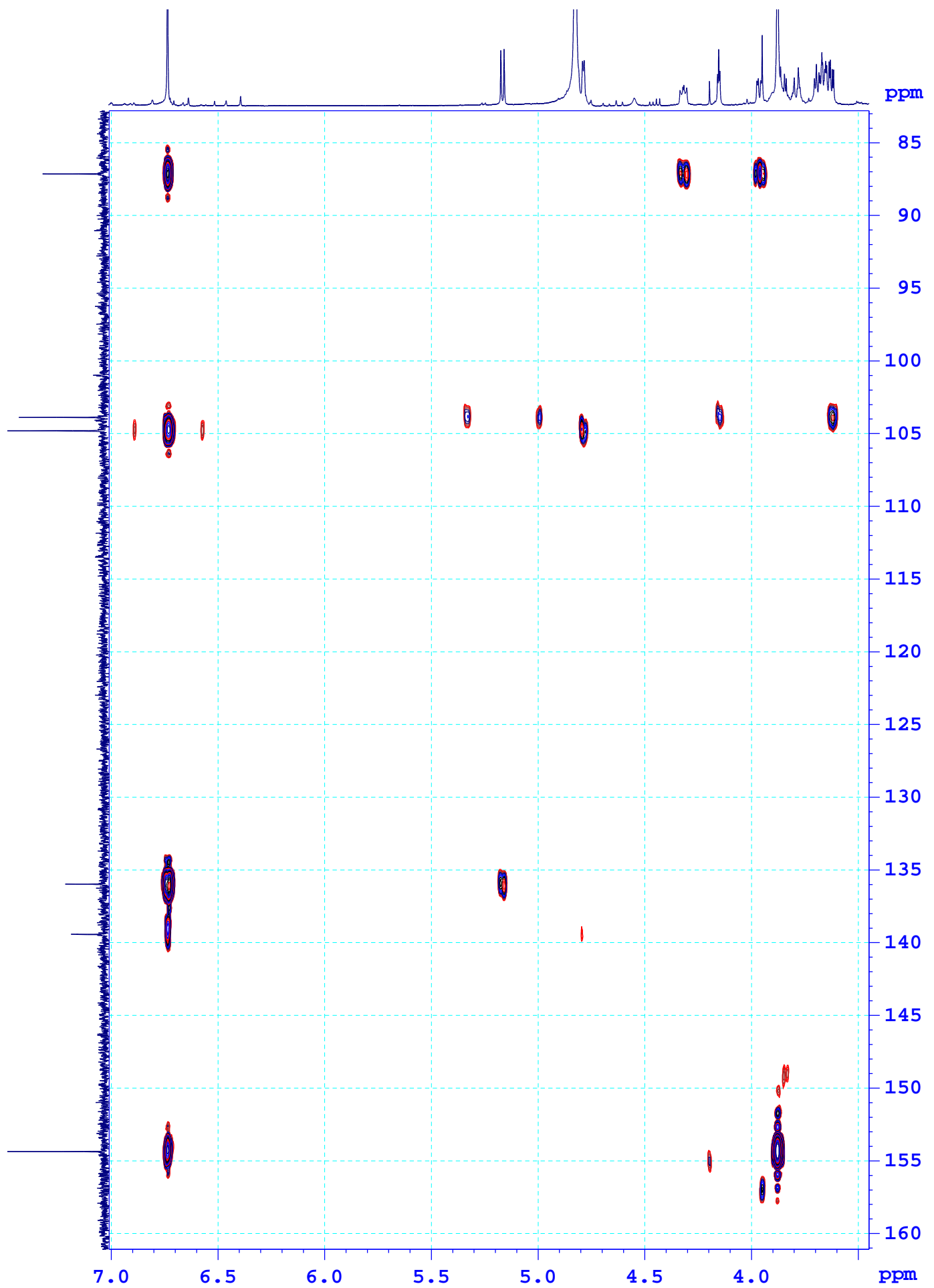

*CB4-MeOD-HMBC*

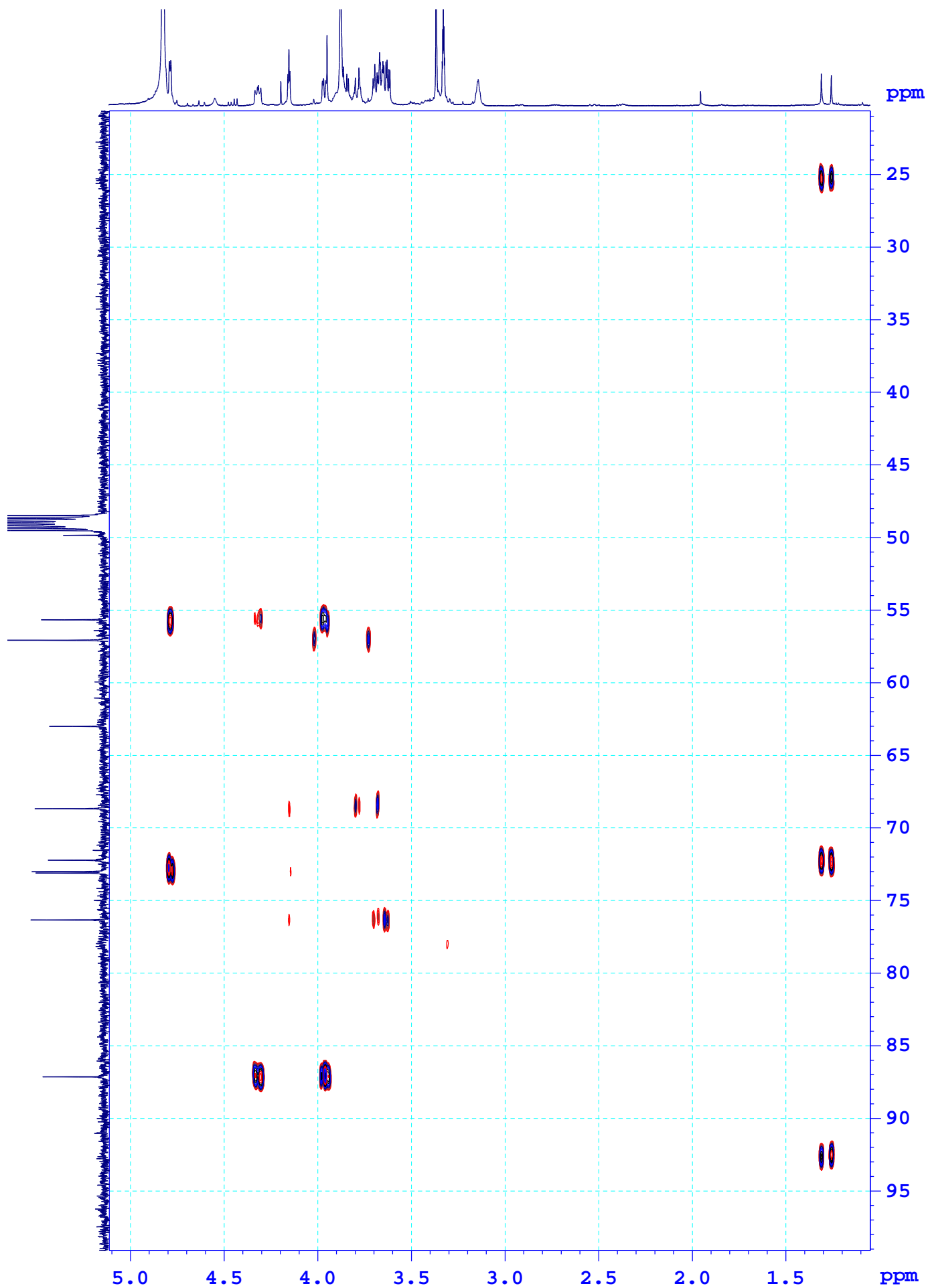

*CB4-MeOD-HMBC*

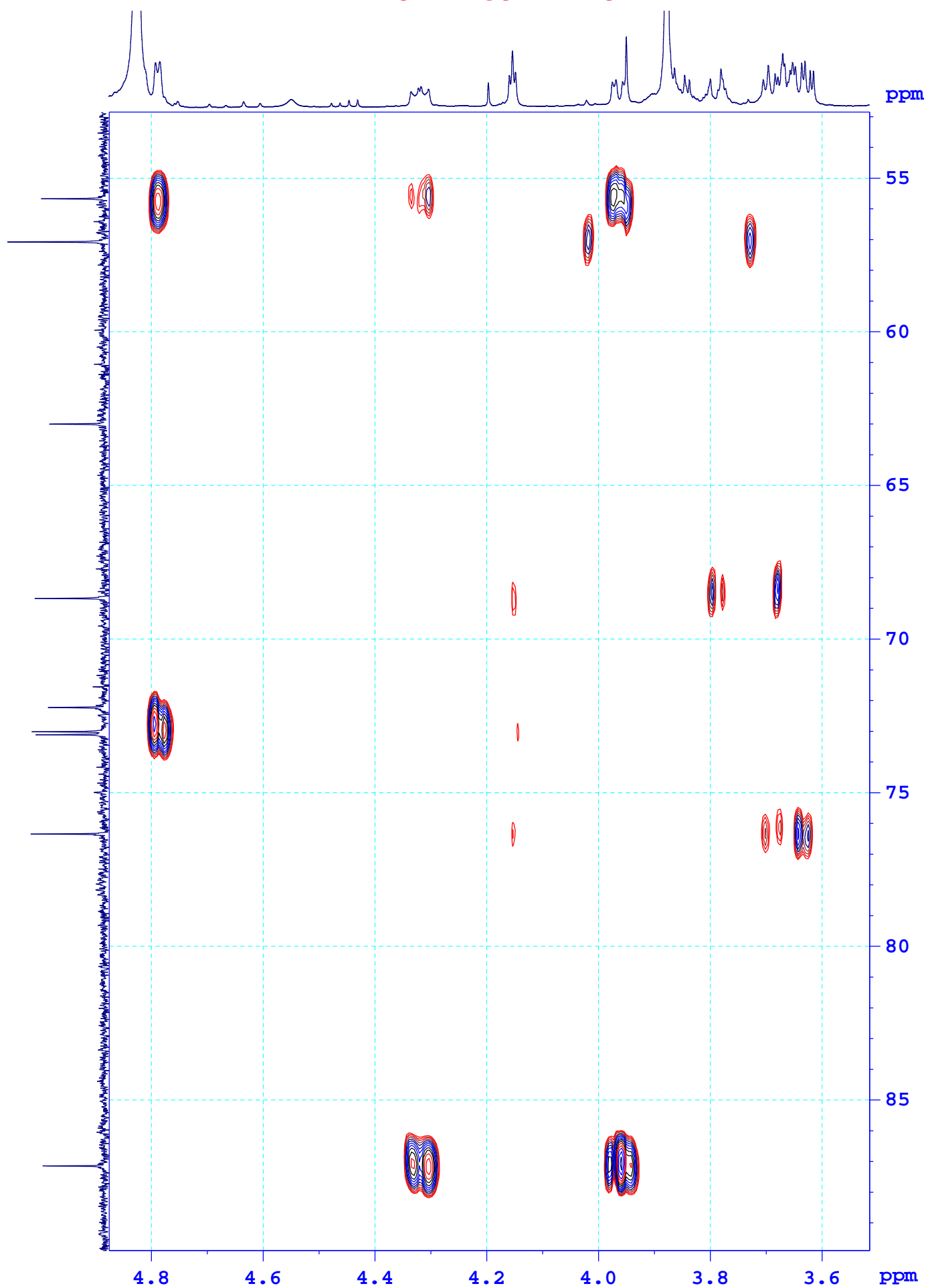

Supplement: Supplementary file 1 [file molecules-23-01083-s001.zip › Supplementary Materials_liping/Figure S10. HMBC spectrum of compound 4.pdf]

*CB4-MeOD-HSQC*

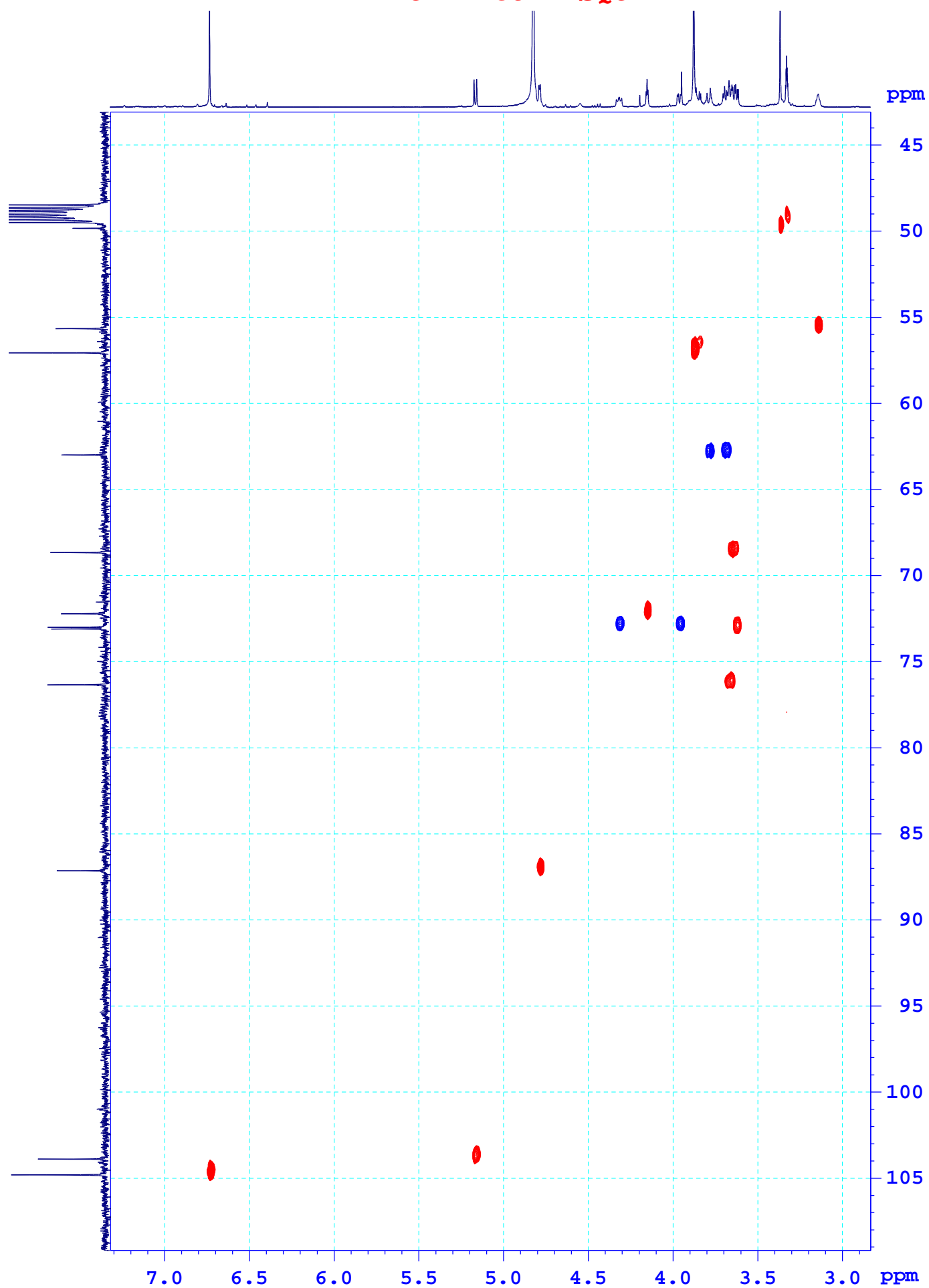

*CB4-MeOD-HSQC*

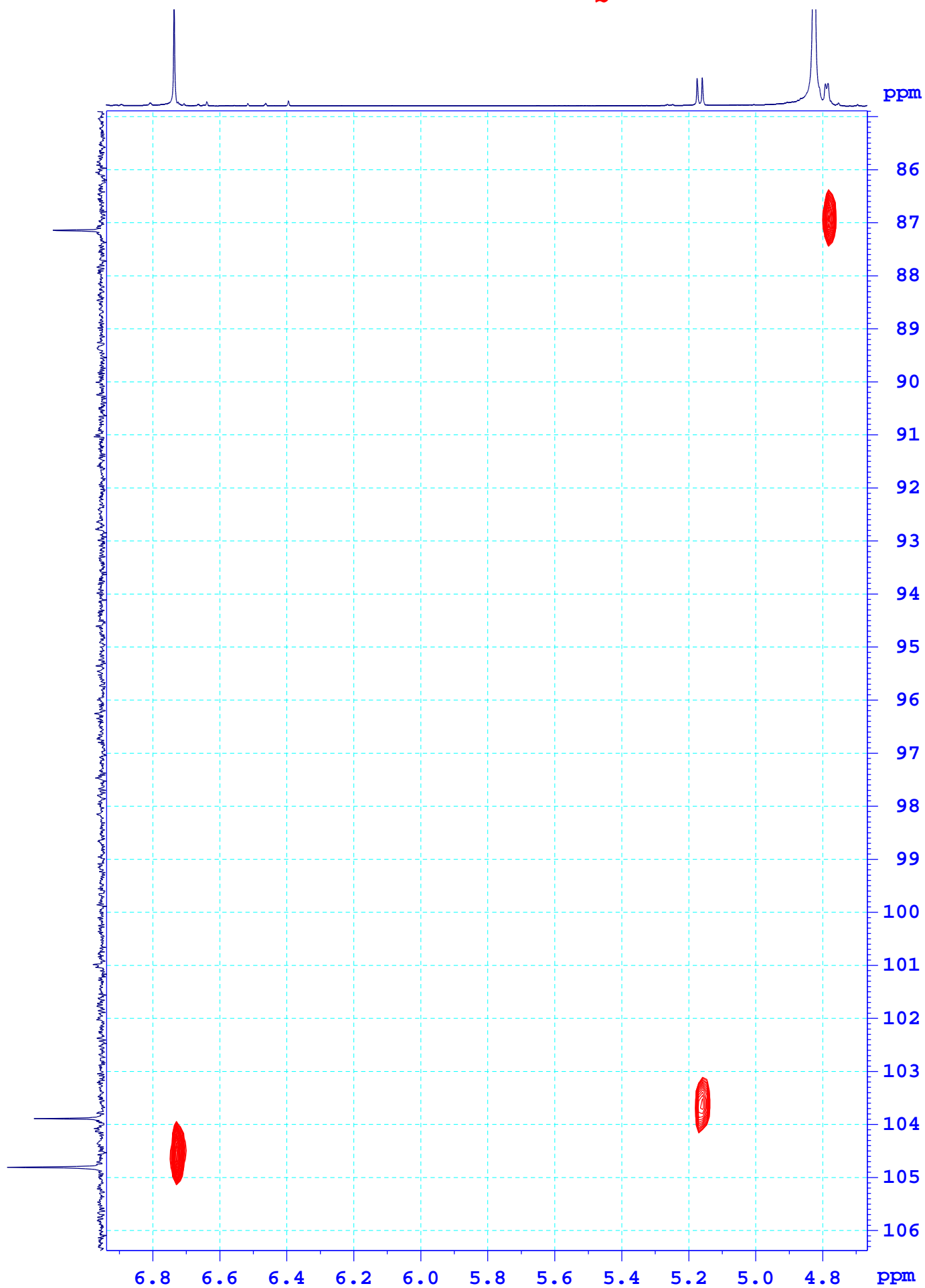

*CB4-MeOD-HSQC*

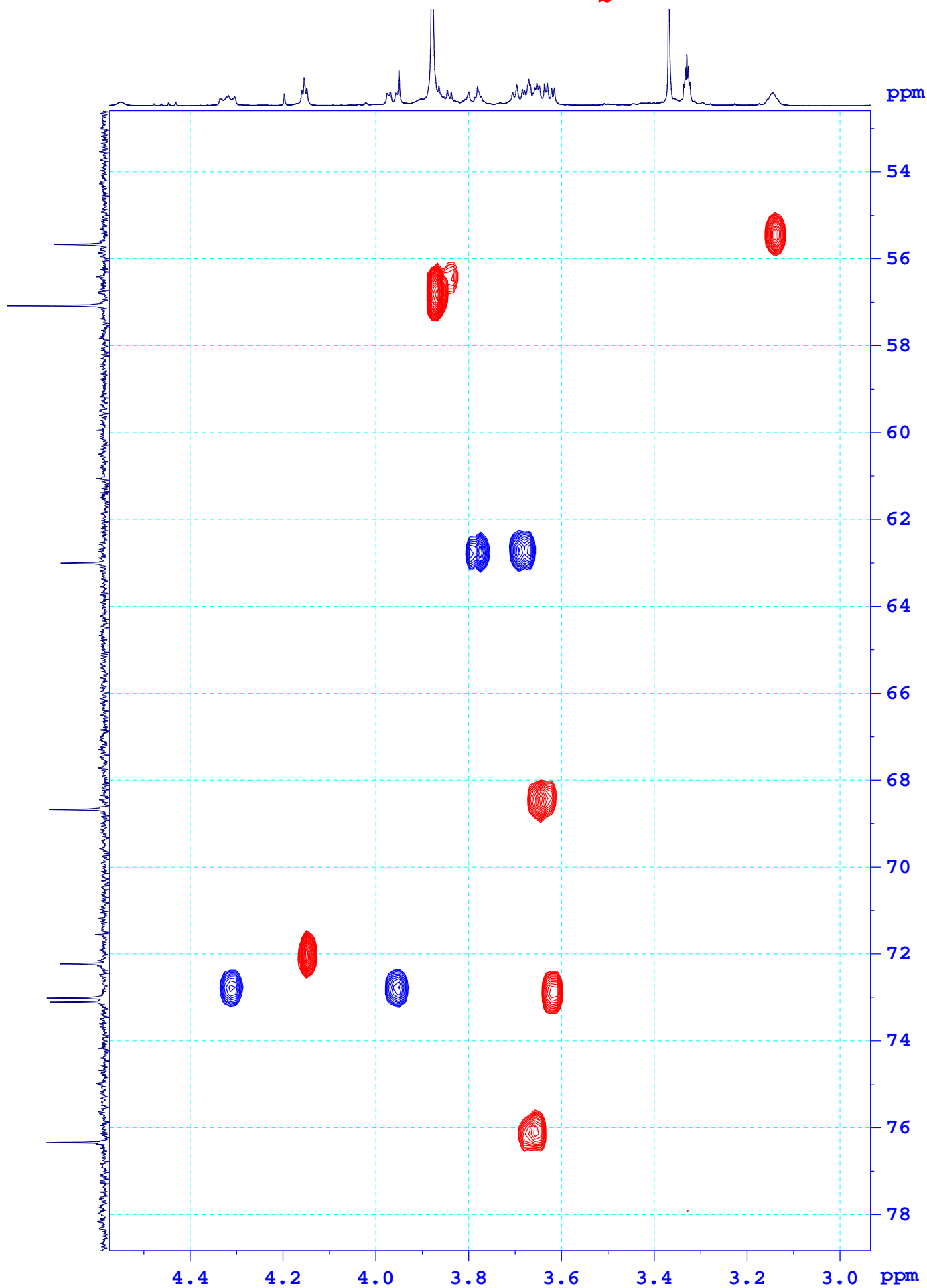

Supplement: Supplementary file 1 [file molecules-23-01083-s001.zip › Supplementary Materials_liping/Figure S11. HSQC spectrum of compound 4.pdf]

*CB6-MeOD-HMBC*

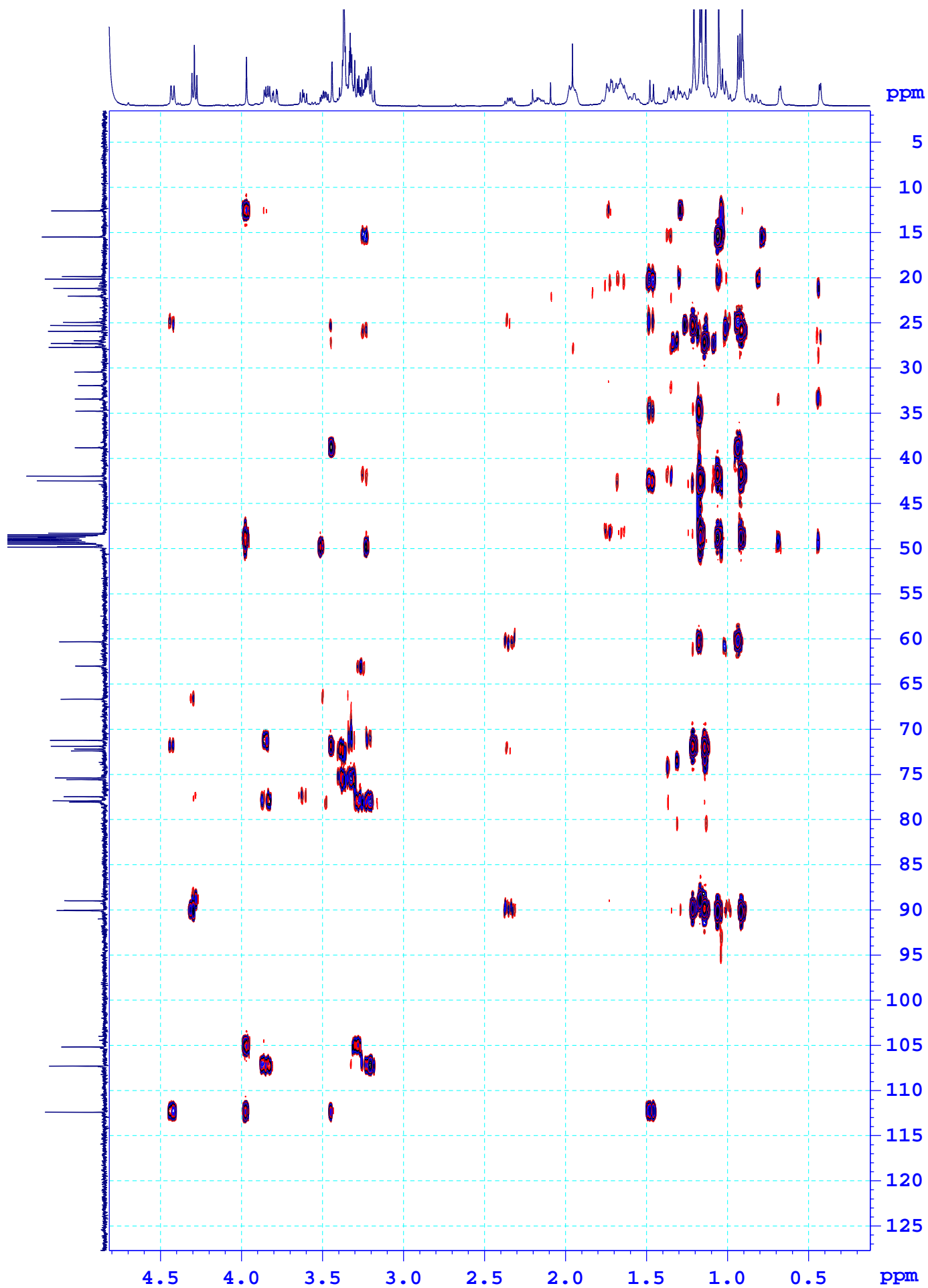

*CB6-MeOD-HMBC*

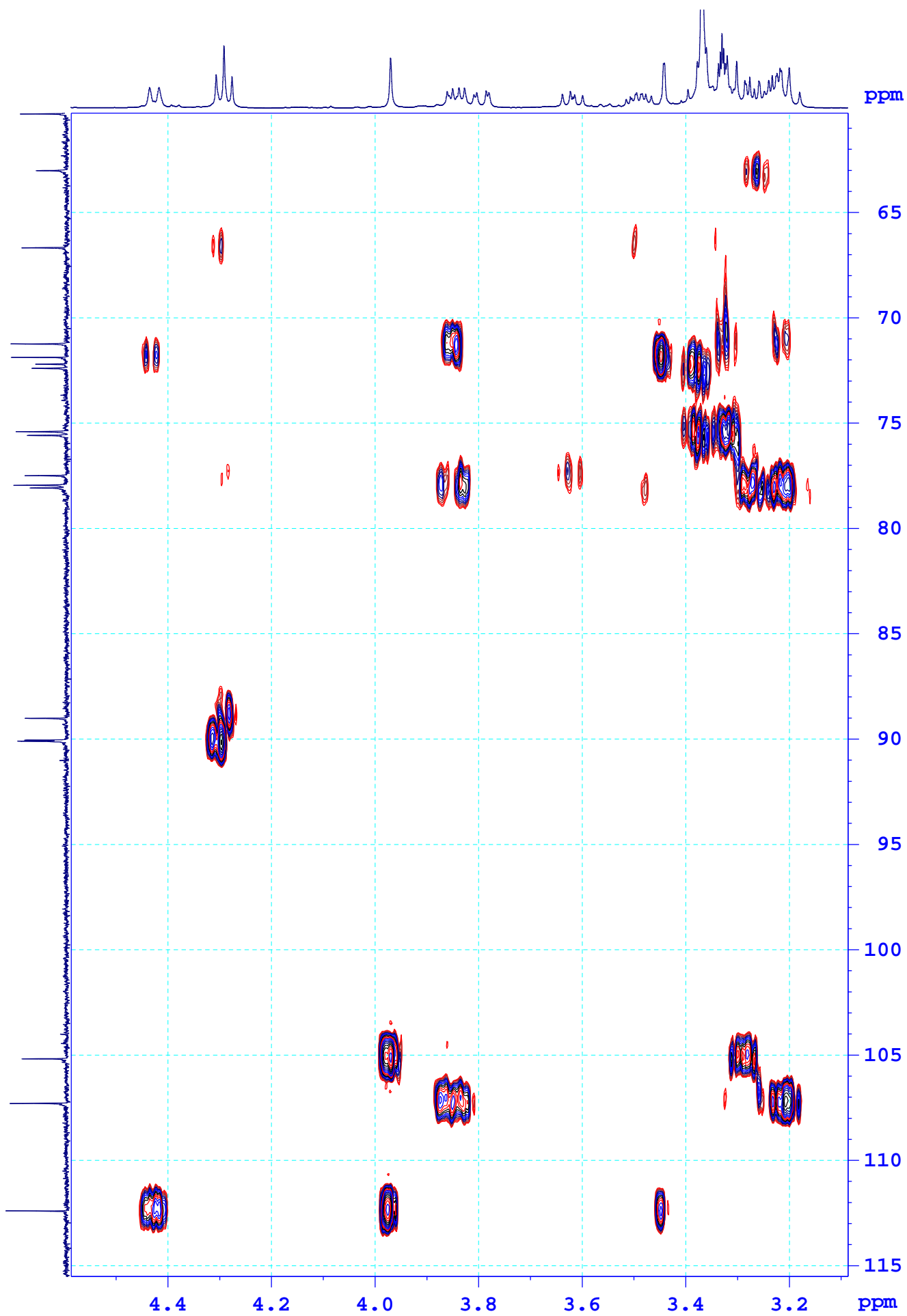

*CB6-MeOD-HMBC*

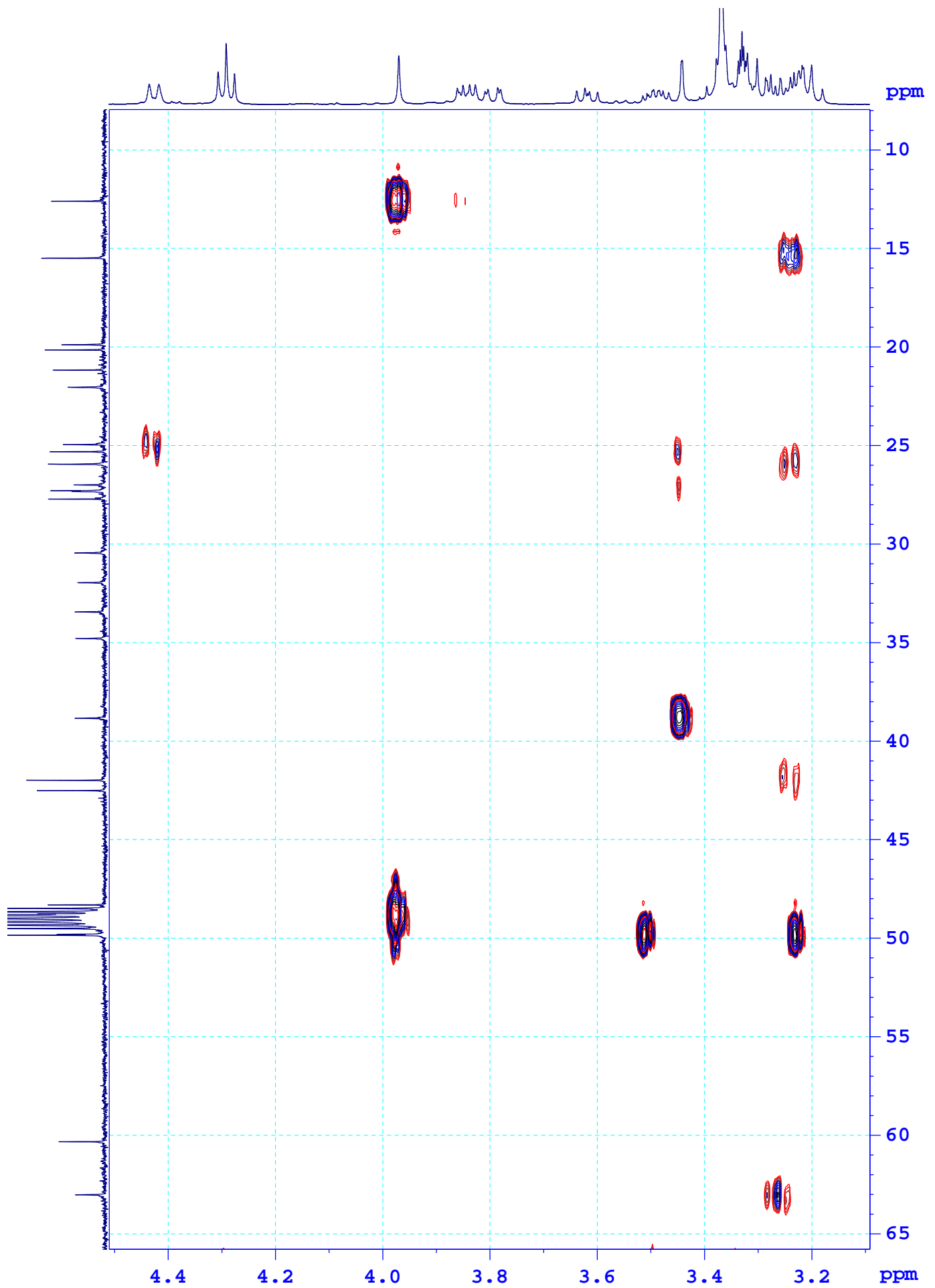

*CB6-MeOD-HMBC*

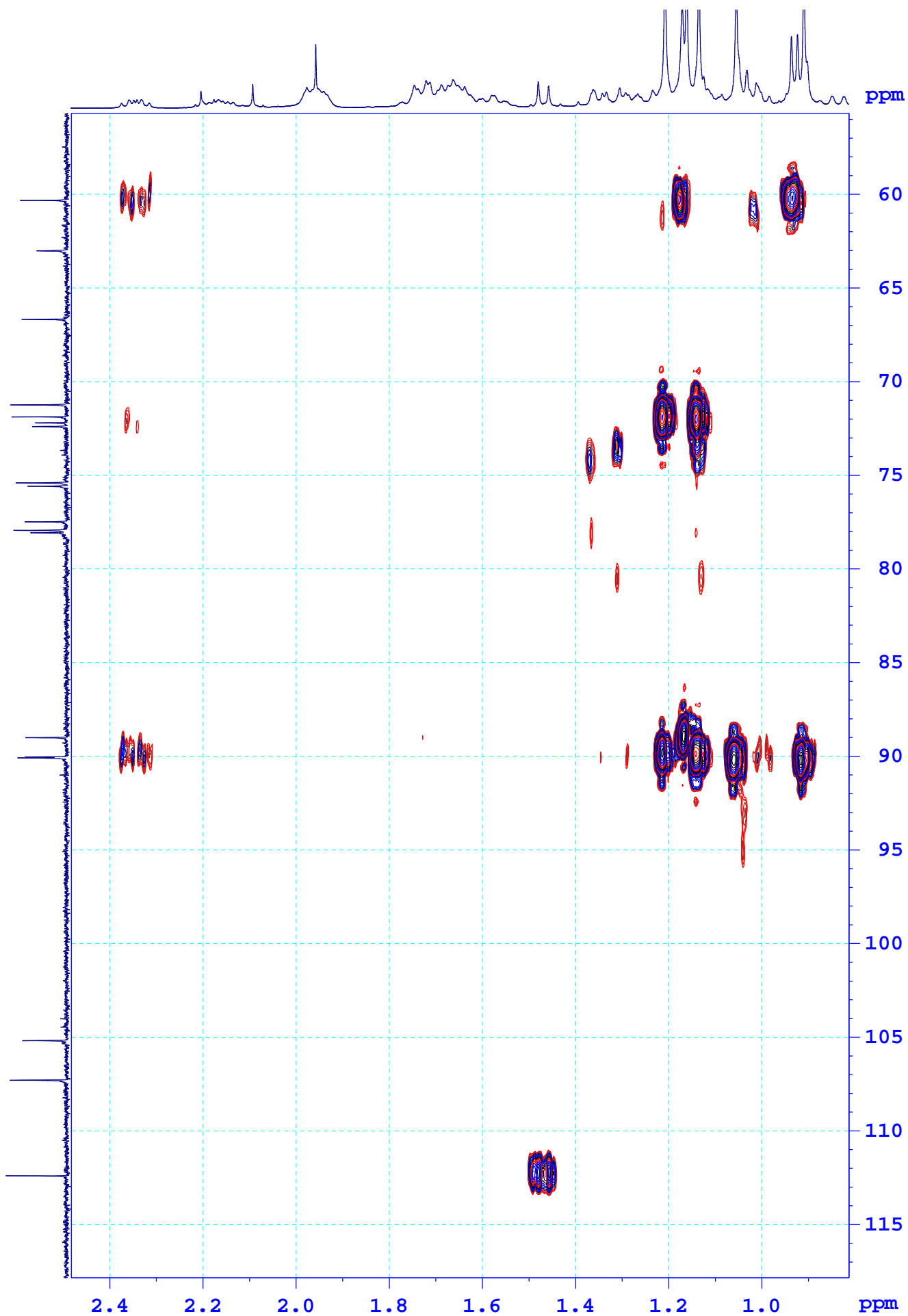

*CB6-MeOD-HMBC*

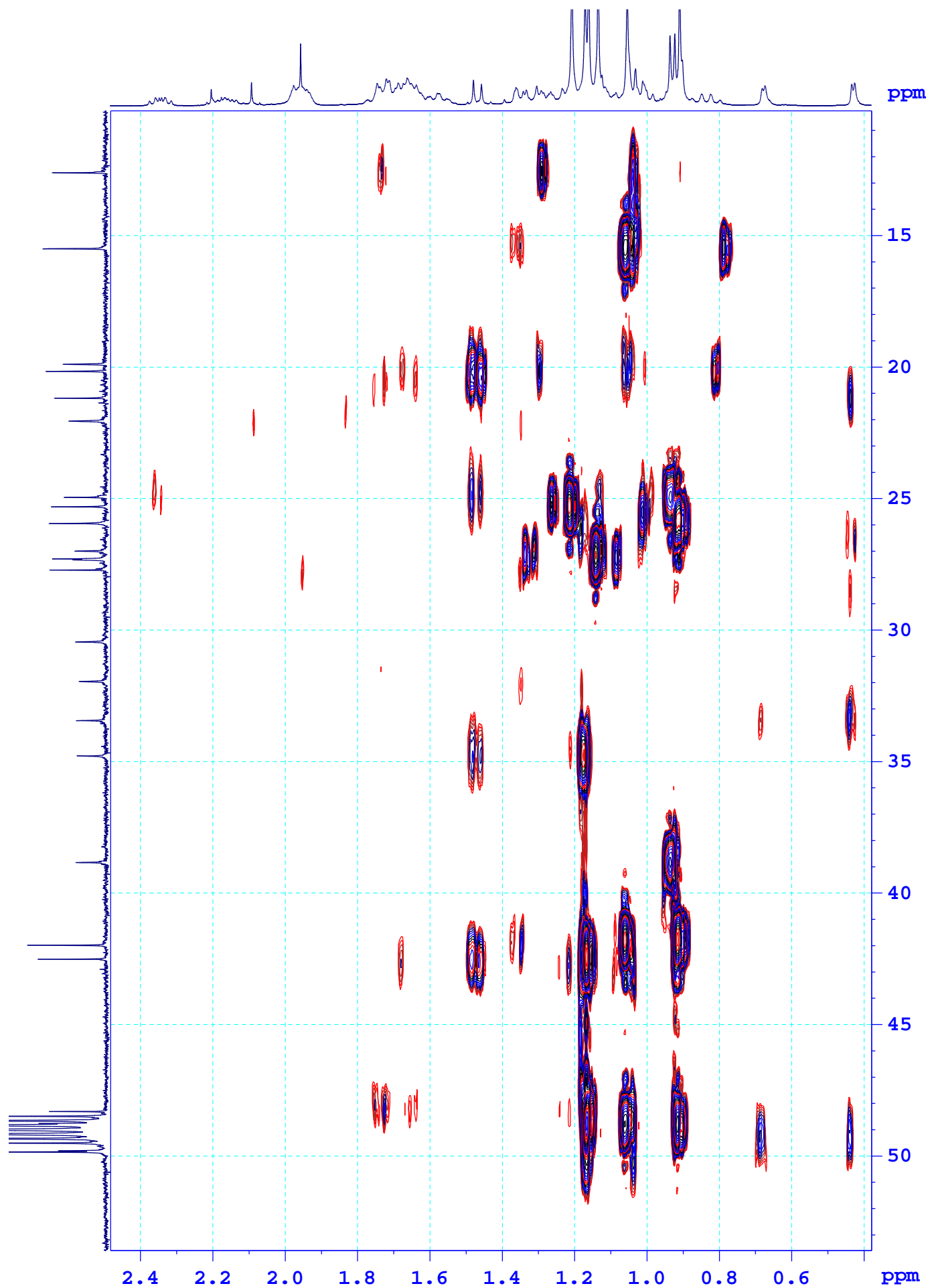

*CB6-MeOD-HMBC*

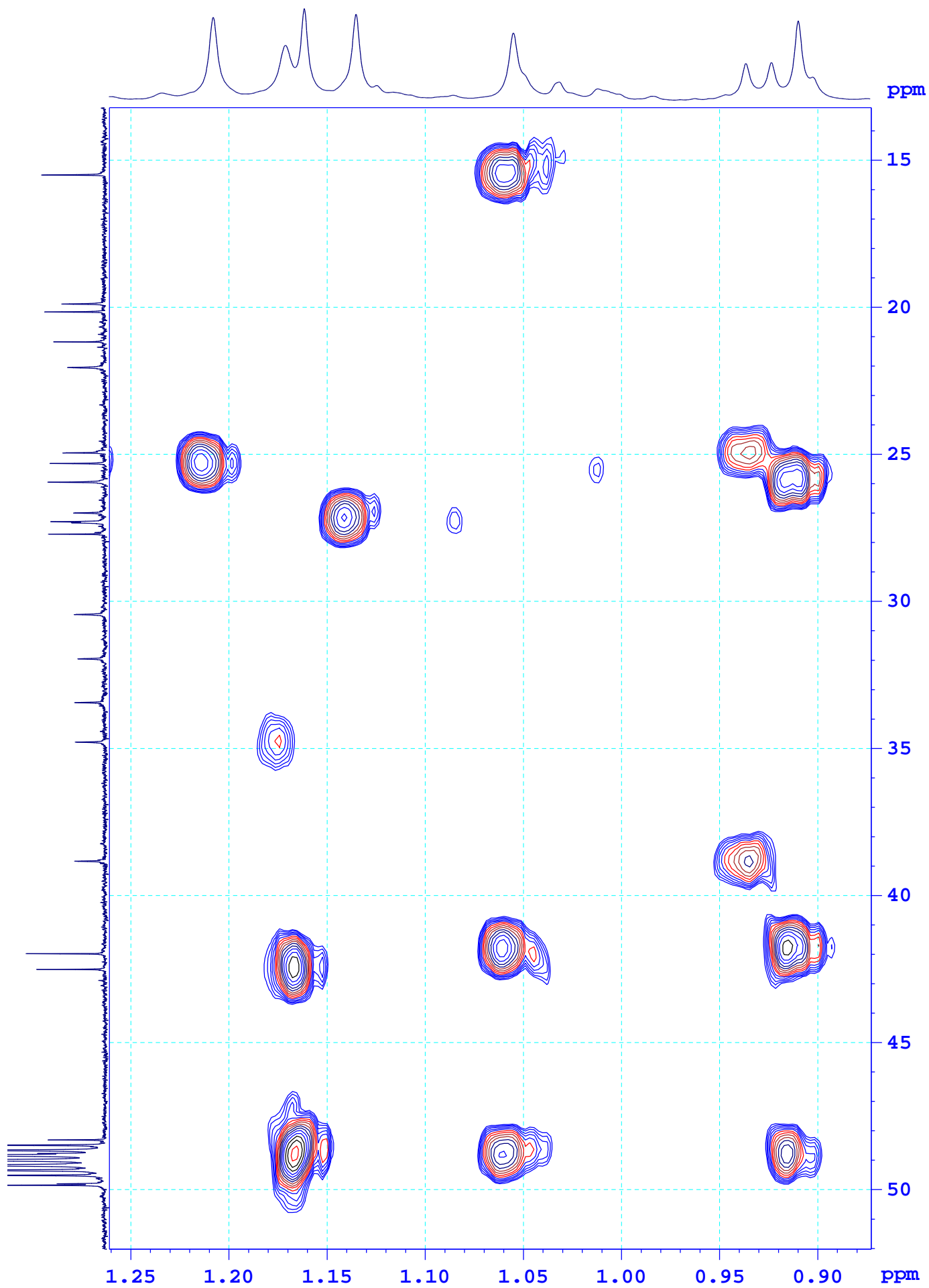

Supplement: Supplementary file 1 [file molecules-23-01083-s001.zip › Supplementary Materials_liping/Figure S16. HMBC spectrum of compound 6.pdf]

*CB6-MeOD-HSQC*

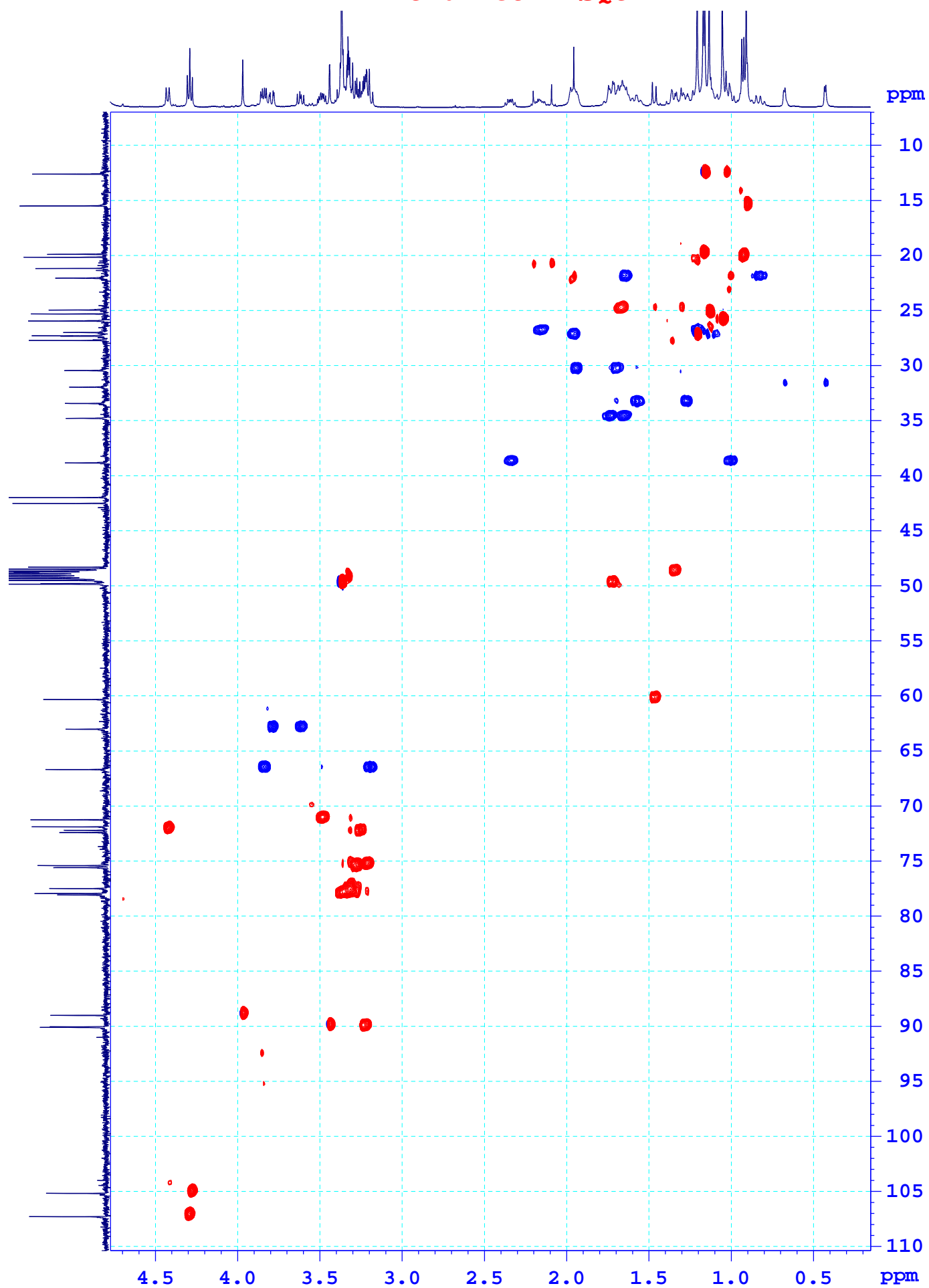

*CB6-MeOD-HSQC*

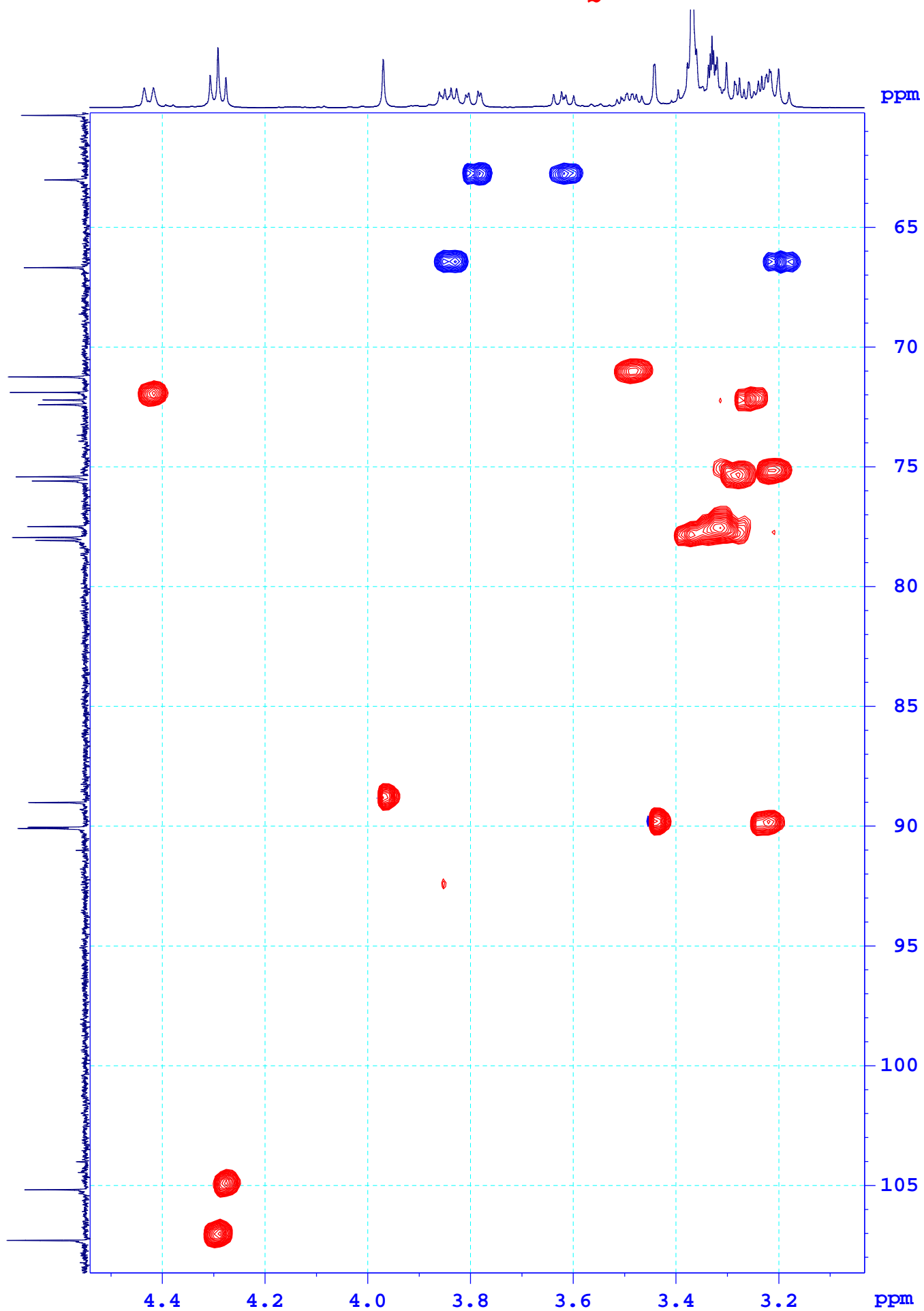

*CB6-MeOD-HSQC*

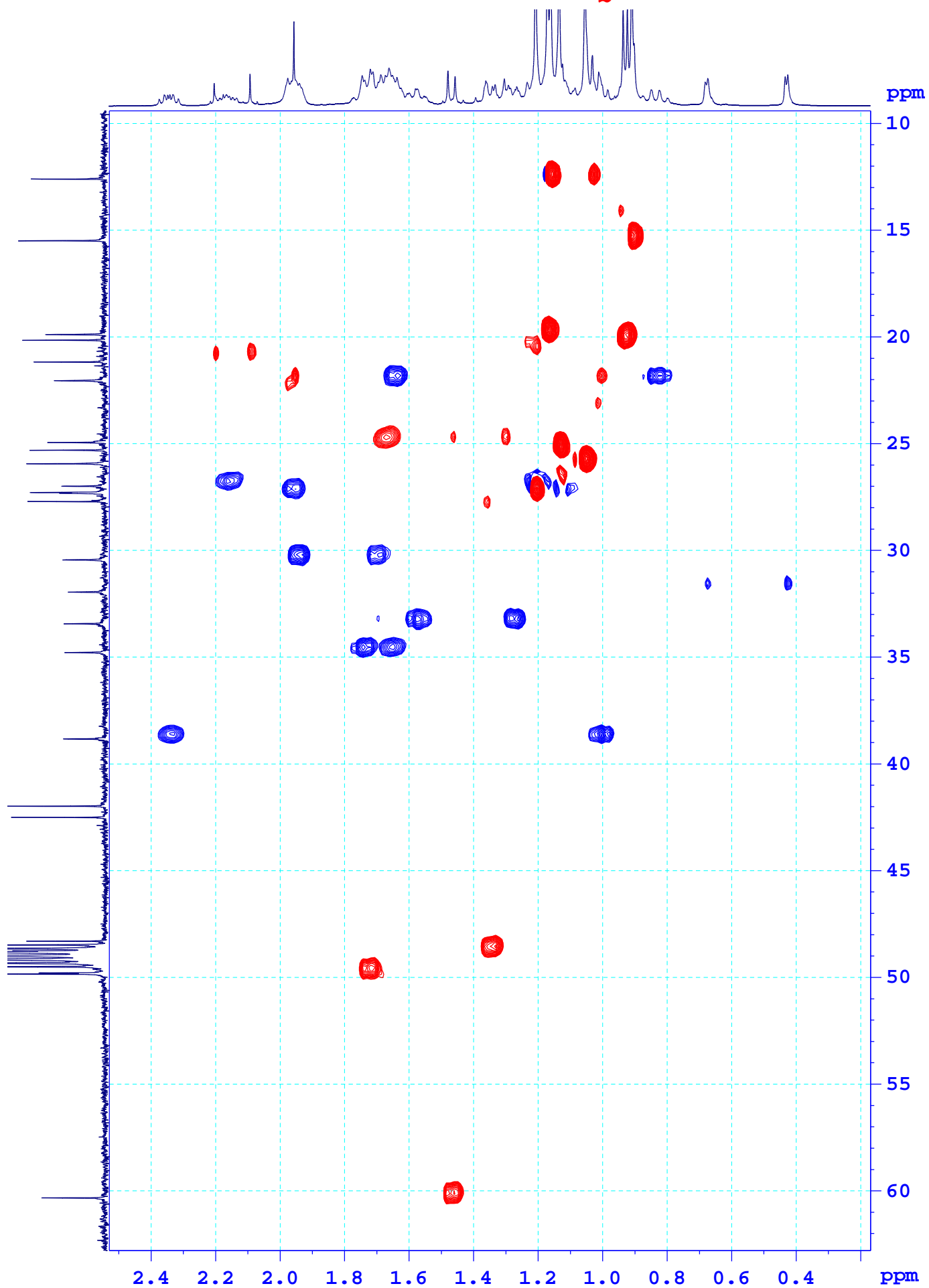

Supplement: Supplementary file 1 [file molecules-23-01083-s001.zip › Supplementary Materials_liping/Figure S17. HSQC spectrum of compound 6.pdf]

# CB7-MeOD-C13CPD &DEPT

DEPT90

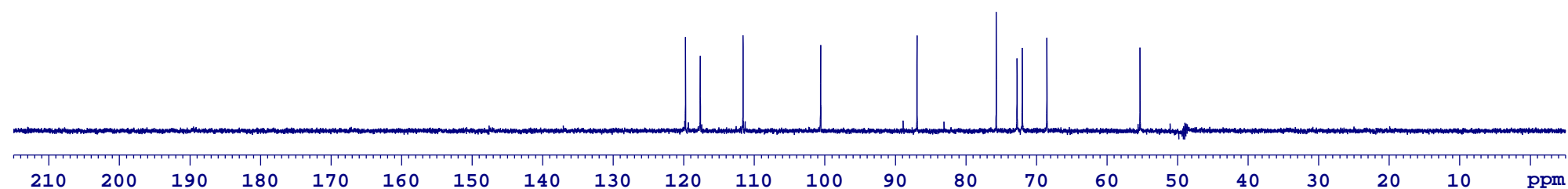

DEPT135

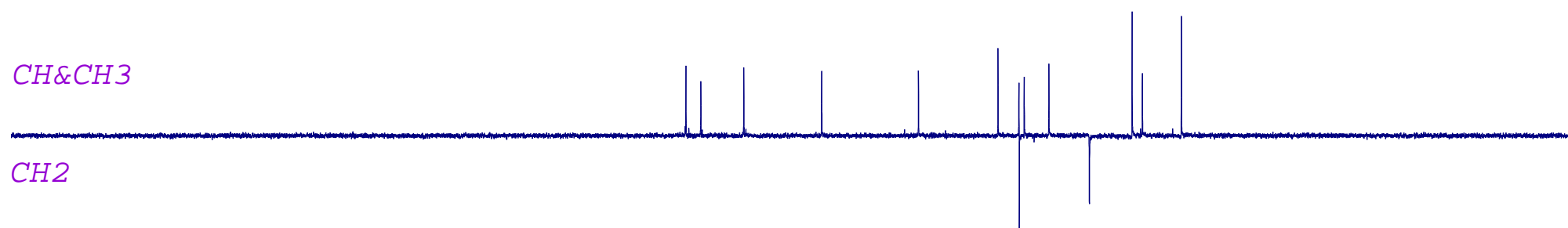

CH&CH3

CH2

C13CPD

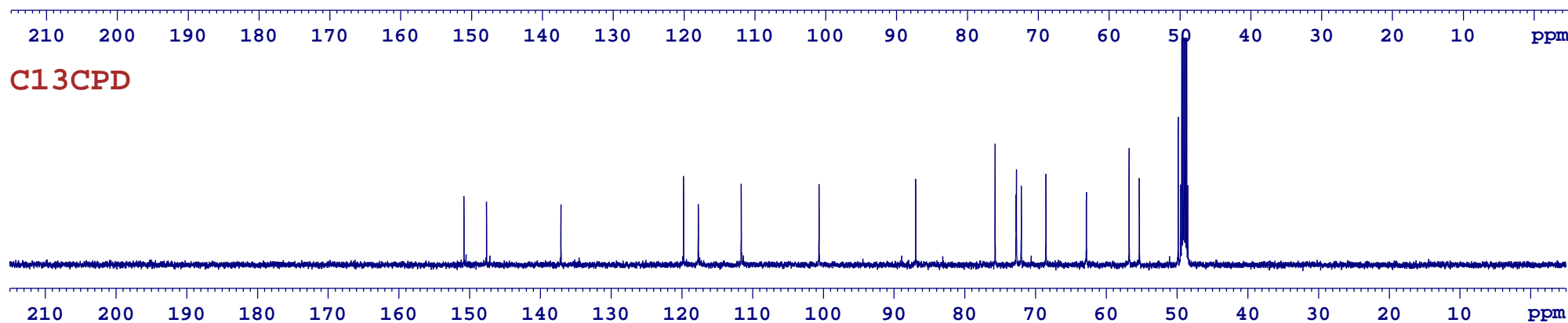

# CB7-MeOD-C13CPD &DEPT

DEPT90

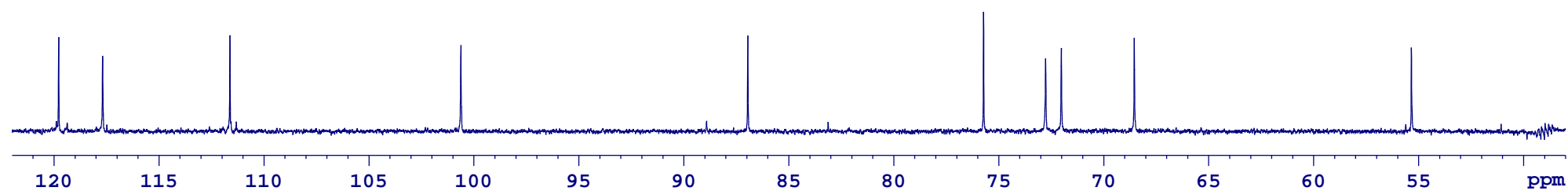

DEPT135

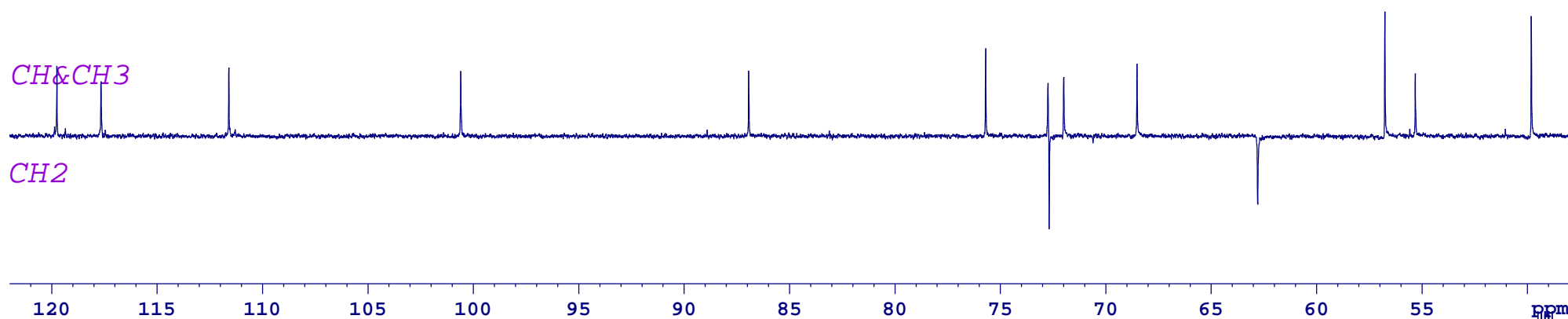

C13CPD

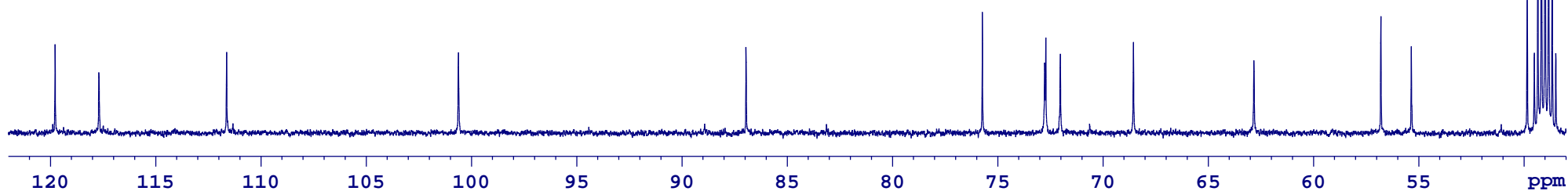

Supplement: Supplementary file 1 [file molecules-23-01083-s001.zip › Supplementary Materials_liping/Figure S22. DEPT spectrum of compound 7.pdf]

*CB7-MeOD-HMBC*

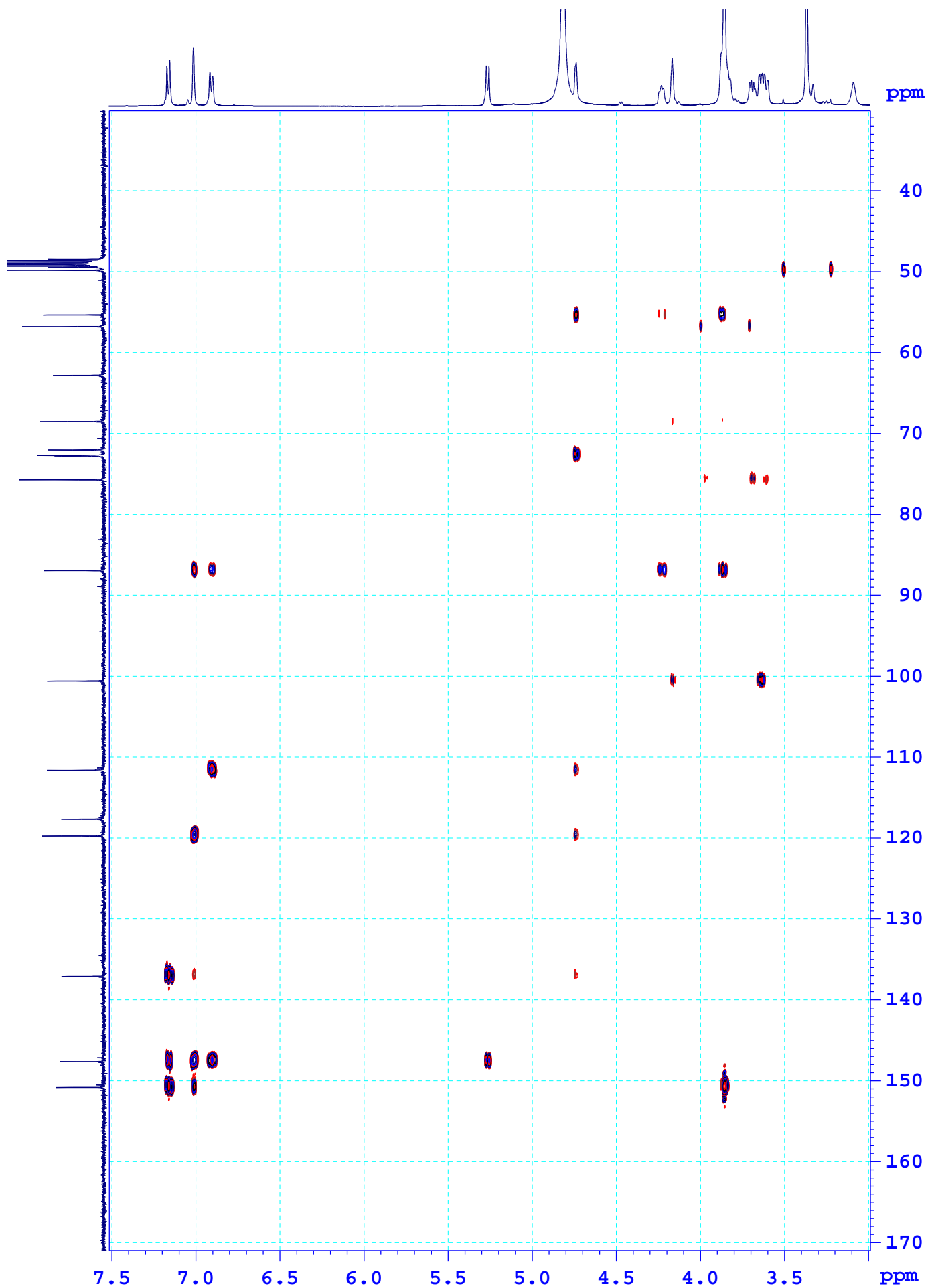

*CB7-MeOD-HMBC*

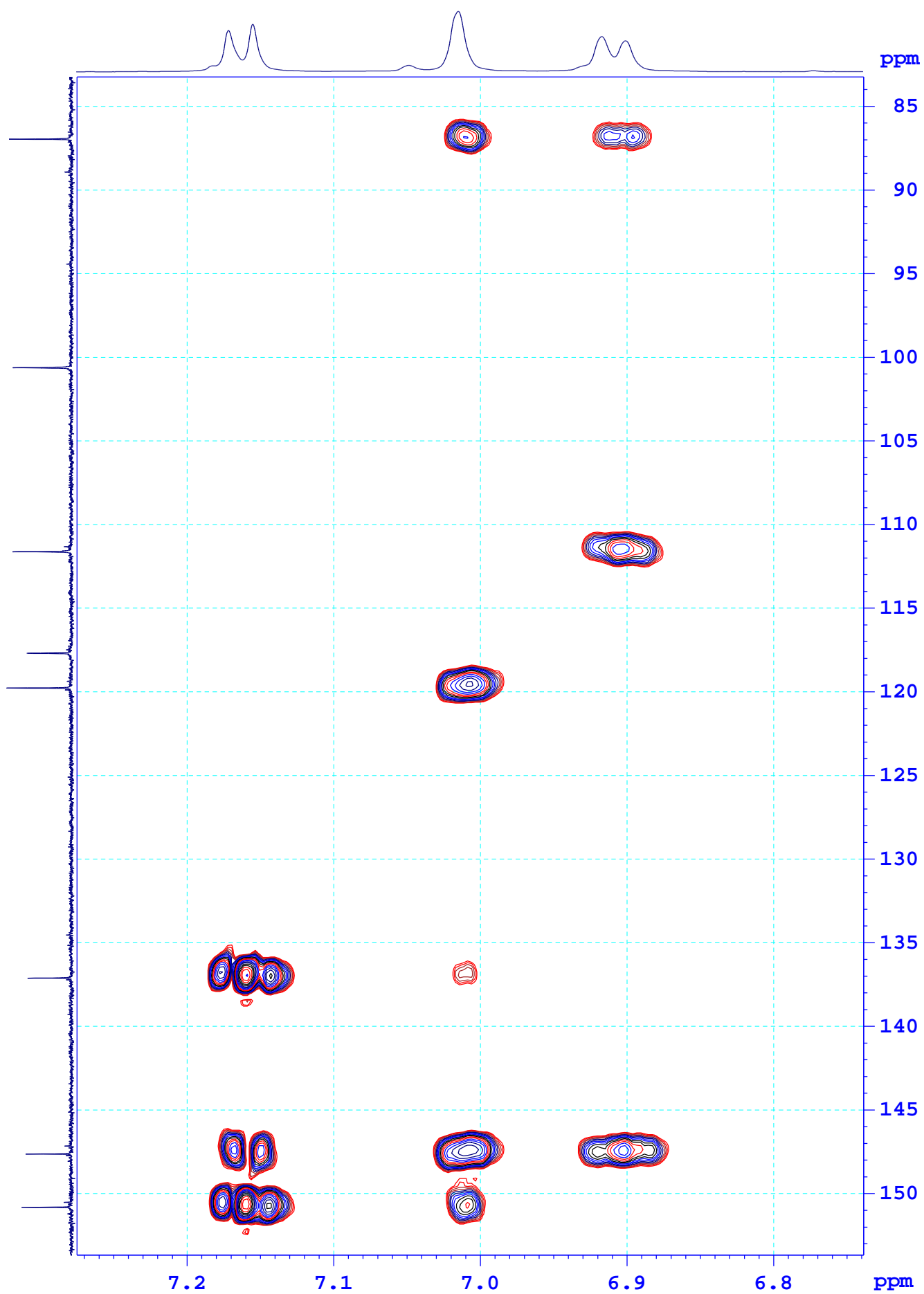

*CB7-MeOD-HMBC*

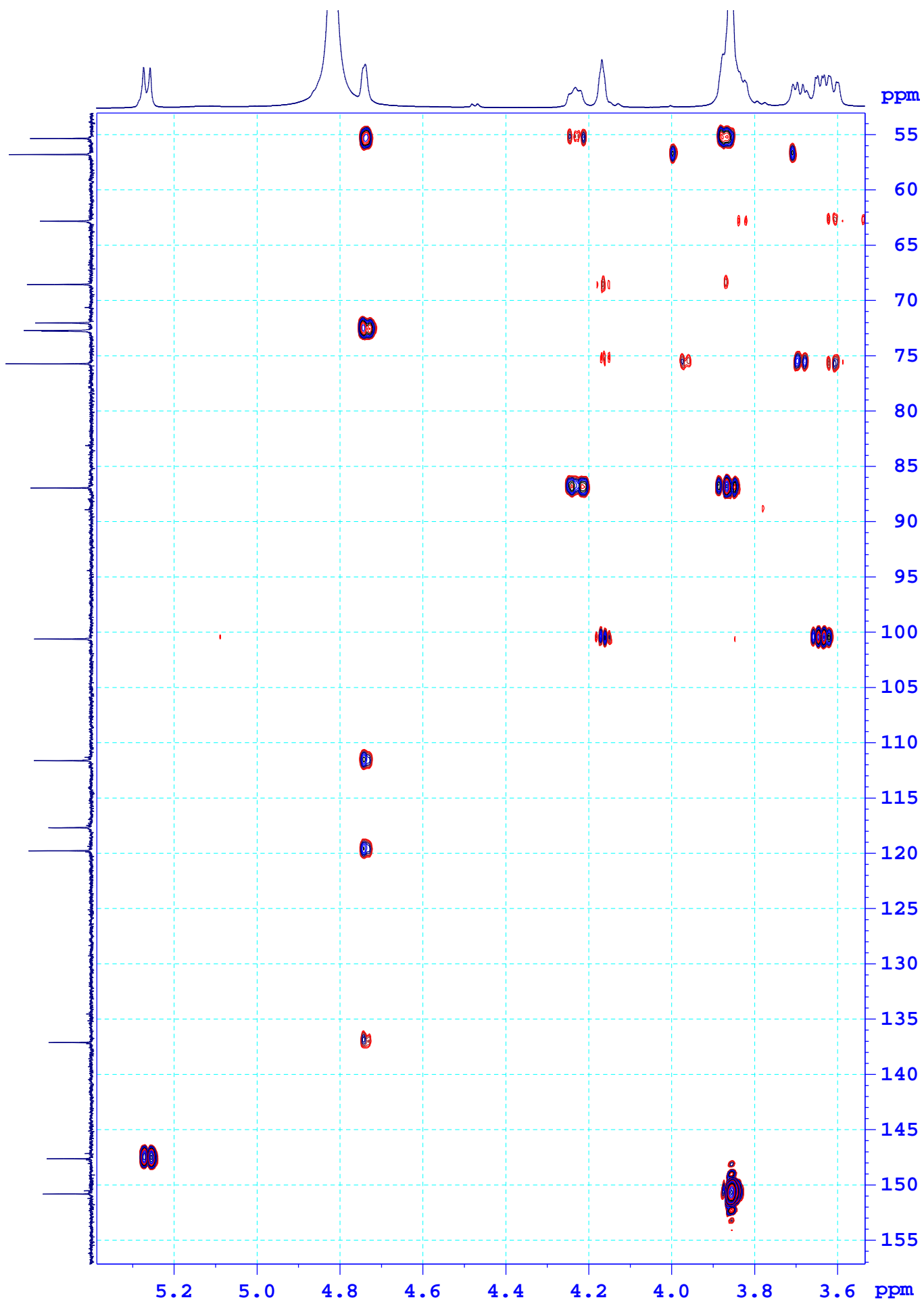

Supplement: Supplementary file 1 [file molecules-23-01083-s001.zip › Supplementary Materials_liping/Figure S23. HMBC spectrum of compound 7.pdf]

*CB7-MeOD-HSQC*

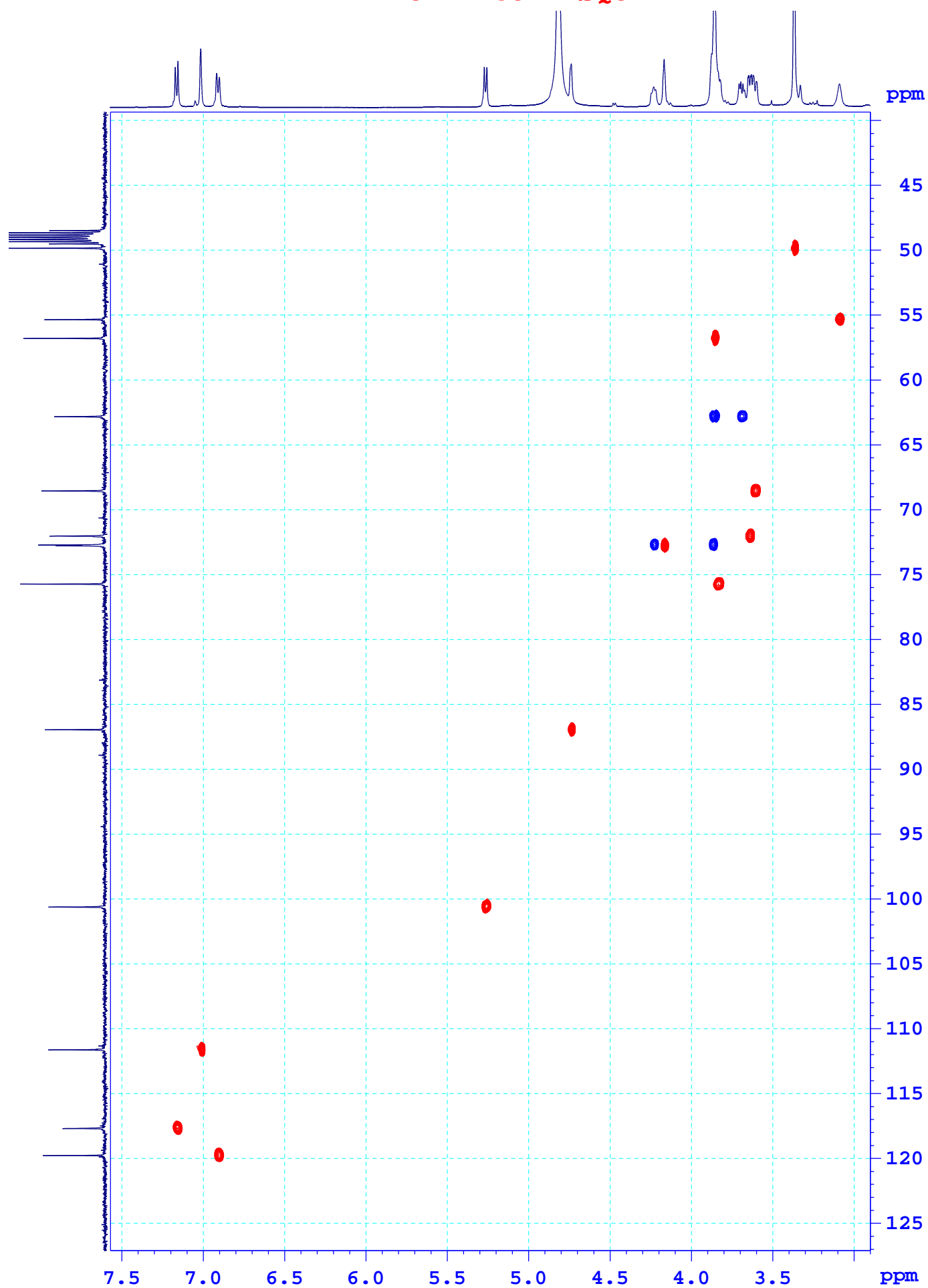

*CB7-MeOD-HSQC*

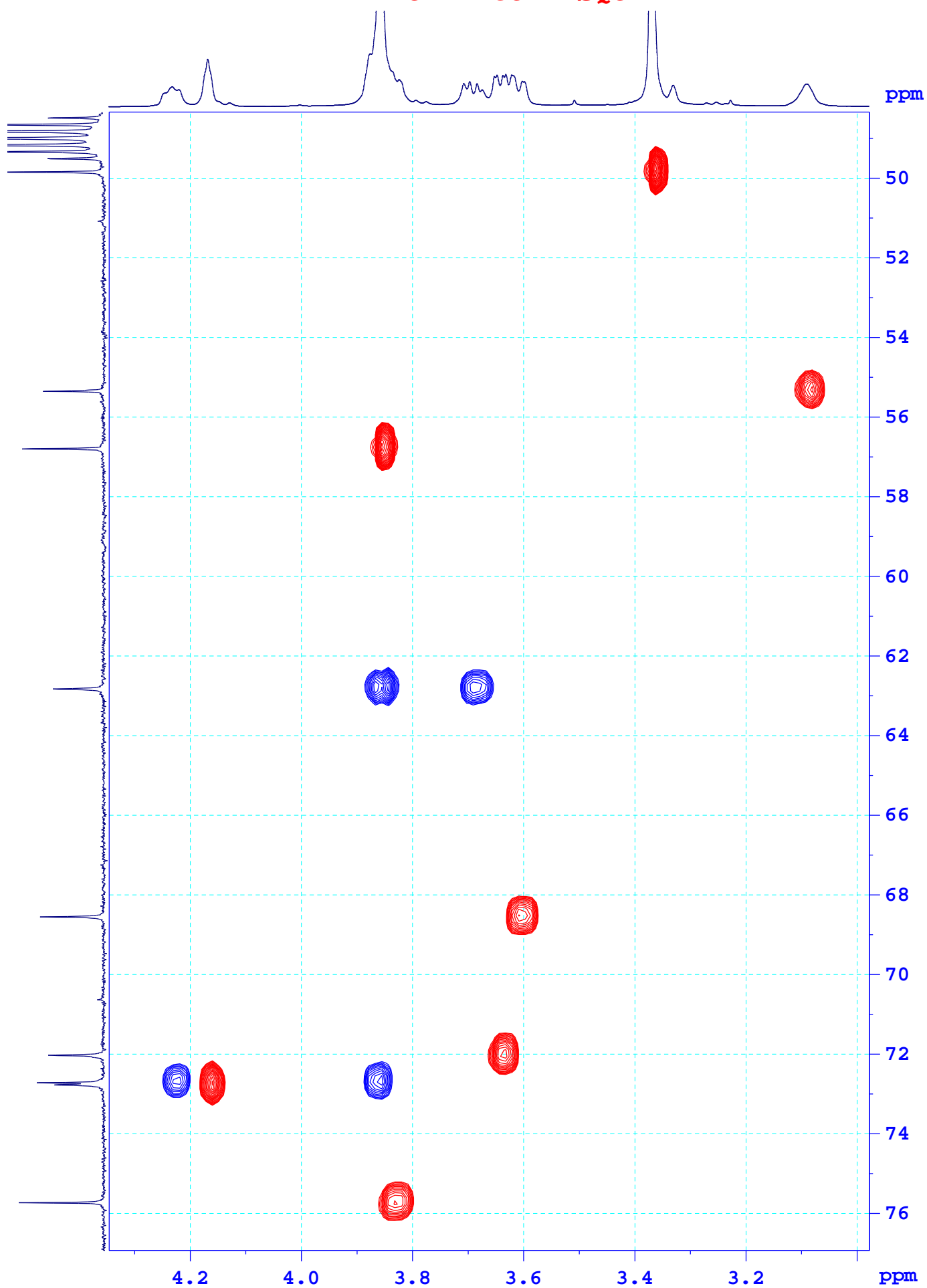

*CB7-MeOD-HSQC*

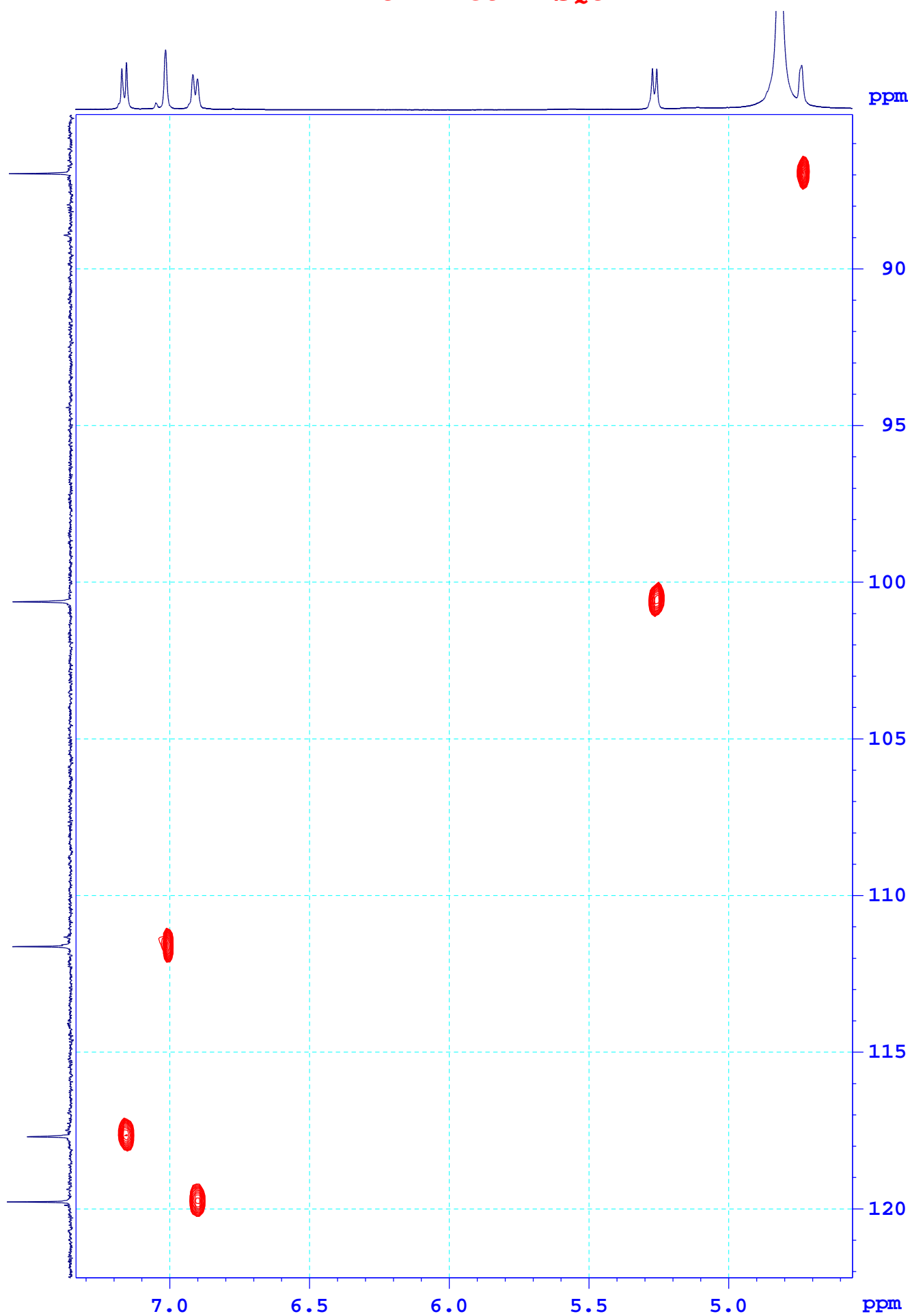

Supplement: Supplementary file 1 [file molecules-23-01083-s001.zip › Supplementary Materials_liping/Figure S24. HSQC spectrum of compound 7.pdf]
